# Discovering novel genomic regions explaining adaptation of bread wheat to conservation agriculture through GWAS

**DOI:** 10.1038/s41598-024-66903-3

**Published:** 2024-07-16

**Authors:** Amit Kumar Mazumder, Rajbir Yadav, Manjeet Kumar, Prashanth Babu, Naresh Kumar, Sanjay Kumar Singh, Amolkumar U. Solanke, Shabir H. Wani, Adel I. Alalawy, Abdulrahman Alasmari, Kiran B. Gaikwad

**Affiliations:** 1 Division of Genetics, ICAR-Indian Agricultural Research Institute, New Delhi, 110012 India; 2 National Institute for Plant Biotechnology, New Delhi, 110012 India; 3 Mountain Research Centre for Field Crops, Khudwani, 192101 India; 4 Sher-E-Kashmir University of Agricultural Sciences and Technology-Kashmir (SKUAST-K), Srinagar, Jammu-Kashmir India; 5 Department of Biochemistry, Faculty of Science, University of Tabuk, Tabuk, Saudi Arabia; 6 Department of Biology, Faculty of Science, University of Tabuk, Tabuk, Saudi Arabia

**Keywords:** Genome-wide association studies (GWAS), Conservation agriculture, Physiological adaptations, Chlorophyll fluorescence, Wheat, Agricultural genetics, Plant breeding, Plant stress responses

## Abstract

To sustainably increase wheat yield to meet the growing world population’s food demand in the face of climate change, Conservation Agriculture (CA) is a promising approach. Still, there is a lack of genomic studies investigating the genetic basis of crop adaptation to CA. To dissect the genetic architecture of 19 morpho-physiological traits that could be involved in the enhanced adaptation and performance of genotypes under CA, we performed GWAS to identify MTAs under four contrasting production regimes viz., conventional tillage timely sown (CTTS), conservation agriculture timely sown (CATS), conventional tillage late sown (CTLS) and conservation agriculture late sown (CALS) using an association panel of 183 advanced wheat breeding lines along with 5 checks. Traits like Phi2 (Quantum yield of photosystem II; CATS:0.37, CALS: 0.31), RC (Relative chlorophyll content; CATS:55.51, CALS: 54.47) and PS1 (Active photosystem I centers; CATS:2.45, CALS: 2.23) have higher mean values in CA compared to CT under both sowing times. GWAS identified 80 MTAs for the studied traits across four production environments. The phenotypic variation explained (PVE) by these QTNs ranged from 2.15 to 40.22%. Gene annotation provided highly informative SNPs associated with Phi2, NPQ (Quantum yield of non-photochemical quenching), PS1, and RC which were linked with genes that play crucial roles in the physiological adaptation under both CA and CT. A highly significant SNP AX94651261 (9.43% PVE) was identified to be associated with Phi2, while two SNP markers AX94730536 (30.90% PVE) and AX94683305 (16.99% PVE) were associated with NPQ. Identified QTNs upon validation can be used in marker-assisted breeding programs to develop CA adaptive genotypes.

## Introduction

Bread wheat (*Triticum aestivum* L.) stands as the most extensively grown and second most consumed crop globally which provides a fifth of global food calories and proteins^1^. With the world population expected to reach 9 billion by 2050, there is a pressing need to enhance the annual grain yield growth from the current 1% per year to 1.6% per year to meet the demands of such a rapidly increasing population^2,3^. However, achieving this advancement is hindered by significant challenges posed by both biotic and abiotic stress factors. Consequently, directing efforts towards enhancing wheat in specific mega-environments becomes crucial to address the unique requirements of different populations. Challenges in wheat production arise from abiotic stress factors like heat, drought, and unexpected rainfall during flowering and grain maturity. Of these, heat stress proves to be the most formidable, significantly diminishing grain yield^4^. According to Tesfaye et al.^5^, the annual average maximum temperature may rise by 1.4–1.8 degrees Celsius in 2030 and 2.1–2.6 ℃ in 2050, increasing the number of heat-stressed places by 12% in 2030 and 21% in 2050 in South Asia. According to projections made by Ortiz et al.^6^, by 2050, approximately half of India’s Indo-Gangetic Plains (IGP) may no longer be suitable for growing wheat. Therefore, in the IGP region, the most important element in determining wheat productivity is the unpredictable variation of temperature in March. Warming causes anthesis and maturity to occur earlier, which decreases crop output by shortening the growing season, decreasing the number of kernels per spike, and reducing the weight of the kernels^7,8^. The most delicate plant growth stages occur around anthesis in several crops, including wheat^9,10^. Recurrence of extreme weather patterns, such as sudden downpours of rain and hailstorms that cause crop lodging, has made the issues even more difficult. According to a thorough analysis of wheat yield from 208 districts conducted by Gupta et al.^11^, between 1981 and 2009, wheat yield decreased by 5.2% due to global warming, and wheat yields decreased by 2–4% for each 1℃ increase in the average daily maximum and lowest temperatures. Most wheat fields in India currently experience somewhat above-average temperatures, particularly during the important grain-filling stage of growth; even a tiny temperature increase will substantially impact yield.

Crop development that is moderated by climatic fluctuations can be substantially addressed by either modifying the production environment via the use of stress-reduction techniques or by choosing varieties with appropriate flowering times and duration^12^. There is a need to integrate agronomic management and responsive genotypes to rectify the problems. Although there has been progress in understanding the phenological traits under unfavourable temperature conditions, knowledge of crop plants’ overall behaviour under ideal conditions is still lacking^13^. Extreme events are predicted to occur more frequently as a result of climate change, making this understanding crucial. Reminders of these events can be found in the wheat-growing seasons of 2014–15, 2015–16, and 2021–22^14^. Predictive breeding strategies and production technologies become increasingly complex and challenging to design under such circumstances. Identification of physiological and/or phenological variables that contribute to yield improvement over time can shed some information on the pattern of adaptation to changing climatic conditions.

One effective strategy is conservation agriculture (CA), which involves retaining crop residue and practising zero tillage^15–17^. This approach ensures enhanced soil structure, optimal soil temperature, preserved soil moisture, and improved soil biodiversity^18,19^, increased soil organic carbon, root penetration, and reduced subsoil compaction^20,21^. Studies indicate that CA consistently yields better grain productivity compared to conventional tillage (CT) practices, both in stress and non-stress environments. Devkota et al.^22^ reported a substantial increase of 62% in wheat grain production under CA compared to CT in their study. CA-based cropping systems also ensure increased economic profitability, better adaptation to heat and water stress, and reduced emissions of greenhouse gases^23^. However, there is a notable gap in research regarding how plants adapt to this optimal environment at the genetic level and the lack of genotypes with specific adaptability for CA, since genotypes bred for CT practices do not always perform equally under CA^19^. Consequently, there is a pressing need for comprehensive investigations to unravel the phenological and morpho-physiological changes occurring in plants that facilitate their adaptation to conservation agriculture, leading to improved grain yields. For yield under zero-tillage conditions, three specific regions were identified on chromosomes 2D (wPt3728-cfd44 and gmw484-wmc27) and one on 5B (wmc99-wPt2373) in a study by Trethowan et al.^24^.

To deepen our understanding of such adaptation mechanisms, a thorough examination of the genetic basis, involving the dissection of traits and identification of chromosomal regions controlling their expression, would be highly valuable in the current era of molecular breeding^14^. A key QTL on chromosome 7D (7D-acc/cat-10) boosts yield and weight in wheat, showing resilience to late-season heat and drought stress. It accounts for 19.6% yield variation in stress trials, making the linked marker a potential candidate for selection in other populations^25^. Significant marker-trait associations (MTAs) were found on chromosomes 2A and 2B, explaining 7–25% of trait variation^26^. GWAS conducted for irrigated, stressed, and combined environments, along with trait per se and stress indices, revealed QTL hotspots on chromosomes 2A (54–70 cM) and 2B (75–82 cM). QTLs previously identified for grain filling rate (GFR) and grain filling duration (GFD) were validated in Indian breeding material under conservation agriculture. Notably, two SSR markers, XCfd42 and Xwmc500, accounted for approximately 6% and 1% variation in GFR, respectively^27^. Genome-wide association studies (GWAS) have enabled the identification of MTAs for various phenological and morpho-physiological traits such as heading date, grain filling duration, plant height, spike traits, kernel weight, grain yield, canopy temperature, chlorophyll content and normalised difference vegetative index (NDVI) in wheat^28–33^. Keeping previous knowledge and current circumstances in consideration, the current study was aimed at finding important morpho-physiological traits that increase genotype adaptation for CA environments, as well as MTAs for the traits under differing production regimes, using GWAS . It will provide new insights into the mechanisms by which wheat adapts to conservation agriculture (CA), ultimately leading to an improved grain yield compared to conventional tillage (CT). This will be the first report where we have identified key physiological traits and putative candidate genes responsible for the adaptation of wheat to CA environments.

## Materials and methods

### Experimental materials

The plant material used in the current study comprised 183 advanced breeding lines with diverse parentages (Supplementary Table 1) along with five commercially cultivated varieties viz., HD3226, DBW187, HD3086, HD3298 and HI1621. The varieties HD3226, DBW187 and HD3086 were used as checks for timely sown conditions and varieties HD3298 and HI1621 for late sown conditions. The use of advanced breeding lines for association studies also enables their direct use in the transfer of identified QTNs and their relevance in the cultivar development process^34^.

### Location, experimental site and production environments

The field experiments were conducted at the Research Fields of ICAR-Indian Agricultural Research Institute, New Delhi. The location is situated at N28°38′24.0252″, E77°10′26.328″, with an altitude of 228.61 m (750 ft) above sea level in the subtropical region of the Northwestern Plain Zone of India. The experiment was designed with four contrasting production environments: conventional tillage timely sown (CTTS), conventional tillage late sown (CTLS), conservation agriculture timely sown (CATS), and conservation agriculture late sown (CALS). The experimental field is maintained under CA (zero tillage with residue retention) since 2008. For timely sown conditions, the trials were planted in the second week of November in the Rabi season 2022–23. In contrast, for late sown conditions, the planting was done in the third week of December. This scheduling aimed to subject the genotypes to terminal heat stress during their growth.

### Field experiment and phenotyping

The experiment was laid in an augmented block design comprising all 183 advanced breeding lines and 5 checks in 8 blocks in all four production environments. Augmented block design is very helpful in experiments where a huge number of genotypes need to be screened but land resources are scarce due to which replication of all genotypes is not possible. In an augmented block design, only the checks are replicated across the blocks while test genotypes are planted without replication as new entries. The experimental materials were planted when adequate moisture was available in the soil. In each production environment, genotypes were grown with a plot size of 4.8m^2^ (6 rows of 4 m length). The fields were irrigated in regular intervals depending upon rainfall and moisture availability in the fields. The cultivation of the crop adhered to recommended standard cultural and agronomic practices to ensure proper growth and development of the crop.

Physiological traits included chlorophyll fluorescence parameters viz., ϕII quantum yield (Phi2) and non-photochemical quenching (NPQ), active photosystem I center (PS1), relative chlorophyll (RC, as SPAD measurement), canopy temperature differential (CTD, in ℃), leaf thickness (LT, in mm) and leaf angle (LA, in °). All the physiological traits were observed using PhotosynQ MultispeQ v2.0^35^ in a non-destructive way in the field. The agro-morphological traits observed in the present study included days to heading (DTH, when 50% of plants in the plot achieve heading, Zadoks stage 59^36^), days to maturity (DTM, when 50% of plants in the plot achieve physiological maturity, Zadoks stage 89^36^), plant height (PH, in cm), tiller count (TC, number of productive tillers per mt row), spike length (SL, in cm), spikelets per spike (SPS), grains per spike (GPS), thousand-grain weight (TGW, in g) and grain yield (GY, in q/ha). Phenological observations were recorded on five randomly selected plants from each plot in all four production environments and grain yield was taken by harvesting of whole 4.8m^2^ plot. In addition to the morpho-physiological traits, grain geometric parameters viz., grain length (GL, in mm), grain breadth (GB, in mm) and grain surface area (GSA, in mm^2^) were also measured using scanned images of threshed grains obtained from two randomly picked spikes by analysing the images in SmartGrain software v1.3^37^.

Plant adaptation to diverse environments involves a multifaceted interplay of physiological and metabolic adjustments orchestrated by alterations in gene expression. Among these adaptations, photosynthetic efficiency emerges as a critical trait profoundly influenced by environmental factors, ultimately dictating the performance of plant genotypes. The quantum yield of photosystem II (Phi2) serves as a pivotal indicator of photosynthetic efficiency, reflecting the extent to which incident light is harnessed in photochemical reactions—a parameter with profound implications for biomass yield optimization. In contrast, the quantum yield of non-photochemical quenching (NPQ) denotes the proportion of incident light dissipated as heat, which is an important photoprotective process that protects the photosystem from damage caused by excess light energy. In addition, a higher value of relative chlorophyll content and active photosystem I reaction centers indicate an optimized light capture and electron transport. Furthermore, a cooler canopy temperature (indicated by higher value of CTD—canopy temperature depression) indicates efficient transpiration and potentially higher stress tolerance, while a warmer canopy temperature (lower CTD value) suggests limitations in water uptake or transpiration. To deepen our comprehension of how these physiological traits influence grain yield, we have selected agro-morphological traits to establish significant correlations between yield component traits and the aforementioned physiological traits.

### Statistical analysis

Statistical analyses were performed using the mean values of the observations recorded on ten randomly selected plants from each plot in all four production environments. Analysis of variance (ANOVA) was performed for Augmented block design in R studio software v4.3.1 using the “AugmentedRCBD” package^38^. The statistical significance for the morpho-physiological traits was analyzed as test and check treatments for block-adjusted ANOVA. To account for the presence of GxE interactions, a combined analysis of variance was performed for the traits in the present study using the linear mixed model analysis from the lme4 package in R embedded in the META-R software^39^. The analysis was carried out considering the RCBD design and using the restricted maximum likelihood (REML) method assuming fixed effects of the genotypes, while all other terms were treated as random effects. The best linear unbiased prediction (BLUP) was calculated for tested genotypes combined across all environments to determine the variance components and the significance level of genotype, environment and GxE interactions. In addition, the coefficient of variation (CV%), least significance difference (LSD, *p* ≤ *0.05*), broad sense heritability (*H*^*2*^) and the grand mean for each trait were calculated. Mean squares of each source of variation were used to estimate the variance components for genotype (${\sigma }_{G}^{2}$), genotype Χ environment interaction (${\sigma }_{GE}^{2}$) and residual error (${\sigma }_{\varepsilon }^{2}$), respectively, and the heritabilities were estimated using the formula ${H}^{2}={\sigma }_{G}^{2}/({\sigma }_{G}^{2}+\frac{{\sigma }_{GE}^{2}}{e}+\frac{{\sigma }_{\varepsilon }^{2}}{r})$, where *e* and *r* were the number of environments and replications in each environment, respectively^40^. The correlation analysis for the traits was performed using the “corrplot” package^41^ in R Studio employing Pearson’s correlation test.

### Genotyping and quality control

The advanced breeding lines and check varieties in the association panel were genotyped using Breeders’ 35 K Axiom Array which contains 35,143 SNPs. To avoid confounding effects of spurious alleles, quality control of SNPs was performed by removing SNPs with missing data of > 20%, MAF < 5% and heterozygosity > 15% to generate the filtered SNP data using TASSEL v5.0 (Trait Analysis by Association, Evolution and Linkage) software^42^.

### Population structure and linkage disequilibrium

To determine the presence of any population stratification in the association panel, a SNP-based principal component analysis (PCA) was performed using the filtered SNP data in TASSEL v5.0. The population structure within the group of 188 genotypes was also determined using the Bayesian inference program STRUCTURE v2.3.4^43^. This software utilises a model-based clustering approach, leveraging genotype information from independent markers to infer population structure. The optimal number of populations (denoted as K) was identified using the ad-hoc delta K method^44^. Linkage disequilibrium (LD) was evaluated by calculating the correlation (r^2^) in allele frequency between marker pairs, using TASSEL v5.0 software. The LD decay was assessed by plotting pairwise LD values (r^2^) against genetic distance for the A, B, and D subgenomes, as well as the whole genome of bread wheat genotypes in our association panel using the “LDheatmap package”^45^ in R Studio.

### Genome-wide association studies and in-silico analysis

**Figure 1:**
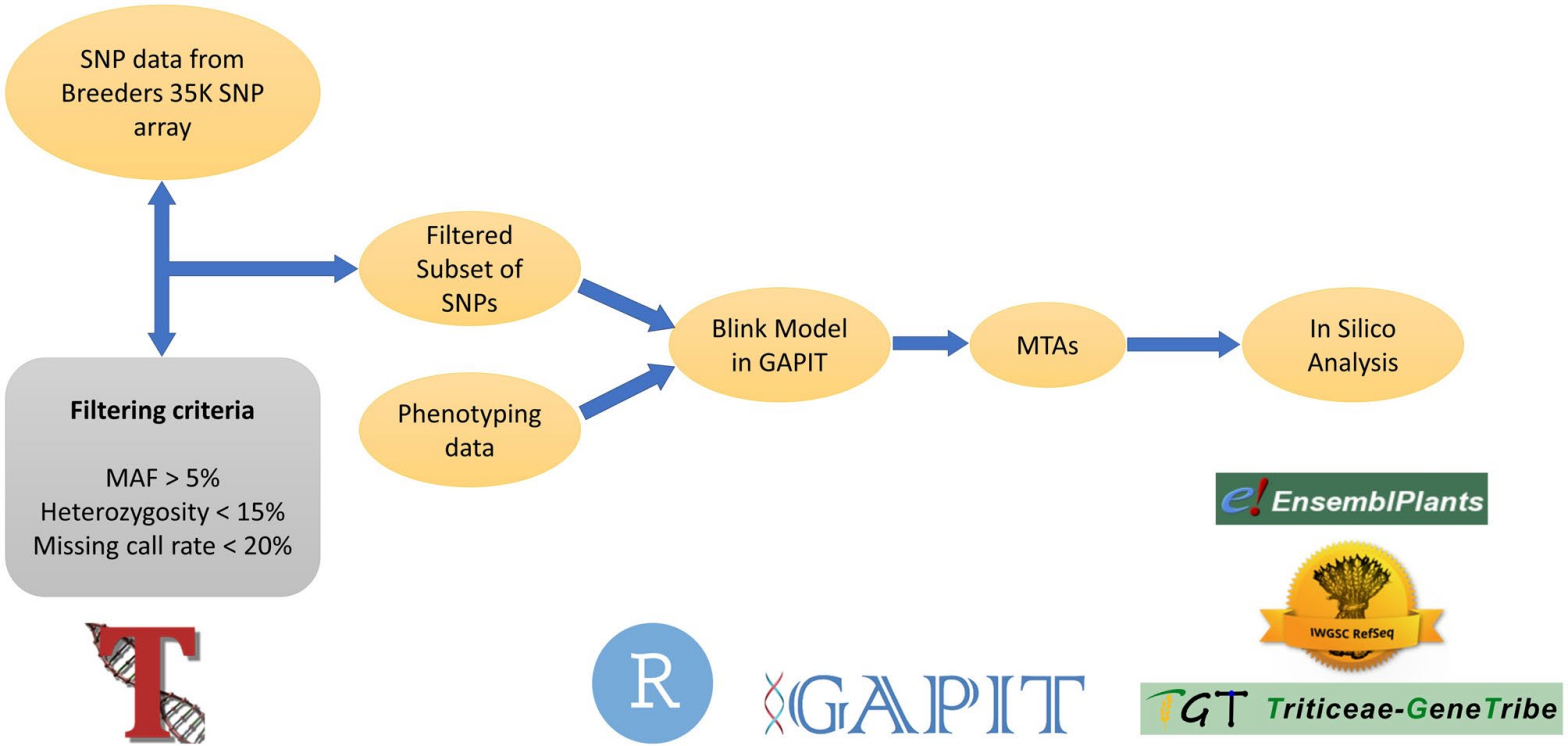
Workflow to identify MTAs for morpho-physiological traits through GWAS.

To identify significant MTAs in each production environment using the phenotypic and genotypic data, GAPIT3 package^46^ was used in R Studio applying the BLINK model^47^ for its high computing efficiency and statistical power. Besides its ability to include principal components as covariates to mitigate the risk of false positives resulting from population stratification, BLINK methodically integrates linked markers as covariates during the testing of other markers. This process is designed to unravel their link to any cryptic relationships among individuals. The markers that are linked to each other are chosen based on linkage disequilibrium, optimized concerning Bayesian information content (BIC), and subject to repeated scrutiny across multiple tests to minimize the occurrence of false negatives. Only such QTNs that had a -log_10_P value above the Bonferroni threshold of 5 were identified as significant MTAs. To identify candidate genes associated with the identified QTNs through GWAS analysis, an *in-silico* analysis was conducted. Genes located within a 1 Mb interval centred on the identified QTN were identified by searching the *EnsemblPlants* wheat database with the IWGSC Reference Sequence v1.0 assembly (https://plants.ensembl.org/Triticum_aestivum/Info/Index). While researchers typically search the entire linkage disequilibrium (LD) region to find candidate genes linked to important QTNs, we narrowed our search area to 1 Mb due to the unusually large LD blocks in our association panel. Information regarding the proteins encoded by the genes identified through gene annotation and their respective functions was sourced from the Triticeae-Gene Tribe (http://wheat.cau.edu.cn/TGT/). Only high-confidence protein-coding genes, accompanied by their Gene ID, were considered. These genes were then analyzed for their protein functions to establish a connection with the trait of interest. Subsequently, only protein-coding genes highly relevant to the expression of the specific traits under investigation were selected. The workflow for quality control of SNP data and subsequent utilisation of filtered genotypic data and phenotypic to identify MTAs followed by the *in-silico* analysis to identify putative candidate genes linked with the identified MTAs is represented in Fig. 1.

### Plant guideline statement

The use of plant and plant material in the study is in compliance with relevant guidelines and regulations.

## Results

### Phenotypic variation for *morpho*-physiological traits

**Figure 2:**
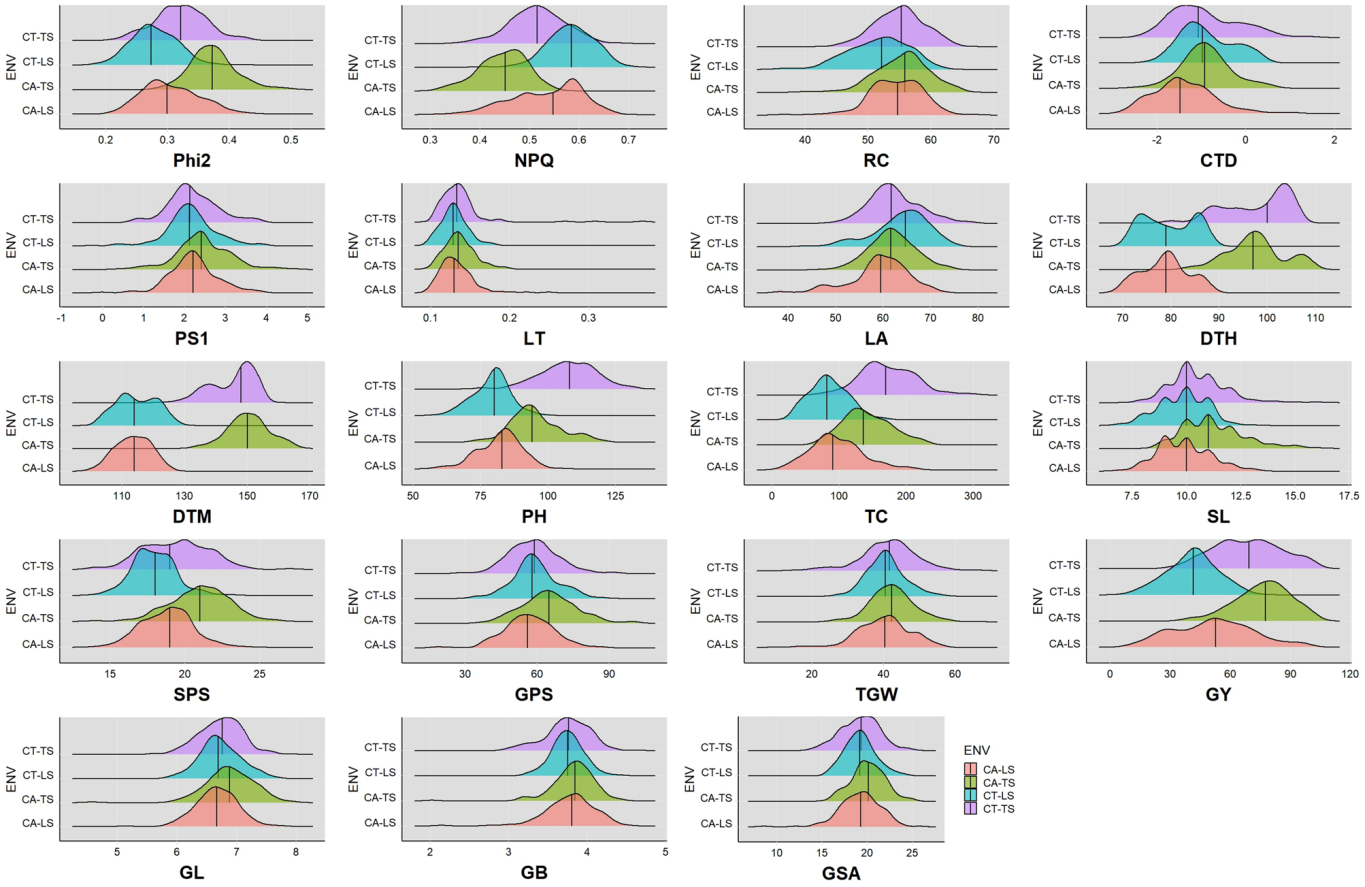
Frequency distribution of the studied morpho-physiological traits in the four contrasting production environments (CTTS, CTLS, CATS and CALS). The vertical black lines indicate the mean trait value in the respective production environment. *Phi2* PS II quantum yield, *NPQ* non-photochemical quenching, *RC* relative chlorophyll, *CTD* canopy temperature depression, *PS1* active PS I center, *LT* leaf thickness, *LA* leaf angle, *DTH* days to heading, *DTM* days to maturity, *PH* plant height, *TC* tiller count, *SL* spike length, *SPS* spikelets per spike, *GPS* grains per spike, *TGW* thousand-grain weight, *GY* grain yield, *GL* grain length, *GB* grain breadth, *GSA* grain surface area.

The genotypes in the association panel were analysed in all four production environments. The phenotypic distribution of the studied morpho-physiological traits across the four production environments is depicted as ridgeline plots in Fig. 2. The summary of descriptive statistics and block-adjusted ANOVA for the studied traits under all four production environments is given in Supplementary Tables S2, S3a–d, respectively. Through ANOVA, significant variations (**p ≤ *0.01*) were observed among the genotypes for all the morpho-physiological traits in each production environment. CT was found to be the environment with the highest variability for the studied traits as compared to CA emphasizing the ability of genotypes to adapt to CA and retain optimum and stable yields. The combined ANOVA revealed significant differences (*p ≤ *0.05*) for all test traits across environments for genotype (except PS1) as shown in Table 1. Grand means, coefficient of variation (CV), least significance difference (LSD) and broad-sense heritability (*H*^2^) are given in Table 1. The CV values ranged from 1.37% for GY to 5.30% for NPQ. For *H*^2^, the highest estimates were produced for GL (93.81%) followed by DTM and DTH at 85.94% and 85.87%, respectively. Furthermore, highly significant differences (***p ≤ *0.001*) for all tested traits (except GL) were observed due to the GxE interactions (Table 1).

**Table 1:** Combined ANOVA for morpho-physiological traits across the four production environments.

Trait^a^	Source of variation^b^								
	Genotype variance	GenxEnv variance	Residual variance	Genotype significance	GenxEnv significance	Grand mean	LSD	CV	Heritability
Phi2	0.000149952*	0.001632408***	3.09E–05	0.010516528	6.05E–29	0.319545576	0.029191638	1.740734274	0.268240795
NPQ	0.000308049***	0.001312702***	0.000755876	0.000156311	1.63E–16	0.51866289	0.038759627	5.300783999	0.46685003
RC	9.627737559***	15.21009808***	0.573855706	4.55E–23	5.98E–24	54.16737398	4.677234473	1.398503541	0.715912991
CTD	0.029987899*	0.402816441***	0.000479121	0.026147208	7.45E–141	− 1.03233296	0.422332077	2.120327808	0.229428442
PS1	9.12E-15^ ns^	0.376195342***	0.001995769	0.999994383	1.01E–103	2.255475233	2.65E–07	1.980692346	9.69E–14
LT	0.000139772***	0.000340982***	4.13E–06	1.68E–17	9.87E–59	0.133030784	0.020346089	1.52825208	0.62080495
LA	3.255278381**	26.92564585***	1.914575227	0.004536538	1.63E–28	61.8680218	4.16870761	2.236505589	0.324020484
DTH	20.19739001***	13.03457404***	2.060156844	3.87E–55	2.13E–25	88.55111596	5.042324704	1.620899558	0.858717474
DTM	21.03642733***	12.40150291***	10.82968701	1.41E–22	1.20E–10	131.165459	6.060198856	2.508929565	0.85949863
PH	31.69286996***	50.74887557***	4.11582575	1.62E–22	6.27E–40	91.30265415	8.659621398	2.222005284	0.712059979
TC	559.1622137***	1083.247364***	4.180694636	9.63E–24	6.27E–99	139.8989856	37.6215953	1.461536474	0.673604441
SL	0.527534839***	1.097611556***	0.033674386	1.24E–20	2.51E–53	10.34260301	1.197277366	1.774271127	0.656963106
SPS	0.880420876***	2.372640279***	0.092466703	8.63E–15	1.99E–55	19.89783089	1.679582261	1.528223772	0.596301015
GPS	19.33766195***	92.84912788***	0.874477153	1.47E–07	1.91E–86	60.53566503	9.053811551	1.544766761	0.454178047
TGW	15.28647417***	36.2920517***	0.618107393	5.85E–18	5.93E–53	41.34283443	6.682933421	1.901654962	0.627039572
GY	24.9655253**	223.990012***	0.664962551	0.001571724	1.24E–101	59.41251338	11.55302658	1.372526279	0.308277985
GL	0.053958654***	0.002603464^ ns^	0.0930213	4.81E–26	0.569175735	6.752534212	0.358297714	4.516732906	0.938143218
GB	0.035610021***	0.056027068***	0.0070362	6.65E–12	1.24E–15	3.77548664	0.292966443	2.221754847	0.71453453
GSA	1.921519183***	2.940549873***	0.145026502	1.27E–27	1.55E–33	19.46282531	2.073984033	1.956670972	0.722052954

^a^*Phi2* PS II quantum yield, *NPQ* non-photochemical quenching, *RC* relative chlorophyll, *CTD* canopy temperature depression, *PS1* active PS I center, *LT* leaf thickness, *LA* leaf angle, *DTH* days to heading, *DTM* days to maturity, *PH* plant height, *TC* tiller count, *SL* spike length, *SPS* spikelets per spike, *GPS* grains per spike, *TGW* thousand-grain weight, *GY* grain yield, *GL* grain length, *GB* grain breadth, *GSA* grain surface area.

^b^Mean square values from the combined analysis of variance.

^ns^*p* ≥ *0.05*; *p ≤ *0.05*; ** *p* ≤ *0.01*; *** p ≤ *0.001.*

### Correlations among traits

**Figure 3:**
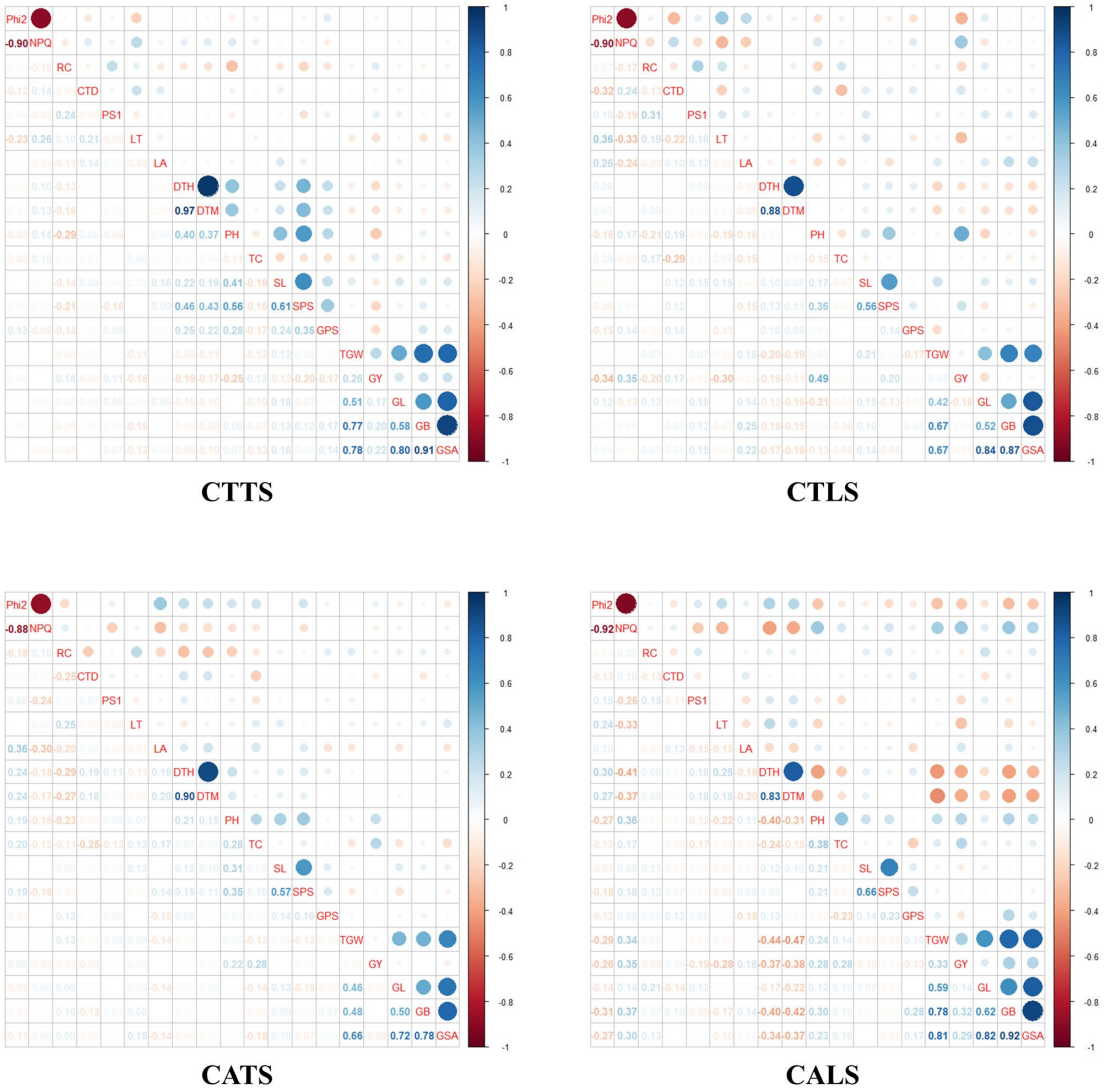
Correlation among the morpho-physiological traits under the four production environments (CTTS, conventional tillage timely sown; CTLS, conventional tillage late sown; CATS, conservation agriculture timely sown; CALS, conservation agriculture late sown). The pair plot shows Pearson’s correlation estimates among each trait pair below the diagonal, traits on the diagonal, and proportional correlation coefficients depicted as the colour and size of circles above the diagonal. *Phi2* PS II quantum yield, *NPQ* non-photochemical quenching, *RC* relative chlorophyll *CTD* canopy temperature depression *PS1* active PS I center *LT* leaf thickness *LA*, leaf angle, *DTH* days to heading, *DTM* days to maturity, PH plant height, *TC* tiller count, *SL* spike length, *SPS* spikelets per spike *GPS* grains per spike *TGW* thousand-grain weight, *GY* grain yield, *GL* grain length, *GB* grain breadth, *GSA* grain surface area.

Pairwise correlation based on Pearson’s coefficients was employed to examine the correlations between the morpho-physiological traits under each production environment separately to understand how the correlation patterns change under differing production conditions (Fig. 3). Significant positive correlations (*p* ≤ *0.05*) were identified between DTH and DTM (0.83 to 0.97) across the production environments. The negative correlation (−0.07) between NPQ and GY observed under timely-sown conditions turned positive (0.35) under late-sown environments, while a consistent negative relation between NPQ and Phi2 (− 0.88 to -0.90) across the environments was observed. PH showed a positive correlation with GY across the environments (0.22 to 0.49) except CTTS (− 0.25). Both DTH and DTM showed negative correlations with TGW, GY, GB, GL, and GSA across the environments. Significant positive correlations were found between Phi2 with DTH (0.24 to 0.30) and DTM (0.24 to 0.27) under CA environments but not in CT conditions. Interestingly, a positive correlation (0.10 to 0.24) was identified between NPQ and CTD with the highest value observed under CTLS (0.24). Furthermore, LT which showed a positive correlation with NPQ, had a negative correlation with Phi2 under timely-sown environments whereas the reverse relationship was observed under late-sown conditions. TC specifically showed a consistent positive correlation (0.28) with GY under both CA environments while no such relation was observed under CT environments. The correlation matrices for the 19 studied traits have been provided in the supplementary information (Supplementary Tables S4–S7) showing the significance of the correlations based on p-values from Pearson’s correlation test.

### Quality control, population structure and linkage disequilibrium

**Figure 4:**
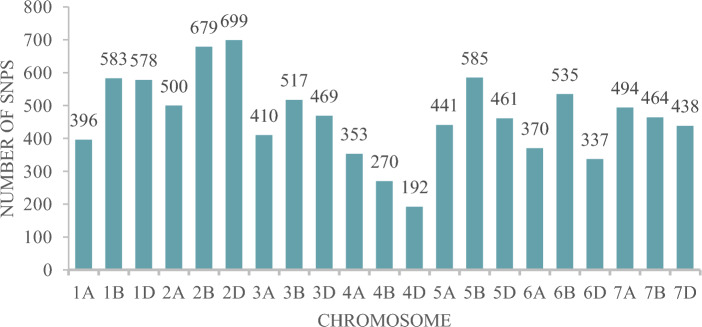
Bar graph depicting the chromosome-wise distribution of filtered SNPs across the genome.

**Figure 5:**
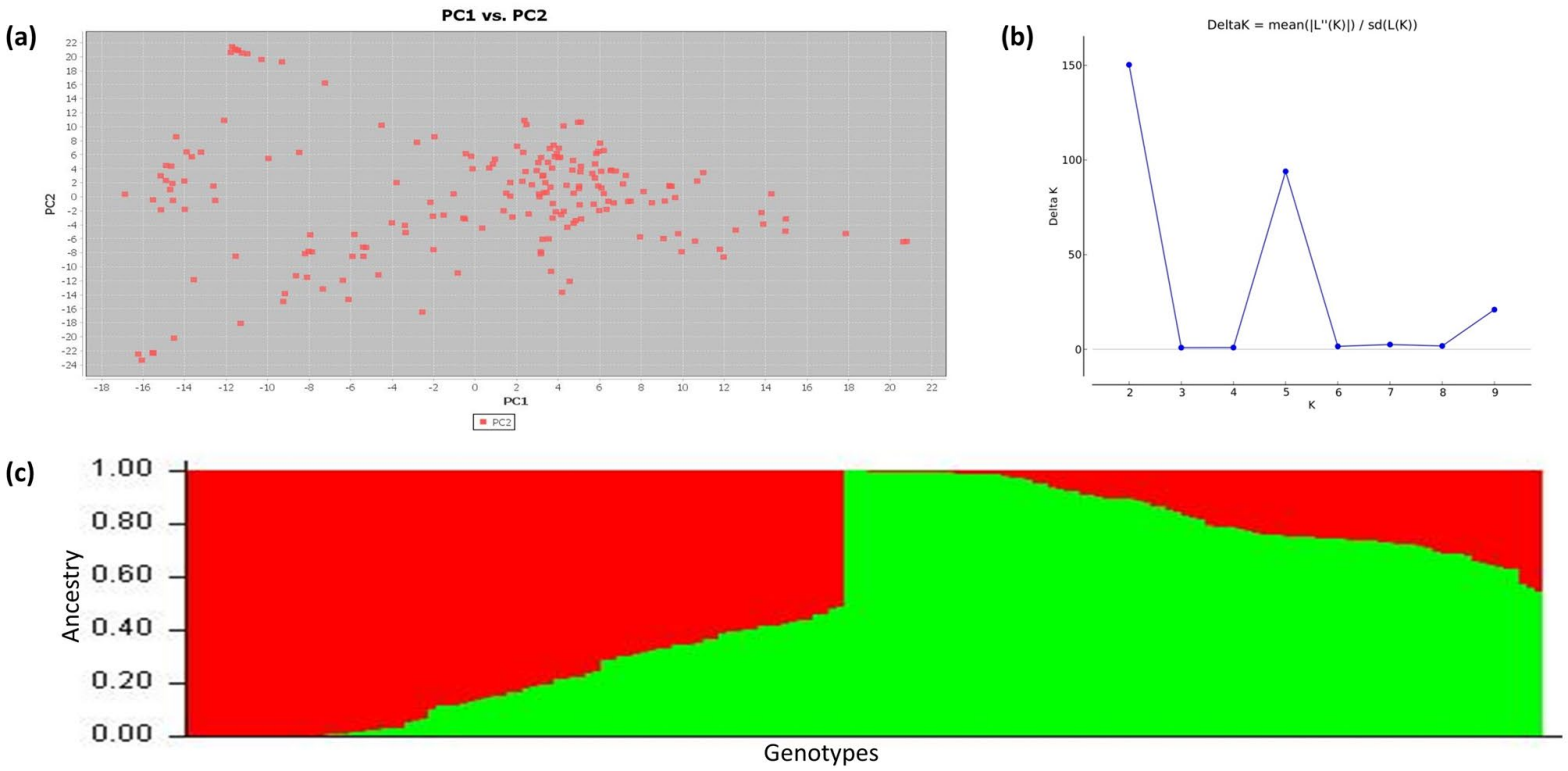
Population structure analysis. (**a**) PCA performed using the filtered genotypic data indicates the presence of two groups in the association panel; (**b**) Evanno plot depicting estimated ΔK values for a given K identified max ΔK value at K = 2; (**c**) Barplot of the population structure showing the presence of two sub-populations.

**Figure 6:**
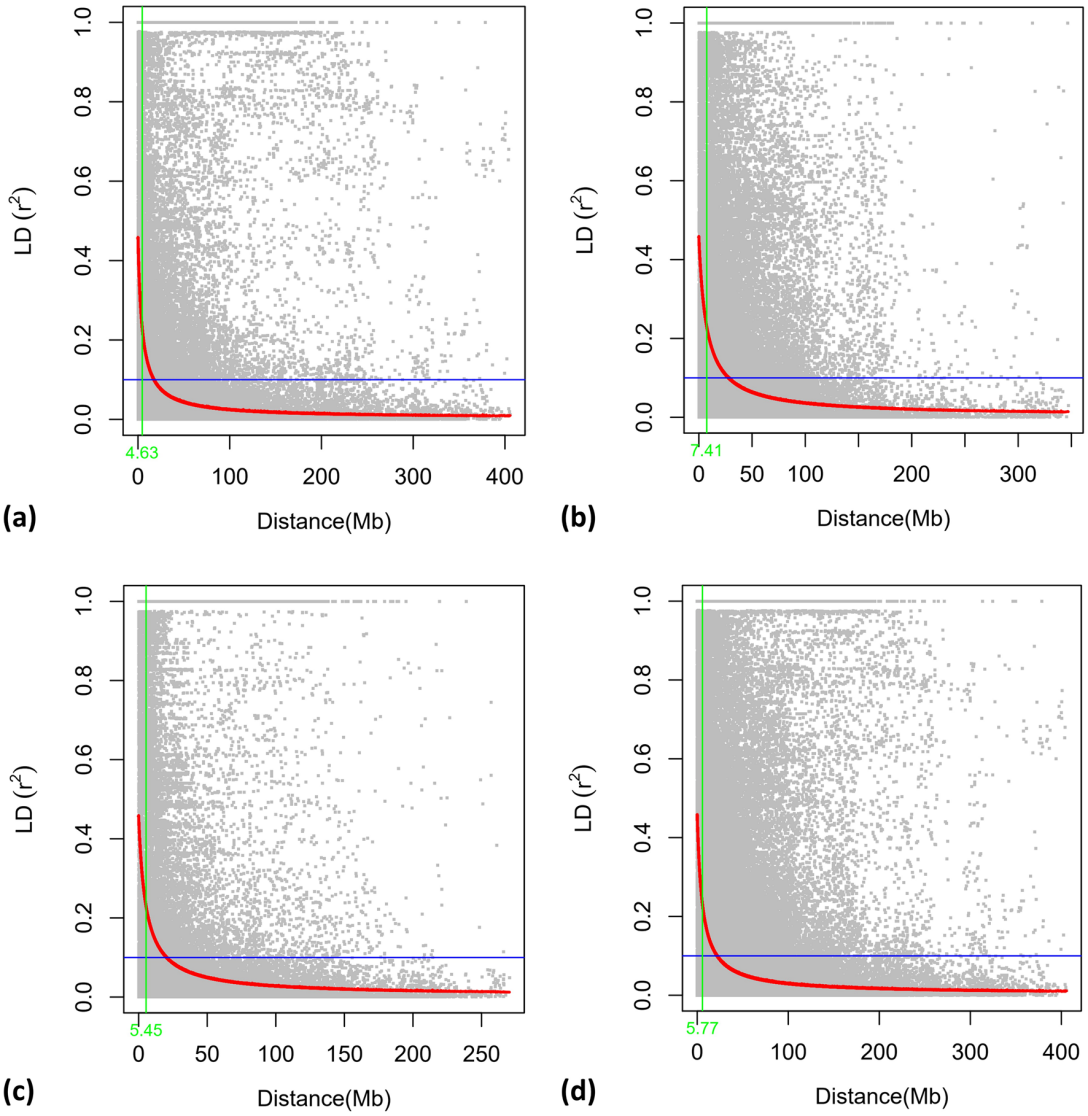
LD decay plots of (**a**) A genome; (**b**) B genome; (**c**) D subgenome; and (**d**) whole genome. The LD decay was the fastest in subgenome A.

The initial genotypic data contained 31,433 SNPs which were filtered to obtain a subset of 9,771 highly informative SNPs that had MAF > 0.05, heterozygosity < 0.15 and missing data < 0.20. The highest number of SNPs were found in the B genome (3633; 37.18%) followed by the D (3174; 32.48%) and A (2964; 30.33%) genomes. Chromosome 2D had the highest number of SNPs (699), while chromosome 4D had the lowest (192) as depicted in Fig. 4. Our analysis using Principal Component Analysis (PCA) revealed the presence of two distinct sub-populations within our data set. This separation is visualized by the first two principal components(Fig. 5a). The entire population was segregated into two sub-populations, as the ΔK value was maximized when K was set to two. (Fig. 5b). Also, using Evanno’s method in STRUCTURE Harvester, the optimal number of clusters (K) was determined to be two (Fig. 5c), which was consistent across both the STRUCTURE analysis and the graphing of ΔK values. R^2^ values for SNP pairs were utilised to assess LD decay by plotting against genetic distances. LOESS regression characterized LD decay, with the curve depicted in red on LD decay plots. A threshold was identified at r^2^ = 0.10, based on the fitted model. LD decay was fastest in sub-genome A, followed by D and B. Decay occurred at 4.63 Mb (A), 5.45 Mb (D), 7.41 Mb (B), and 5.77 Mb (genome-wide) as depicted in Fig. 6.

### Genome-wide association studies and gene annotation

**Figure 7:**
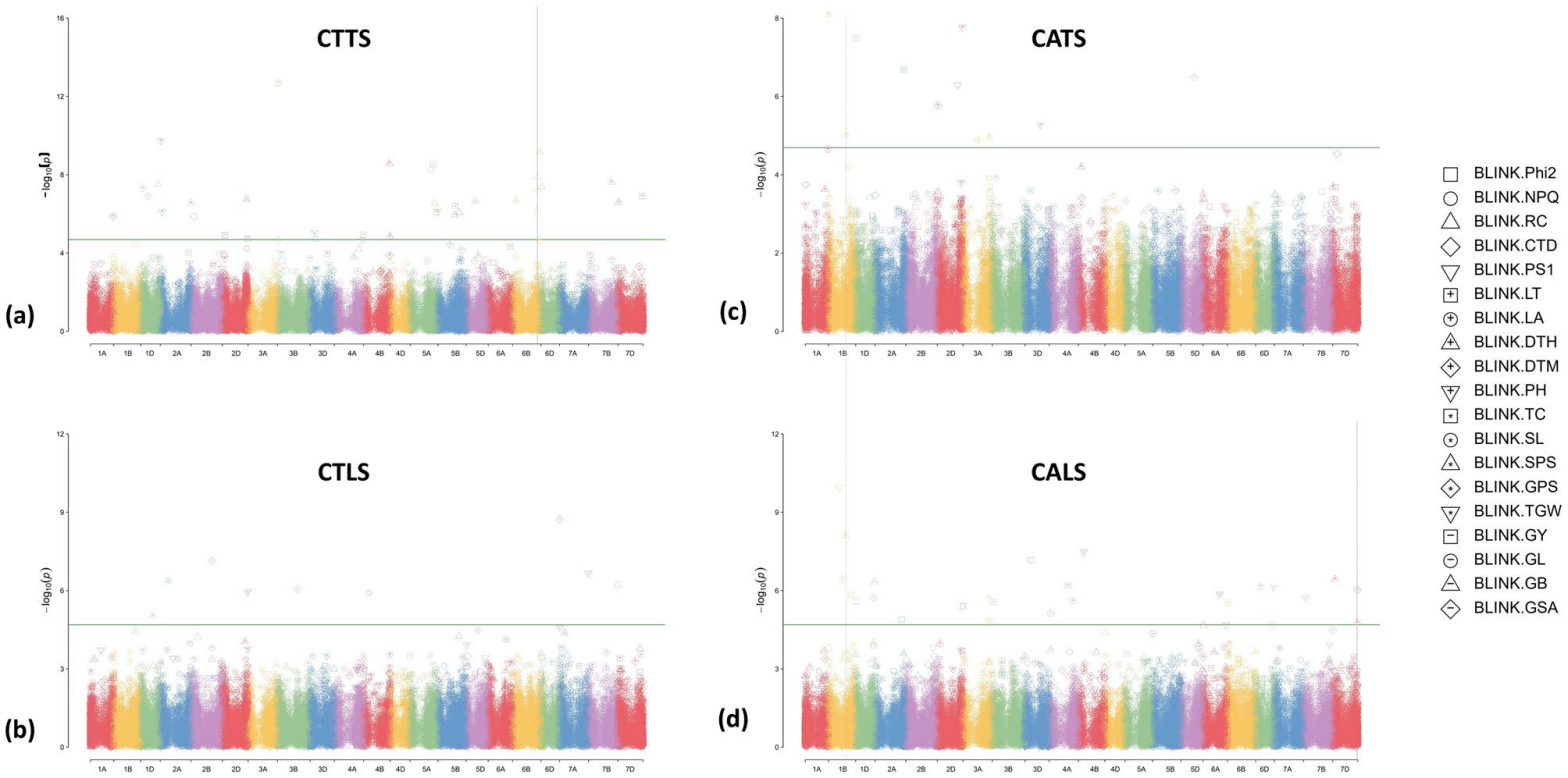
Manhattan plots showing identified MTAs through BLINK model for the morpho-physiological traits under (**a**) CTTS; (**b**) CTLS; (**c**) CATS; and (**d**) CALS. *Phi2* PS II quantum yield, *NPQ* non-photochemical quenching, *RC* relative chlorophyll, *CTD* canopy temperature depression, *PS1* active PS I center, *LT* leaf thickness, *LA* leaf angle, *DTH* days to heading, *DTM* days to maturity, *PH* plant height, *TC* tiller count, *SL* spike length, *SPS* spikelets per spike, *GPS* grains per spike, *TGW* thousand-grain weight, *GY* grain yield, *GL* grain length, *GB* grain breadth, *GSA* grain surface area.

To detect significant MTAs within each production environment using both phenotypic and genotypic data, the GAPIT3 package was employed in R Studio using the BLINK model. GWAS was performed for individual traits under each environment to uncover the presence of MTA*Environment interaction as many MTAs found in one environment could not be observed under other environments. To identify significant MTAs, a Bonferroni threshold was set at a -log_10_(p) value of 5.0. A total of 80 MTAs were identified through GWAS across all environments for all the traits, and their chromosome-wise and trait-wise distributions are depicted in Supplementary Figs. S1, S2, respectively. The highest number of significant MTAs were identified in the D subgenome (30) followed by the B subgenome (28) and A subgenome (22). The maximum number of significant MTAs were detected for DTH (11) followed by TGW (8), while LA (1) had the lowest. Upon a detailed investigation, it was observed that the A subgenome had the highest number of identified MTAs for LT (2), PH (3) and TC (2). In contrast, the B subgenome had the highest number of MTAs for DTH (8) and RC (3) while the D subgenome harboured the most MTAs for Phi2 (3), DTM (3), GB (2), GSA (3), GY (3) and SPS (3). In addition, Manhattan plots and QQ plots were created using the R package “GAPIT3” to detect significant MTAs by plotting SNPs that had a -log_10_(p) value of more than 5.0. The Manhattan plots demonstrating the identification of significant QTNs discovered by the BLINK model approach are depicted in Fig. 7.

The highest number of MTAs were identified in the CTTS (35) production environment followed by CALS (25) and CATS (11) while the lowest numbers were identified in CTLS (9). The Manhattan plots and QQ plots for the morpho-physiological traits in the present study under each production environment are shown in Supplementary Figs. S3–S21. Out of the total 80 significant MTAs initially identified, further analysis focused on the 48 most highly significant SNPs as shown in Table 2. Notably, **SNP AX95138506**, situated on Chromosome 2B, played a pivotal role by explaining 40.2% of the phenotypic variance for CTD under the CTTS condition. Similarly, **SNP AX94525104**, located on Chromosome 3A, accounted for a noteworthy 31.3% of the variance for GL under CALS. In addition to these, 9 other SNPs were identified, each contributing more than 20% to the phenotypic variance in various morpho-physiological traits. Interestingly, **SNP AX94803899**, located on chromosome 3B had the highest -log_10_(p) at 12.68 explaining a phenotypic variation of 12.98% for GY. Another **SNP AX95219528** on chromosome 1B was observed to be associated with DTH (PVE 11.88–21.42%) and was found to be consistent under both CATS and CALS environments. The remaining 36 SNPs, subjected to further gene annotation through *in-silico* analysis, elucidated phenotypic variances ranging from 2.15 to 19.45% for the different traits under study. Furthermore, it was observed that MTAs for Phi2 and NPQ were identified under CA and CT environments, respectively.

**Table 2:** List of selected Marker Trait Associations (MTAs) identified for morpho-physiological traits under contrasting production regimes through GWAS.

Trait	ENV	SNP	QTN	Chromosome	Position	P value	MAF	PVE (%)	− log_10_[P]
NPQ	CTTS	AX94477203	*QCTTS.iari-3DS_NPQ*	3D	137485826	1.92E–05	0.2500	6.75	4.717
NPQ	CTTS	AX94683305	*QCTTS.iari-5AL_NPQ*	5A	524721500	5.12E–09	0.0824	16.99	8.291
RC	CTTS	AX95247332	*QCTTS.iari-1DL_RC*	1D	439868237	3.12E–08	0.1622	6.89	7.506
RC	CTTS	AX94796135	*QCTTS.iari-5AL_RC*	5A	624213725	2.88E–07	0.0771	4.22	6.541
RC	CTTS	AX94449793	*QCTTS.iari-5BL_RC*	5B	547583889	8.52E–07	0.1170	3.38	6.070
RC	CTTS	AX94416085	*QCTTS.iari-6BL_RC*	6B	634048194	1.29E–08	0.0559	8.49	7.889
RC	CTTS	AX95249924	*QCTTS.iari-6DS_RC*	6D	16882808	4.17E–08	0.0638	8.27	7.380
CTD	CTTS	AX95138506	*QCTTS.iari-2BS_CTD*	2B	72815939	1.41E–06	0.0824	40.22	5.851
DTH	CTTS	AX95202058	*QCTTS.iari-2BS_DTH*	2B	7428753	2.76E–07	0.2872	2.28	6.559
DTH	CTTS	AX94986002	*QCTTS.iari-4BLa_DTH*	4B	657990217	2.62E–09	0.3298	3.57	8.582
DTH	CTTS	AX94486277	*QCTTS.iari-4BLb_DTH*	4B	673203278	1.38E–05	0.2101	2.15	4.860
DTH	CTTS	AX94430452	*QCTTS.iari-6BS_DTH*	6B	92697905	2.12E–07	0.0585	10.01	6.674
DTH	CTTS	AX94950339	*QCTTS.iari-6BL_DTH*	6B	678739547	6.50E–10	0.2553	7.24	9.187
DTH	CTTS	AX94522036	*QCTTS.iari-7BL_DTH*	7B	581399895	2.36E–08	0.1064	6.20	7.627
DTM	CTTS	AX94521760	*QCTTS.iari-1AL_DTM*	1A	584469113	1.32E–06	0.2314	7.89	5.879
DTM	CTTS	AX94950280	*QCTTS.iari-1DS_DTM*	1D	48401200	4.75E–08	0.4069	6.70	7.323
DTM	CTTS	AX94544614	*QCTTS.iari-1DL_DTM*	1D	182039280	1.21E–07	0.1277	7.85	6.917
PH	CTTS	AX95153371	*QCTTS.iari-2AS_PH*	2A	16672400	1.82E–10	0.2447	3.95	9.740
PH	CTTS	AX94681462	*QCTTS.iari-5AL_PH*	5A	702753259	7.53E–07	0.0532	11.77	6.123
TC	CTTS	AX95181791	*QCTTS.iari-5AL_TC*	5A	584672964	3.02E–09	0.0878	29.47	8.520
TC	CTTS	AX95110699	*QCTTS.iari-5BL_TC*	5B	435158879	4.03E–07	0.1144	9.84	6.395
SL, SPS	CTTS	AX94707304	*QCTTS.iari-6BL_SL,SPS*	6B	631966790	6.38E–07	0.0824	26.90	6.195
SPS	CTTS	AX94669346	*QCTTS.iari-6BL_SPS*	2D	609234499	1.72E–07	0.0745	11.10	6.764
TGW	CTTS	AX94879209	*QCTTS.iari-3DS_TGW*	3D	112759910	9.31E–06	0.1543	19.00	5.031
GY	CTTS	AX94803899	*QCTTS.iari-3BS_GY*	3B	26650236	2.05E–13	0.0771	12.98	12.688
GY	CTTS	AX94514123	*QCTTS.iari-5AL_GY*	5A	666680993	8.36E–07	0.0878	3.28	6.078
GY	CTTS	AX94819762	*QCTTS.iari-7DL_GY*	7D	616257059	1.24E–07	0.0984	3.51	6.907
GB	CTTS	AX95194941	*QCTTS.iari-5BL_GB*	5B	421275897	1.13E–06	0.2074	6.56	5.947
NPQ	CTLS	AX94730536	*QCTLS.iari-4BS_NPQ*	4B	134118354	1.21E–06	0.1489	30.90	5.917
DTH	CTLS	AX94476240	*QCTLS.iari-1DL_DTH*	1D	305590589	9.03E–06	0.1543	10.52	5.044
SL	CTLS	AX94650173	*QCTLS.iari-2AS_SL*	2A	197557797	4.11E–07	0.1835	21.89	6.386
GPS	CTLS	AX94882016	*QCTLS.iari-3BL_GPS*	3B	488187764	8.67E–07	0.2101	17.17	6.062
PS1	CATS	AX95178106	*QCATS.iari-2DL_PS1*	2D	519133490	5.06E–07	0.1090	23.05	6.296
LA	CATS	AX94454667	*QCATS.iari-1BS_LA*	1B	1942165	7.97E–09	0.1463	24.02	8.099
DTH	CATS, CALS	AX95219528	*QCATS.iari-1BL_DTH*	1B	436292401	9.36E–06	0.0691	11.88–21.42	5.029
DTH	CATS	AX94476007	*QCATS.iari-3AL_DTH*	3A	663297662	1.10E–05	0.0904	19.45	4.959
DTM	CATS	AX94937481	*QCATS.iari-2DS_DTM*	2D	12872093	1.70E–06	0.0824	11.98	5.770
DTM	CATS	AX95168777	*QCATS.iari-3AL_DTM*	3A	401710412	1.28E–05	0.0718	18.72	4.893
PH	CATS	AX95242866	*QCATS.iari-2DL_PH*	2D	633755644	1.72E–08	0.1596	15.82	7.764
TC	CATS	AX94773494	*QCATS.iari-1DS_TC*	1D	7906507	3.22E–08	0.2846	10.72	7.492
Phi2	CALS	AX94651261	*QCALS.iari-1BL_Phi2*	1B	571707456	1.41E–06	0.1223	9.43	5.851
Phi2	CALS	AX95228767	*QCALS.iari-2DL_Phi2*	2D	650956289	4.00E–06	0.1356	3.51	5.398
LT	CALS	AX94985892	*QCALS.iari-4AL_LT*	4A	598714500	2.44E–06	0.0559	19.45	5.613
DTM	CALS	AX94658713	*QCALS.iari-6BS_DTM*	6B	17401946	2.96E–06	0.0585	22.87	5.529
TGW	CALS	AX94543129	*QCALS.iari-6DL_TGW*	6D	452632475	7.73E–07	0.0691	6.11	6.112
GL	CALS	AX94525104	*QCALS.iari-3AL_GL*	3A	652820340	1.88E–06	0.0798	31.33	5.726
GB, GSA	CALS	AX94857819	*QCALS.iari-7DL_GB,GSA*	7D	619993835	1.69E–05	0.0851	27.66	4.772
GSA	CALS	AX95025855	*QCALS.iari-1BL_GSA*	1B	359199100	3.63E–07	0.0878	6.27	6.440

*Phi2* PS II quantum yield, *NPQ* non-photochemical quenching, *RC* relative chlorophyll, *CTD* canopy temperature depression, *PS1* active PS I center, *LT* leaf thickness, *LA* leaf angle, *DTH* days to heading, *DTM* days to maturity, *PH* plant height, *TC* tiller count, *SL* spike length, *SPS* spikelets per spike, *GPS* grains per spike, *TGW* thousand-grain weight, *GY* grain yield, *GL* grain length, *GB* grain breadth, *GSA* grain surface area, *CTTS* Conventional tillage timely sown, *CATS* Conservation agriculture timely sown, *CTLS* Conventional tillage late sown, *CALS* conservation agriculture late sown.

Out of the total 80 SNP markers associated with 19 morpho-physiological traits, 48 SNPs were found to be located within and contiguous to genes, and they are listed in Table 2. The detailed investigation focused solely on high-confidence protein-coding genes that play a role in the expression and development of the associated traits. Genes identified through an *in-silico* analysis were mostly transcription factors that have been reported to overexpress in stress conditions, peroxidases, kinases, pentatricopeptide repeat proteins, splicing factors, proteins involved in photophosphorylation and electron transport chain, various kinds of domain-containing proteins and sugar transporters. Details of the candidate genes identified through gene annotation are provided in Table 3.

**Table 3:** Gene annotation through *in-silico* analysis to identify candidate genes.

Trait	QTN	Candidate gene ID	Chromosome: location (bp)	Gene annotation
Phi2	*QCALS.iari-1BL_Phi2*	TraesCS1B02G343300	1B:571444691–571445480	Photosystem II reaction center W protein, chloroplastic
	*QCALS.iari-2DL_Phi2*	TraesCS2D02G599200	2D:650684790–650686459	Cytochrome P450 89A9
NPQ	*QCTTS.iari-5AL_NPQ*	TraesCS5A02G314800	5A:525115605–525117944	UDP-glucose 4-epimerase 3
		TraesCS5A02G314700	5A:525077777–525079517	Heat stress transcription factor B-2c
		TraesCS5A02G314600	5A:525025721–525026755	Ethylene-responsive transcription factor ERF071
	*QCTLS.iari-4BS_NPQ*	TraesCS4B02G116200	4B:133851917–133854945	UDP-glucose 6-dehydrogenase 4
	*QCTTS.iari-3DS_NPQ*	TraesCS3D02G165300	3D:137002179–137006358	25.3 kDa vesicle transport protein
RC	*QCTTS.iari-5AL_RC*	TraesCS5A02G444500	5A:624706560–624708402	UDP-glycosyltransferase 73E1
	*QCTTS.iari-5BL_RC*	TraesCS5B02G368900	5B:547652774–547657481	Chaperonin 60 subunit alpha 2, chloroplastic
	*QCTTS.iari-6BL_RC*	TraesCS6B02G363300	6B:634501638–634502222	Putative ripening-related protein 5
	*QCTTS.iari-1DL_RC*	TraesCS1D02G355200	1D:439878998–439883243	Hexokinase-2
		TraesCS1D02G354100	1D:439414111–439415360	Peroxidase 1
	*QCTTS.iari-6DS_RC*	TraesCS6D02G042400	6D:17309364–17310623	Putative wall-associated receptor kinase-like 16
		TraesCS6D02G042300	6D:17297853–17304530	Wall-associated receptor kinase 2
CTD	*QCTTS.iari-2BS_CTD*	TraesCS2B02G111000	2B:72811061–72817327	Probable glutamyl endopeptidase, chloroplastic
		TraesCS2B02G110800	2B:72608545–72619945	Lon protease homolog
PS1	*QCATS.iari-2DL_PS1*	TraesCS2D02G404800	2D:519132647–519135038	Aquaporin PIP1-2
		TraesCS2D02G404600	2D:518969013–518971345	Cytochrome P450 86A22
		TraesCS2D02G404500	2D:518810879–518814738	Thioredoxin-like 3–3
LT	*QCALS.iari-4AL_LT*	TraesCS4A02G192300	4A:472917832–472921560	Cyclin-dependent kinase B1-1
LA	*QCATS.iari-1BS_LA*	TraesCS1B02G004100	1B:2354630–2357674	Wall-associated receptor kinase 1
DTH	*QCATS.iari-3AL_DTH*	TraesCS3A02G422600	3A:663574417–663576507	Glycine-rich RNA-binding protein 4, mitochondrial
		TraesCS3A02G421400	3A:662931519–662933524	Transcription factor bHLH49
		TraesCS3A02G421900	3A:663258012–663265132	26S proteasome regulatory subunit 6A homolog A
	*QCTTS.iari-2BS_DTH*	TraesCS2B02G015500	2B:7428456–7443833	Histone-lysine N-methyltransferase ASHH2
	*QCTTS.iari-4BLa_DTH*	TraesCS4B02G374400	4B:658211040–658212993	GATA transcription factor 2
		TraesCS4B02G373700	4B:657991716–657993829	VAN3-binding protein
		TraesCS4B02G373600	4B:657989337–657991890	Formyltetrahydrofolate deformylase 2, mitochondrial
	*QCTTS.iari-4BLb_DTH*	TraesCS4B02G398900	4B:673112846–673115678	Serine / arginine-rich splicing factor RS31
	*QCTTS.iari-6BL_DTH*	TraesCS6B02G402700	6B:678744926–678745984	BTB / POZ and MATH domain-containing protein 1
	*QCTTS.iari-6BS_DTH*	TraesCS6B02G110200	6B:92523435–92524395	18.9 kDa heat shock protein
		TraesCS6B02G110400	6B:92697243–92698587	Probable xyloglucan endotransglucosylase/ hydrolase protein 30
		TraesCS6B02G110600	6B:92749833–92755866	Ribose-phosphate pyrophosphokinase 1, chloroplastic
	*QCTTS.iari-7BL_DTH*	TraesCS7B02G327700	7B:581892922–581894632	Probable xyloglucan endotransglucosylase/ hydrolase protein 23
		TraesCS7B02G327600	7B:581794516–581796383	Inositol polyphosphate multikinase IPK2
		TraesCS7B02G327400	7B:581372974–581376095	Xyloglucan endotransglucosylase/ hydrolase protein 22
	*QCATS.iari-1BL_DTH*	TraesCS1B02G246600	1B:436536923–436537890	Protein SRC2
	*QCTLS.iari-1DL_DTH*	TraesCS1D02G218700	1D:305586356–305590935	RNA-binding KH domain-containing protein PEPPER
DTM	*QCTTS.iari-1AL_DTM*	TraesCS1A02G433400	1A:584676043–584676930	Pentatricopeptide repeat-containing protein At2g15690, mitochondrial
	*QCATS.iari-3AL_DTM*	TraesCS3A02G219300	3A:402135204–402136682	Protein tesmin / TSO1-like CXC 4
	*QCALS.iari-6BS_DTM*	TraesCS6B02G028300	6B:16996335–16996970	MADS-box transcription factor PHERES 2
	*QCTTS.iari-1DL_DTM*	TraesCS1D02G066600	1D:48021551–48024273	Importin subunit beta-1
		TraesCS1D02G066400	1D:47934766–47936019	Polygalacturonase QRT2
	*QCTTS.iari-1DS_DTM*	TraesCS1D02G136400	1D:181626866–181639553	Serine / threonine-protein kinase EDR1
	*QCATS.iari-2DS_DTM*	TraesCS2D02G031400	2D:12619687–12623828	Transcription factor E2FB
		TraesCS2D02G030800	2D:12550664–12552270	7-deoxyloganetin glucosyltransferase
PH	*QCTTS.iari-2AS_PH*	TraesCS2A02G041300	2A:16719074–16727514	Fatty acid amide hydrolase
		TraesCS2A02G041500	2A:16745853–16746524	Ethylene-responsive transcription factor 1B
	*QCTTS.iari-5AL_PH*	TraesCS5A02G548300	5A:702459427–702460136	PLASMODESMATA CALLOSE-BINDING PROTEIN 3
		TraesCS5A02G548500	5A:702501770–702503426	Probable xyloglucan endotransglucosylase/ hydrolase protein 32
		TraesCS5A02G548700	5A:702631484–702632670	Heat stress transcription factor C-2a
		TraesCS5A02G549400	5A:703181022–703183341	UDP-glycosyltransferase 85A1
	*QCATS.iari-2DL_PH*	TraesCS2D02G560400	2D:633258611–633259105	SKP1-like protein 1A
TC	*QCTTS.iari-5AL_TC*	TraesCS5A02G386600	5A:584415361–584420062	YTH domain-containing protein ECT2
	*QCTTS.iari-5BL_TC*	TraesCS5B02G252600	5B:435597303–435598318	Probable E3 ubiquitin-protein ligase XERICO
		TraesCS5B02G252500	5B:435156029–435159077	Serine / threonine-protein kinase D6PKL2
	*QCATS.iari-1DS_TC*	TraesCS1D02G016800	1D:7583590–7587977	Wall-associated receptor kinase 2
SL	*QCTLS.iari-2AS_SL*	TraesCS2A02G212200	2A:197088104–197089901	Transcription factor UDT1
	*QCTTS.iari-6BL_SL,SPS*	TraesCS6B02G360300	6B:632182793–632188473	Soluble inorganic pyrophosphatase 6, chloroplastic
		TraesCS6B02G360500	6B:632304665–632305774	WAT1-related protein At2g39510
SPS	*QCTTS.iari-6BL_SPS*	TraesCS6B02G360300	6B:632182793–632188473	Soluble inorganic pyrophosphatase 6, chloroplastic
		TraesCS6B02G360500	6B:632304665–632305774	WAT1-related protein At2g39510
GPS	*QCTLS.iari-3BL_GPS*	TraesCS3B02G303600	3B:487855328–487857841	Aldehyde oxidase GLOX1
		TraesCS3B02G304300	3B:488542649–488550339	Protein MALE DISCOVERER 1
		TraesCS3B02G304200	3B:488535427–488537668	Rop guanine nucleotide exchange factor 11
TGW	*QCTTS.iari-3DS_TGW*	TraesCS3D02G148000	3D:112694083–112698708	Sodium/calcium exchanger NCL1
		TraesCS3D02G148200	3D:112960872–112963125	Basic leucine zipper 2
	*QCALS.iari-6DL_TGW*	TraesCS6D02G362000	6D:452735812–452739910	SAP-like protein BP-73
GY	*QCTTS.iari-5AL_GY*	TraesCS5A02G500700	5A:666682422–666683774	NAC domain-containing protein 22
	*QCTTS.iari-3BS_GY*	TraesCS3B02G052900	3B:26751728–26753832	Beta-1,2-xylosyltransferease XAX1
	*QCTTS.iari-7DL_GY*	TraesCS7D02G516500	7D:616035255–616041322	Protein PSK SIMULATOR 1
		TraesCS7D02G515100	7D:615774804–615776009	Transcription factor MYB44
GL	*QCALS.iari-3AL_GL*	TraesCS3A02G408200	3A:653303151–653306203	Bidirectional sugar transporter SWEET1a
		TraesCS3A02G408000	3A:653240579–653244186	Scarecrow-like protein 1
GB	*QCTTS.iari-5BL_GB*	TraesCS5B02G241900	5B:421645424–421646515	Oil body-associated protein 1A
	*QCALS.iari-7DL_GB,GSA*	TraesCS7D02G523600	7D:620116105–620117371	Bifunctional monodehydroascorbate reductase and carbonic anhydrase nectarin-3
GSA	*QCALS.iari-1BL_GSA*	TraesCS1B02G200300	1B:359198865–359209340	Tubulin-folding cofactor D

*Phi2* PS II quantum yield, *NPQ* non-photochemical quenching, *RC* relative chlorophyll, *CTD* canopy temperature depression, *PS1* active PS I center, *LT* leaf thickness, *LA* leaf angle, *DTH* days to heading, *DTM* days to maturity, *PH* plant height, *TC* tiller count, *SL* spike length, *SPS* spikelets per spike, *GPS* grains per spike, *TGW* thousand-grain weight, *GY* grain yield *GL* grain length, *GB* grain breadth, *GSA* grain surface area.

## Discussions

In the Indo-Gangetic plain zone, the prevalent cropping system is the rice–wheat rotation. A significant factor contributing to the low yield of wheat in this system is the delayed sowing of wheat, often caused by the late transplanting and harvesting of the preceding rice crop. To address this issue and establish rice–wheat as a sustainable system, the adoption of zero tillage techniques for wheat cultivation can be highly beneficial^48^. With delayed wheat sowing in India, the occurrence of high-temperature events during the grain-filling stage becomes more frequent. Conservation agriculture (CA) represents a valuable approach to combat the impacts of climate change on agriculture. By minimizing soil disturbance, promoting soil health, and reducing greenhouse gas emissions, CA practices contribute to climate change mitigation^49^. Simultaneously, CA’s ability to enhance water conservation, improve crop resilience, and diversify agricultural systems aids in adapting to the changing climate. Breeding wheat varieties optimized for Conservation Agriculture (CA) involves a multifaceted approach, targeting traits such as drought tolerance, disease resistance, improved root architecture, shorter growth cycles, stress tolerance, nutrient efficiency, and lodging resistance^19,50^. These traits align with CA principles by enhancing crop resilience and adaptability to changing environmental conditions, improving soil health, and promoting sustainable agricultural practices.

The advanced breeding lines were observed to have ample variation for the studied morpho-physiological traits in the present study within each production environment as well as across them which creates a possibility to target these materials in various breeding programmes (Table 1). The variability for the studied traits among the genotypes was higher in CT environments than in CA environments signifying the role of management practices on crop performance. Among the physiological traits, the heritability estimates were higher for RC (0.71) and LT (0.62) while moderate heritability was observed for NPQ (0.46) and LA (0.32), and low heritability in the case of Phi2 (0.26) and CTD (0.24). Although GY (0.31) had a lower heritability estimate, moderate (0.45 for GPS and 0.60 for SPS) to high heritability (0.63 for TGW, 0.66 for SL, 0.67 for TC, 0.71 for PH, and 0.85 for both DTH and DTM) were observed among the agro-morphological traits. The seed geometric parameters had high heritability estimates (0.71 for GB, 0.72 for GSA and 0.94 for GL). Similar heritability estimates for physiological and agro-morphological traits were reported in previous studies^51–53^. The moderate to high heritability estimates for most of the studied traits across the production environments suggest that these traits can be utilised to understand the genetic control of these traits upon grain yield and their utilisation in improving grain yield in wheat.

Complex correlation patterns were identified for the studied traits due to the influence of change in production regimes. Among the two chlorophyll fluorescence parameters, Phi2 was positively correlated and NPQ was negatively correlated with GY under timely-sown (TS) conditions whereas the relationships changed under late-sown (LS) conditions as the correlation between NPQ and Phi2 with GY turned positive and negative, respectively. This signifies that plants grown under LS conditions are exposed to more light stress due to the coincidence of high temperature and light intensity with the reproductive growth stages of the crop. Thus, such genotypes which can maintain optimum Phi2 against NPQ, as plants use photoprotective mechanisms to reduce photoinhibition of both PS II and PS I complexes, are supposedly more tolerant to such high light and heat stress^54–56^. The consistent negative correlations (− 0.11 to − 0.37) of GY with DTH and DTM across the four production environments except for CATS wherein no significant negative correlation of GY with these phenological traits was observed indicates that plants grown under CATS get an opportunity to accumulate more photosynthetic reserves during the vegetative stage as the stress factors are less prominent in this ideal environment^50,57^. Additionally, in CA, there is a shift from a negative correlation (− 0.10 to − 0.17) between RC and NPQ in conventional tillage (CT) to a positive one (0.07 to 0.10), indicating that CA’s reduced soil disturbance and improved structure offer resilience against stress, unlike the variable responses in CTLS conditions. Under TS conditions, PH was negatively correlated (−0.25)with GY in CT while in CA, the relationship was observed to be positive (0.20). PH and GY are negatively correlated traits^58,59^ as taller plants are more prone to lodge due to heavy wind during the maturity stages but the positive relationship in CA represents an opportunity to target increased PH for improved GY as plants grown under CA have better anchorage due to less soil disturbance and better soil structure^18^. Kernel traits such as TGW, GL, GB and GSA showed moderate to high positive correlations (ranging from 0.14 to 0.92) which corroborate with previous studies^60,61^. Furthermore, CTD showed a significant positive correlation with GY (0.17) only under CTLS out of the four production environments as also reported in previous studies^62–64^. This suggests that plants sown under TS conditions produce optimum GY by completing most of the grain filling before heat stress coincides when compared to crops grown under LS conditions wherein reproductive growth stages coincide with high temperature which hampers GY. Interestingly, under LS conditions, only CT showed a positive correlation between CTD and GY whereas no such relationship was observed in CA which indicates plants grown in CA are less exposed to high-temperature stress as lower soil temperatures are offered by CA compared to CT^19^.

The adoption of marker-assisted breeding approaches holds the potential to empower wheat breeders in selecting promising genotypes that demonstrate improved performance under CA practices. DNA-based markers, including abundant SNPs present within the wheat genome, can be harnessed for this purpose, and be identified using high-throughput techniques such as GWAS. GWAS involves the integration of phenotypic data related to the traits of interest with genotypic data generated through sequencing for the identification of significant MTAs based on a Bonferroni threshold^65^, aiding breeders in their selection efforts. Contemplating the considerations mentioned earlier, the primary emphasis of this study was placed on identifying MTAs for the 19 morpho-physiological traits under contrasting production regimes through GWAS. The effectiveness of GWAS in uncovering significant MTAs hinges significantly on the marker distribution within the genotypic dataset. GWAS is more likely to identify MTAs with greater precision when markers are evenly distributed across the genome. Conversely, the presence of rare alleles diminishes GWAS’s resolution power. Additionally, the existence of population structure, if not properly addressed, can lead to unrelated associations and false positive results. To account for relatedness among the genotypes used in association mapping, kinship analysis is employed. Among all these factors, LD plays a crucial role in MTA identification^66^. High LD requires fewer markers, while low LD necessitates a larger number of markers for GWAS. Ensuring the quality of both phenotypic and marker genotypic data is paramount in the association mapping process. In the present study, we observed rapid Linkage Disequilibrium (LD) decay in the A subgenome (4.63 Mb), followed by the D subgenome (5.45 Mb) and the B subgenome (7.41 Mb), with a whole-genome LD decay rate of 5.77 Mb. A similar trend for subgenome-wise distribution of markers and rate of LD decay has been reported by Krishnappa et al.^67^. 48 MTAs out of the total 80 MTAs were selected for detailed study after gene annotation that identified putative candidate genes that are related to the development of the target traits.

The ϕII quantum yield (Phi2) is essentially the percentage of incoming light (excited electrons) that goes into the Photosystem II (Photophosphorylation), where the light energy is converted into chemical energy. Thus, increasing the proportion of Phi2 can improve the photosynthetic efficiency thereby increasing the grain yield. Two known genes “**Photosystem II reaction center W protein, chloroplastic**”^68,69^ on Chr1B and “**Cytochrome P450 89A9**”^70–72^ on Chr2D were found to be linked to markers **AX94651261** and **AX95228767**, respectively for Phi2 that are known to play pivotal roles in photophosphorylation and can be targeted to improve the photosynthetic efficiency. The identification of these significant MTAs under CALS emphasizes their role in the enhanced physiological response of genotypes towards adaptation to CA with improved stress tolerance. Due to its ideal environment, the CA offers a chance for crops to reach their maximum potential. Better and longer-lasting soil moisture availability and temperature modulation have produced this environment^73^. In a late-sown scenario with CT conditions, the crop experiences stress due to the rapid depletion of soil moisture. On the other hand, crops may withstand stress for a longer period under CA. While Phi2 relates to the proportion of absorbed light that goes into photochemical reactions, ϕNPQ (NPQ) is the proportion of the excitement energy of the PSII complex that is dissipated as heat to protect the plant against photooxidative damage and is referred to as non-photochemical quenching. Other than these two known regulated processes, the excess absorbed energy is expressed as ϕNO (non-regulated NPQ processes) and since these three processes of energy dissipation are proportions of the absorbed light energy, their sum is equal to 1. As NPQ is considered a photoprotective mechanism of plants, several stress-responsive genes are assumed to be involved in the process. We identified three MTAs for NPQ and through gene annotation, it was found that SNP **AX94730536** (PVE 30.90%) on Chr4B was linked to “**UDP-glucose 6-dehydrogenase 4**” gene while “**25.3 kDa vesicle transport protein**” gene was linked to SNP **AX94477203** (6.75% PVE) on Chr3D. In addition, SNP **AX94683305** (16.99% PVE) was found to be associated with three genes viz., **TraesCS5A02G314800**, **TraesCS5A02G314700** and **TraesCS5A02G314600** for “**UDP-glucose 4-epimerase**”, “**Heat stress transcription factor B-2c**” and “**Ethylene-responsive transcription factor ERF071**”, respectively. All these proteins have been reported to be differentially expressed under stress conditions^74–76^ and induce tolerance^77–81^. According to our data, NPQ under CT (0.54) was higher for both TS and LS than it was for CA (0.49). Due to the fast loss of soil water and the rising temperatures of the soil and air, the impact of stress is greater in CTLS conditions than in CALS. The fact that no QTNs for NPQ were found in the study under CA conditions indicates that CA is a superior environment when stress levels are high. Under stress conditions, plants initiate the accumulation of reactive oxygen species (ROS) within chloroplasts as a result of excess excitation energy, and the primary sites for the generation of these ROS are the reaction centers of Photosystem I (PS I) and Photosystem II (PS II) in the thylakoids^82^. Due to such ROS production, photo-inhibitive damage occurs in the PS I centers thereby reducing the number of PS I active reaction centers. The PS1 Active Centers (PS1) was found to be associated with SNP **AX95178106** (23.05% PVE) on Chr2D which is linked with three genes viz., **TraesCS2D02G404800**, **TraesCS2D02G404600** and **TraesCS2D02G404500** that codes for “**Aquaporin PIP1-2**”, “**Cytochrome P450 86A22**” and “**Thioredoxin-like 3-3**”, respectively. All three proteins are related to Photosystem I activity, as supported by evidence presented by Kromdijk et al.^83^; Zhang et al.^84^ and Zhu et al.^85^. Among the four production environments, CATS had an enhanced PS I activity (mean value of 2.45) as evidenced by the identification of all QTNs for PS1 in this environment, resulting in improved photophosphorylation.

Since, enhanced photosynthetic efficiency is a function of increased amount of chlorophyll content both under stress and non-stress conditions, targeting relative chlorophyll content has been a regular practice of breeders to select genotypes as a proxy trait for improved grain yield^86,87^. For relative chlorophyll content (RC), two genes **TraesCS1D02G355200** and **TraesCS1D02G354100** associated with SNP **AX95247332** (6.89% PVE) on Chr1D that translates into “**Hexokinases-2**” and “**Peroxidase 1**”, respectively have been reported to catalyze chlorophyll destruction leading to leaf senescence^88–90^. Also, SNP **AX94449793** (3.38% PVE) on Chr5B associated with **TraesCS5B02G368900** that translates into “**Chloroplastic Chaperon 60 subunit α-2**” has been reported to play an important role in protein transport under stress conditions^91,92^. Another SNP **AX95249924** on Chr6D was found to be present in close proximation with genes **TraesCS6D02G042400** and **TraesCS6D02G042300** that codes for “**Putative wall-associated receptor kinase-like 16**” and “**Wall-associated receptor kinase 2**” have been reported to negatively regulate leaf senescence and help plants maintain higher relative chlorophyll content even under stress conditions. Similar, findings were presented by Li et al.^93^; Riou et al.^94^; Zhao et al.^95^. The expression of these genes in the CT environment makes it more evident that these QTNs can be targeted to maintain relative chlorophyll contents under stress conditions. In contrast, no QTNs could be identified for RC under CA environments implying that plants thrive without the necessity for differential gene expression to uphold optimal chlorophyll content. This suggests that under CA conditions, where plant stress is minimized, maintaining ideal chlorophyll levels does not heavily depend on significant changes in gene expression. A “**Cyclin-dependent kinase B1-1**” protein coding gene **TraesCS4A02G192300** was found to be associated with SNP **AX94985892** (19.45% PVE) for leaf thickness (LT). The “**Cyclin-dependent kinase B1-1**” has been reported to promote Anaphase and is involved in increased growth rate and organ size^96^.

Crop phenology is highly influenced by the agrometeorological parameters and the production environment in which they are grown. Early heading, late maturity and increased plant height can be considered important non-grain parameters to select genotypes for improved grain yield under both timely and late sown conditions^97^. The highest number (8) of MTAs were found for days to heading (DTH). Notably, SNP **AX94476007** (19.45% PVE) on Chr3A was found to be linked to three genes viz., **TraesCS3A02G421900**, **TraesCS3A02G421400** and **TraesCS3A02G422600** for “**26S proteasome regulatory subunit 6A homolog A**”, “**Transcription factor bHLH49**” and “**Mitochondrial Glycine-rich RNA-binding protein 4**”, respectively. The role played by these genes in regulating the flowering time has been reported by Liu et al.^98^; Ahmad et al.^99^; Li et al.^100^; Alptekin et al.^101^; Ma et al.^102^. As described earlier in this section, CATS (97.71 and 150.47) was found to be the ideal production environment that had early heading and late maturity as compared to CTTS (97.96 and 145.64). Another SNP **AX94950339** (7.24% PVE) on Chr 6B was linked to **TraesCS6B02G402700** translates for “**BTB/POZ and MATH domain-containing protein 1**” which plays a role in vernalisation and regulates heading time under stress conditions as reported by Byrne et al.^103^ and Strejčková et al.^104^. Notably, SNP **AX95219528** (11.88–21.42% PVE) linked with **TraesCS1B02G246600** on Chr1B translates “**Protein SRC2**” was found in both CATS and CALS production environments, which has been reported to be upregulated during vernalisation^105^. 6 MTAs were found to be associated with days to maturity (DTM). Remarkably, SNP **AX94521760** (7.89% PVE) linked with **TraesCS1A02G433400** on Chr1A that translates into “**Mitochondrial Pentatricopeptide repeat-containing protein At2g15690**” which is reported to play a role in controlling the maturity time^106,107^. A “**MADS-box transcription factor PHERES 2**” coding gene **TraesCS6B02G028300** was found to be linked to SNP **AX94658713** (22.87%PVE) on Chr6B. The MADS-box genes belong to a well-known protein family that regulates floral transition, floral patterning, normal growth and development of carpels and fruits, and embryo and seed growth^108–110^. The identification of “**MADS-box transcription factor PHERES 2**” (upregulated under stress condition) associated SNP under CALS indicates its role in providing extended duration for grain filling^111^. SNP **AX94544614** (7.85% PVE) on Chr1D was found to be linked with genes **TraesCS1D02G066400** coding for “**Polygalacturonase QRT2**” regulating grain maturity^112–114^. For plant height (PH), SNP **AX95153371** (3.95% PVE) on Chr2A was linked with two genes **TraesCS2A02G041300** and **TraesCS2A02G041500** which translates for “**Fatty acid amide hydrolase**” and “**Ethylene-responsive transcription factor 1B**”, respectively both of which have been reported to play key roles in regulating plant growth and height^115–117^. Two SNPs viz., **AX95181791** (29.47% PVE) on Chr5A and **AX95110699** (10.72% PVE) on Chr1D linked with genes **TraesCS5A02G386600** and **TraesCS5B02G252500** which code for proteins “**YTH domain-containing protein ECT2**” and “**Serine / threonine-protein kinase D6PKL2**” were found to be associated with number of productive tillers (TC). *Arabidopsis* ECT2/3/4 proteins are reported to be important for organogenesis and cell proliferation through binding with the m^6^A site via the YTH domain and affecting mRNA stability, and have been reported to play a role in modifying tiller numbers^118^.

Grain yield depends on various spike-related traits such as the spike length, number of spikelets per spike, grains per spike and thousand-grain weight^119^. Targeting such traits has shown fruitful results in improving grain yield over the last decades. Two QTNs (**AX94650173** and **AX94707304)** were identified for spike length (SL) that were linked to three putative high-confidence protein-coding genes. SNP **AX94650173** (21.89% PVE) on Chr2A was found to be linked to gene **TraesCS2A02G212200** translating for “**Transcription factor UDT1**” and SNP **AX94707304** (26.90% PVE) on Chr6B was linked with two genes **TraesCS6B02G360300** and **TraesCS6B02G360500** which code for proteins “**Chloroplastic Soluble inorganic pyrophosphatase 6**” and “**WAT1-related protein At2g39510**”. The WALLS ARE THIN 1 (WAT1) gene has been reported to play a key role in cell elongation through auxin transport, which signifies its effect on spike length modification^120^. Interestingly, many QTLs for spike length have been reported to be present in the 2AS region where SNP **AX94650173** was found to be associated with SL in our present study^121^. The role played by these proteins in regulating spike length has been reported by Ko et al.^122^; Müller et al.^123^ and Nazari et al.^124^. The identification of SNP **AX94650173** under CTLS may indicate its role in regulating SL under stress conditions. Genes **TraesCS6B02G360300** and **TraesCS6B02G360500** were also found to be linked to SNP **AX94669346** (11.10% PVE) on Chr6B regulating the number of spikelets per spike (SPS). SNP **AX94882016** (17.17% PVE) on Chr3B was found to be associated with grains per spike (GPS) linked with genes **TraesCS3B02G303600**, **TraesCS3B02G304300** and **TraesCS3B02G304200** which translates for proteins “**Aldehyde oxidase *GLOX1***”, “**Protein MALE DISCOVERER 1**” and “**Rop guanine nucleotide exchange factor 11**”. It has been demonstrated that mutation and overexpression of an aldehyde oxidase gene *OsAO3* increased and decreased grain yield, respectively, by affecting panicle number per plant, spikelet number per panicle and spikelet fertility in rice^125^ and plants tend to downregulate the expression of *GLOX1* gene under stress condition as it has detrimental effects on pollen and pistil^126^. The MALE DISCOVERER 1 (*MDIS1*) gene is a receptor-like kinase that has been reported to play a key role in guiding the pollen tube growth by sensing the LURE signals during fertilisation in *Arabidopsis*^127^. A Rop guanine nucleotide exchange factor (*OsRopGEF7B*) was demonstrated to affect floral organ development and seed setting rate in Rice^128^. This SNP being identified under CTLS and the linked genes being expressed under stress conditions further signifies and makes their utilisation more evident in targeting stress tolerance and grain yield improvement in wheat.

Another key component trait that decides the grain yield is the thousand-grain weight (TGW) which highly depends upon the grain filling rate^129^. Two QTNs for TGW were identified where SNP **AX94879209** on Chr3D was found to be linked with “**Sodium/calcium exchanger NCL1**” and “**Basic leucine zipper 2**” genes which play important functions in calcium homeostasis and abiotic stress tolerance^130–133^ while SNP AX94543129 on Chr6D was linked to a “**SAP-like protein BP-73**” which is reported to regulate cell proliferation in developing seeds^134,135^. The identification of *NCL1* and *bZIP* gene associated QTN in CTTS makes it more evident that plants grown in CA environment are less exposed to stress compared to CT environment. For grain yield (GY), three SNPs viz., **AX94514123**, **AX94803899** and **AX94819762** were found to be linked to genes that code for proteins and protein families such as “**NAC domain-containing protein 22**”, “**Beta-1,2-xylosyltransferease XAX1**”, “**Protein PSK SIMULATOR 1**” and “**Transcription factor MYB44**” under CTTS environment. The roles of these proteins in regulating the grain yield under abiotic stress through modifying root architecture^136–138^, by regulating the xylan biosynthesis in the cell wall^139^, cell proliferation and light stress^139,140^, and stress tolerance by regulating stomatal development^142–144^. SNP **AX94525104** (31.33% PVE) on Chr3A was found to be linked to genes **TraesCS3A02G408200** and **TraesCS3A02G408000** which translates for proteins “**Bidirectional sugar transporter SWEET1a**” and “**Scarecrow-like protein 1**”, respectively which play a key role in regulating the grain length (GL) through enhanced grain filling rates^145–147^ and promoting gibberellin signalling through repression of DELLA repressor protein^148–150^. The identification of this QTN under the CALS environment and the genes annotated being reported to play a pivotal role in regulating grain yield under stress conditions signifies the importance of this QTN in improving grain yield under stress conditions. Two SNPs viz., **AX95194941** (6.56% PVE) on Chr5B and **AX94857819** (27.66% PVE) on Chr7D which translates for “**Oil body-associated protein 1A**” and “**Bifunctional monodehydroascorbate reductase and carbonic anhydrase nectarin-3**”, respectively were found to be associated with grain breadth (GB) and play roles in regulating grain yield^151–154^. The SNP **AX94857819** being identified under CALS emphasizes its importance in grain filling and stress tolerance.

In conclusion, this is the first report where we have identified key physiological traits viz., Phi2, NPQ, RC and PS1 which are responsible for improved performance of the genotypes under CA as compared to CT. These traits are the traits of adaptability under CA. Out of the 80 MTAs identified through GWAS, 48 were found to be highly associated with our traits of the present study as identified through *in-silico* gene annotation studies. The candidate genes with pivotal roles in trait development such as **Chloroplastic Photosystem II reaction center W protein** for Phi2 and **Bidirectional sugar transporter SWEET1a** for GL were identified through gene annotation studies. Upon validation, the identified QTNs hold potential for integration into marker-assisted breeding programs, contributing towards the development of wheat genotypes adapted to Conservation Agriculture.

### Supplementary Information

Supplementary Information.

## Data Availability

The datasets generated during and/or analyzed during the current study are available from the corresponding author upon reasonable request.

## Code availability

Publicly available statistical tools and databases were used for various analyses in the present study.
