# Supplementary material for: Discovering novel genomic regions explaining adaptation of bread wheat to conservation agriculture through GWAS
Source: Sci Rep. 2024 Jul 16;14:16351. doi: 10.1038/s41598-024-66903-3 (PMC11252282; doi:10.1038/s41598-024-66903-3)
Supplement: Supplementary file 1 — Supplementary Information. [file 41598_2024_66903_MOESM1_ESM.pdf]

Discovering novel genomic regions explaining adaptation of bread wheat to conservation agriculture through GWAS

Amit Kumar Mazumder[1], Rajbir Yadav[1], Manjeet Kumar[1], Prashanth Babu[1], Naresh Kumar[1], Sanjay Kumar Singh[1], Amolkumar U. Solanke[2], Shabir H. Wani[3], Adel I. Alalawy[4], Abdulrahman Alasmari[5], Kiran B. Gaikwad[1]*

[1]Division of Genetics, ICAR-Indian Agricultural Research Institute, New Delhi-110012, India

[2]National Institute for Plant Biotechnology, New Delhi-110012, India

[3]Mountain Research Centre for Field Crops, Khudwani-192101; Sher-e-Kashmir University of Agricultural Sciences and Technology-Kashmir (SKUAST-K), Srinagar, Jammu-Kashmir, India

[4]Department of Biochemistry, Faculty of Science, University of Tabuk, Saudi

[5]Department of Biology, Faculty of Science, University of Tabuk, Saudi

*Corresponding author: gaikwadkb@gmail.com (Orcid ID: 0000-0002-9076-8640)

SUPPLEMENTARY TABLES

Supplementary Table S1 Parentage of the 183 advanced breeding lines and the 5 commercial check varieties used in the association panel

Genotype ID	Parentage	Genotype ID	Parentage
217	HDCSW18	340	HD2733/HD2329//CL1449/PBW502
218	CSW176	341	CL2596/K9451/CL882//HD2009
219	BMZ135(2018-19)	342	CSW2/Yr15
222	BMZ125(2018-19)	343	PGT-15
223	BMZ146(2018-19)	344	PGT-48
224	BMZ115(2018-19)	345	HD2824/MC-11//PBW343/DW671
225	BMZ141(2018-19)	346	DL-5/PBW343//Deepali
226	BMZ101(2018-19)	347	HD2824/HD2643//HDK10/PBW502
227	HD3317	349	NIAW34/PHW12//43IBWSN-1187
228	SPL HYPT 10(2018-19)	350	HD2329/HD2285//43IBWSN-1182
229	SPL HYPT 10(2018-19)	351	CSW107
230	SPL HYPT 8(2018-19)	356	HD3313
231	SPL HYPT 8(2018-19)	359	WH1105/HD2329+Yr10
232	HD3086	360	HD3086/HD2932+Yr10
233	DPW621-50	361	HD3086/HD2932+Yr10
234	VL616(2)/Inqulab/ Kundan	362	HD2967/PBW550//HD2967+Yr10
235	DBW88	364	HDCSW18//HD2932+Yr10
236	DW1272/HP1731//43-IBWSN-1102	366	HDCSW18//HD2932+Yr10
237	31-ESWYT147/3/HW5028//HD2432/DW1309	368	SAWYT-319(06-07)//HD2932+Yr10
240	23 HRWYT-06	369	CL1449/HUW585//HD2932+Yr10
241	25 HRWSN 54	370	SAWYT-319(06-07)//HD2932+Yr10
242	CSW57/IND-EMT-12145-55	371	CL1449/HUW585//HD2932+Yr10
244	GS 10060	374	HD2967/NIVT-1A(3)//HD2932+Yr10
245	24 HRWSN 2112	376	18SAWYT-311//HD2932+Yr10
246	HD2874/HD2967//43IBWSN-1087	377	CL2596/K9461/CL882//HD2009//HD2932+Yr10
249	HD2967//HD2887/HD2946//HD2733	378	CL1705/HD2687//HD2932+Yr10
250	22ND HRWSN-2112	379	2ndWYCVT37/IDSN-45
251	HD2967//HD2887/HD2946//HD2733	380	WH1105/44IDSN-7061
252	DWG107/Raj3765//CSW 49	381	HD3117/HW5207
253	EBWYT-47	382	HD3117/HW5207
254	HDCSW18 (VARIANT)	383	HD3117/HW5207

257	31ESWYT135//HD2329/WR544/PBW343/NW3041	384	HD3117/HW5207
259	CL264/CL1633//CNo.601	385	HD2967/PBW550//HD2967+Yr10
260	IRSBWYT-40	386	2ndWYCYT 37/IDSN-45
261	CL1705/HD2687	387	WH1105/WH1157
262	CL2596/K9451//HS590	389	WH1105/PBW667
263	HDCSW18/PBW677	390	PBW712/WH1169
264	SAWYT-326/HD2967	391	PBW712/WH1169
265	NA	392	PBW712/WH1170
266	HD2967/HD2887//HD2946/HD2733	393	PBW712/WH1170
267	HD2967/HD2887//HD2946/HD2733	394	HD3086/WH1169
268	HD2967/HD2887//HD2946/HD2733	395	HDCSW18/WH1105
269	HD2967/HD2887//HD2946/HD2733	397	HDCSW18/WH1157
270	HD2967/CP196	399	HDCSW18/K1204
271	22ndHRWSN-2112	401	HDCSW18/K1204
272	SAWYT-308	402	HDCSW18/PBW712
273	PBW343/CL1538/HD2932/HD2189	404	HDCS18/WH1169
275	HD3312	405	HDCS18/WH1169
276	HD2932	406	WH1105/PBW698
277	HD2329	408	HD2967/HD3003//PBW712
280	WH542/UP2425//WH542/UP2425	409	HD2967/HD3002//K1204
281	IM-15/HD2967	410	HD2967/HD3002//HS592
282	CSW3/HD2932+Yr10	411	HD2967/HD3002//HS592
283	SAWSN-3194	412	PBW343/Picilocal/R2608//HD2932
284	24thHRWSN-2112	416	CL2596/K9451/CL882//HD2009//PBW677
285	NA	419	SAWYT(06-07)-319//WH1157
287	CL1633/CNo-601//CL1633/CNo-601	420	SAWYT(06-07)-319//PBW698
289	18HRWYT214/18 HRWYT-229	421	CL1449/HUW585/PBW698
290	SAWYT-326/HD2967	424	HD2967//HD2887/HD2946//HD2733
291	VL849/NBP-39//HD2967	425	43IBWSN-1175
292	HD2789/HD2891//HD2932	426	CL2596/K9451/CL882//HD2009
293	HD2967//HD2887/HD2946//HD2733	427	DLS/PBW343//Deepali
294	HD2967//HD2887/HD2946//HD2733	428	CL1705/HD2687
295	HD2967//HD2887/HD2946//HD2733	429	18HRWYT-214/18HRWYT-229
296	CSW24/CSW26	431	HD2967//HD2887/HD2946//HD2733
297	HD2877/DW433//WH542/3/HD2982//2967	432	31-ESWYT-135//HD2329/WR-544/PBW343/NW3041
298	HD2967/CP196	434	HD3115/PBW550

300	HD2967/CP196	437	7935 IN 13-14/ HD2967
301	IM-15/HD2967	439	CSW18/WR544
302	26SAWYT-309	440	NA
303	26SAWYT-314	441	NA
304	26SAWYT-316	442	HD2967//HD2887/HD2946//HD2733
305	26SAWYT-319	443	HD2967//HD2887/HD2946//HD2733
307	ESWYT-45	445	HD2967//HD2887/HD2946//HD2733
308	SAWYT-342	447	NA
309	38ESWYT-149	449	NA
310	25 SAWYT-343	450	NA
311	DW1272/HP1731//43-IBWSN-1102	451	NA
314	HD2967//HD2887/HD2946//HD2733	453	CL264/CL1633//CNO.601
316	HD2967/CP196	455	DL-5/PBW343//Deepali
318	SAWYT-326/HD2967	456	CSW2/Yr15
321	CSW2/Yr15	458	VL849/NBP-39//HD2967
322	CSW18/CSW1	459	HD2967/HD2887//HD2946/HD2733
323	CSW18/CSW1	461	SAWYT(06-07)-319/WH1170
325	HD2967	462	SAWYT(06-07)-319/PBW677
326	CL1705/HD2687	463	18thSAWYT-311//K1204
327	CL1633/CNo-601//CL1633/CNo-601	464	CL1705/HD2687//WH1169
329	SRRSN-6083	466	18HRWYT-214//HI1563
331	23 HRWSN-2003	467	18HRWYT-214//HI1563
335	HDCSW18/PBW677	DBW187	NAC/TH.AC//3*PVN/3/MIRLO/BUC/4/2*PASTOR/5/KACHU/6/KACHU
336	CL2596/K9451/CL882//HD2009//WH1170	HD3086	DBW14/HD2733//HUW468
337	HD2967//HD2887/HD2946//HD2733	HD3226	GRACKLE/HD2894
338	CBW38/WR541//VHN4168	HD3298	CL1449/PBW343//CL882/HD2009
339	SAWSN-3162	HI1621	W15.92/4/PASTOR//HXL7573/2*BAU/3/WBLL1

Supplementary Table S2 Descriptive statistics for the 19 morpho-physiological traits under the four production environments

Trait	Count	CTTS				CATS				CTLS				CALS			
		Mean ± SD	Min	Max	CV	Mean ± SD	Min	Max	CV	Mean ± SD	Min	Max	CV	Mean ± SD	Min	Max	CV
Phi2	188	0.320 ± 0.046	0.203	0.436	5.654	0.373 ± 0.041	0.24	0.484	3.621	0.278 ± 0.056	0.143	0.444	3.964	0.307 ± 0.049	0.2	0.447	9.613
NPQ	188	0.514 ± 0.057	0.365	0.636	5.705	0.445 ± 0.045	0.311	0.59	3.986	0.584 ± 0.069	0.378	0.743	4.804	0.53 ± 0.073	0.333	0.671	7.32
RC	188	55.055 ± 5.393	42.758	66.85	3.746	55.513 ± 7.234	36.551	69.595	2.981	51.71 ± 6.651	33.849	66.475	2.916	54.47 ± 6.171	30.643	68.418	2.549
PS1	188	2.207 ± 0.627	0.617	4.166	11.473	2.452 ± 0.655	0.688	4.346	13.098	2.149 ± 0.65	0.000	3.927	11.031	2.227 ± 0.547	0.872	3.984	9.186
CTD	188	0.935 ± 0.764	-1.457	2.413	5.489	0.906 ± 0.559	-0.68	2.47	22.048	0.893 ± 0.611	-0.484	2.259	10.271	1.398 ± 0.704	-1.15	2.806	3.361
LT	188	0.134 ± 0.025	0.088	0.316	4.01	0.137 ± 0.026	0.081	0.21	2.827	0.13 ± 0.023	0.084	0.199	2.943	0.132 ± 0.025	0.087	0.235	2.699
LA	188	62.443 ± 5.783	47.45	80.139	4.053	62.04 ± 6.434	45.14	79.205	3.082	63.714 ± 8.194	36.887	83.987	3.972	59.404 ± 8.43	39.178	77.965	6.482
DTH	188	97.964 ± 8.273	77.653	115.333	4.14	97.709 ± 8.143	79.495	116.878	3.159	79.656 ± 8.387	62.536	96.570	2.889	78.998 ± 7.414	61.69	94.994	2.284
DTM	188	145.642 ± 9.926	119.298	165.048	3.333	150.466 ± 12.655	125.704	179.959	2.949	114.866 ± 11.507	89.125	141.036	2.686	113.908 ± 9.799	91.057	135.657	2.271
PH	188	107.542 ± 11.467	73.503	132.217	3.442	95.726 ± 13.22	66.196	131.611	3.326	79.694 ± 9.357	54.670	108.327	3.716	82.387 ± 13.038	47.35	113.164	3.135
TC	188	171.824 ± 45.061	54.392	310.629	5.27	141.787 ± 39.678	50.22	246.157	3.373	128.888 ± 43.5	17.754	252.043	3.176	117.309 ± 42.388	16.798	228.176	2.853
SL	188	10.468 ± 1.281	7.895	16.672	3.477	11.222 ± 1.764	7.686	16.866	2.976	9.794 ± 1.399	6.179	13.250	3.191	9.901 ± 1.459	6.081	13.755	3.09
SPS	188	19.363 ± 2.149	13.789	26.1	3.685	21.046 ± 2.349	16.07	27.542	3.734	17.941 ± 1.847	13.989	24.351	2.717	21.268 ± 2.283	14.831	27.452	2.198
GPS	188	58.407 ± 11.211	23.295	89.815	10.218	64.991 ± 11.955	36.073	100.13	10.621	58.793 ± 9.983	30.137	89.439	8.042	60.088 ± 11.693	15.093	87.641	2.948
TGW	188	41.489 ± 9.001	11.64	64.714	14.942	42.621 ± 7.37	24.579	66.511	8.28	40.67 ± 5.812	24.282	55.799	10.055	40.84 ± 9.009	5.935	62.228	3.368
GY	188	68.798 ± 16.658	35.212	104.576	9.229	76.151 ± 14.814	31.795	107.076	3.187	41.615 ± 11.527	6.532	69.711	18.165	49.268 ± 16.231	17.91	79.125	4.789
GL	188	6.737 ± 0.509	5.143	8.146	4.115	6.892 ± 0.78	5.097	8.728	2.859	6.726 ± 0.721	5.162	8.217	2.599	6.666 ± 0.734	4.383	8.548	3.223
GB	188	3.749 ± 0.366	2.564	4.516	4.257	3.826 ± 0.43	2.322	4.733	3.507	3.749 ± 0.388	2.925	4.704	3.098	3.784 ± 0.522	1.678	4.934	2.885
GSA	188	19.243 ± 2.368	13.449	24.622	4.515	20.149 ± 2.736	14.17	26.939	3.624	19.228 ± 2.45	14.052	25.668	3.267	19.259 ± 3.36	5.734	28.958	3.702

Phi2, PS II quantum yield; NPQ, non-photochemical quenching; RC, relative chlorophyll; CTD, canopy temperature depression; PS1, active PS I center; LT, leaf thickness; LA, leaf angle; DTH, days to heading; DTM, days to maturity; PH, plant height; TC, tiller count; SL, spike length; SPS, spikelets per spike; GPS, grains per spike; TGW, thousand-grain weight; GY, grain yield; GL, grain length; GB, grain breadth; GSA, grain surface area; CTTS, conventional tillage timely sown; CATS, conservation agriculture timely sown; CTLS, conventional tillage late sown; CALS, conservation agriculture late sown

Supplementary Table S3a Block adjusted ANOVA for morpho-physiological under CTTS production environment

Source	Df	Mean Square																		
		Phi2	NPQ	RC	PS1	CTD	LT	LA	DTH	DTM	PH	TC	SL	SPS	GPS	TGW	GY	GL	GB	GSA
Treatment (ignoring Blocks)	187	0.00188**	0.00413**	15.26051**	0.38961**	0.64865**	0.00056**	33.9858**	54.59375**	57.41952**	107.98113**	1768.91859**	1.61217**	5.00962**	128.82109**	67.36946**	320.64405**	0.17612**	0.09265**	5.12131**
Treatment: Check	4	0.00017**	0.03654**	43.08452**	1.41096**	1.60606**	0.00074**	171.9782**	278.19367**	488.27978**	50.12526**	305.34705**	3.90706**	16.66903**	743.2323**	31.5388**	348.0978**	0.02926**	0.28557**	19.90134**
Treatment: Test	182	0.00181**	0.00303**	14.7222**	0.36681**	0.59419**	0.00055**	30.31247**	45.27983**	47.88801**	106.12722**	1711.26308**	1.57058**	4.61155**	112.61801**	64.15385**	276.02518**	0.14137**	0.0856**	4.58533**
Treatment: Test vs. Check	1	0.02173**	0.07555**	1.93602**	0.45276**	6.73025**	0.00154**	150.56276**	855.32827**	68.71353**	676.81746**	18116.50808**	0.00277**	30.82161**	620.13634**	795.93441**	8331.46362**	7.08922**	0.60525**	43.55036**
Block (eliminating Treatments)	7	0.00353**	0.00305**	78.84915**	0.27032**	0.0089**	0.00052**	121.71911**	207.82464**	329.42587**	310.46563**	975.90726**	2.83598**	10.09757**	237.7994**	183.20889**	115.65832**	0.67826**	0.43631**	11.42837**
Residuals	28	0.00034**	0.00084**	4.25744**	0.06303**	0.00226**	0.00003**	6.46716**	16.17671**	23.48215**	13.51942**	78.54832**	0.13237**	0.50109**	34.79494**	39.80217**	37.4897**	0.07844**	0.02574**	0.76821**

Phi2, PS II quantum yield; NPQ, non-photochemical quenching; RC, relative chlorophyll; CTD, canopy temperature depression; PS1, active PS I center; LT, leaf thickness; LA, leaf angle; DTH, days to heading; DTM, days to maturity; PH, plant height; TC, tiller count; SL, spike length; SPS, spikelets per spike; GPS, grains per spike; TGW, thousand-grain weight; GY, grain yield; GL, grain length; GB, grain breadth; GSA, grain surface area; CTTS, conventional tillage timely sown; CATS, conservation agriculture timely sown; CTLS, conventional tillage late sown; CALS, conservation agriculture late sown

^{ns} $P > 0.05$; * $P \leq 0.05$; ** $P \leq 0.01$

Supplementary Table S3b Block adjusted ANOVA for morpho-physiological under CATS production environment.

Source	Df	Mean Square																		
		Phi2	NPQ	RC	PS1	CTD	LT	LA	DTH	DTM	PH	TC	SL	SPS	GPS	TGW	GY	GL	GB	GSA
Treatment (ignoring Blocks)	187	0.00138**	0.00339**	19.27153**	0.49121**	0.31473**	0.00039**	30.42953**	35.62955**	42.33688**	94.89926**	1315.08341**	2.13678**	4.08386**	137.72424**	42.22046**	202.61735**	0.18723**	0.08598**	4.09612**
Treatment: Check	4	0.00154**	0.04638**	71.72107**	0.57568**	0.76034**	0.00091**	173.74912**	76.83011**	44.97707^{ns}	96.59257**	302.45557**	7.56175**	19.82061**	333.9694**	34.15782^{ns}	517.56039**	0.06256^{ns}	0.43976**	18.4053**
Treatment: Test	182	0.00138**	0.00228**	17.5508**	0.43747**	0.30574**	0.00037**	23.46383**	34.62049**	42.45956**	87.06653**	1338.44358**	1.96068**	3.76043**	132.96271**	39.54392**	188.6436**	0.17646**	0.07581**	3.51657**
Treatment: Test vs. Check	1	0.00074^{ns}	0.03205**	122.64631**	9.93366**	0.16866^{ns}	0.00326**	724.90701**	54.47561*	9.4492ns	1513.68178**	1114.0433**	12.48749**	0.00191ns	219.34264*	561.60305**	1486.06842**	2.64492**	0.52161**	52.33891**
Block (eliminating Treatments)	7	0.0058**	0.00472**	133.02577**	0.39519**	0.03041^{ns}	0.00083**	175.25851**	322.00782**	869.1573**	410.27957**	805.06637**	5.22292**	14.46144**	84.7496^{ns}	62.75863**	188.66314**	2.12714**	0.56934**	16.90493**
Residuals	28	0.00018	0.00031	2.76673	0.11028	0.04083	0.00002	3.74146	9.48926	19.66836	10.35418	23.15454	0.11343	0.61768	47.08121	12.82836	5.73041	0.03931	0.01818	0.54358

Phi2, PS II quantum yield; NPQ, non-photochemical quenching; RC, relative chlorophyll; CTD, canopy temperature depression; PS1, active PS I center; LT, leaf thickness; LA, leaf angle; DTH, days to heading; DTM, days to maturity; PH, plant height; TC, tiller count; SL, spike length; SPS, spikelets per spike; GPS, grains per spike; TGW, thousand-grain weight; GY, grain yield; GL, grain length; GB, grain breadth; GSA, grain surface area; CTTS, conventional tillage timely sown; CATS, conservation agriculture timely sown; CTLS, conventional tillage late sown; CALS, conservation agriculture late sown

^{ns} $P > 0.05$; * $P \leq 0.05$; ** $P \leq 0.01$

Supplementary Table S3c Block adjusted ANOVA for morpho-physiological under CTLS production environment.

Source	Df	Mean Square																		
		**Phi2**	**NPQ**	**RC**	**PS1**	**CTD**	**LT**	**LA**	**DTH**	**DTM**	**PH**	**TC**	**SL**	**SPS**	**GPS**	**TGW**	**GY**	**GL**	**GB**	**GSA**
Treatment (ignoring Blocks)	187	0.00173**	0.00251**	26.42936**	0.40914**	0.42372**	0.00028**	36.63146**	29.46939**	41.4556**	75.77157**	1662.96243**	1.24039**	2.03549**	96.41009**	27.08983^{ns}	139.95146**	0.12109**	0.05255**	3.2449**
Treatment: Check	4	0.00065**	0.00274*	189.48164**	1.47897**	1.03425**	0.00034**	93.878**	24.17842**	60.53644**	67.4875**	141.92071**	5.4712**	7.85718**	184.92732**	14.52986^{ns}	13.96217^{ns}	0.27319**	0.58513**	17.3272**
Treatment: Test	182	0.00141**	0.00205**	22.48774**	0.38746**	0.35046**	0.00027**	35.42427**	29.16742**	41.25743**	44.14651**	1705.09875**	1.13249**	1.89384**	87.23709**	27.17717^{ns}	141.40242**	0.11754**	0.04049**	2.9298**
Treatment: Test vs. Check	1	0.06446**	0.08453**	91.59418**	0.07488^{ns}	11.31454**	0.00145**	27.35268*	105.59209**	1.19818^{ns}	5864.66969**	78.31978*	3.95522**	4.52936**	1411.82708**	61.43375^{ns}	379.83462*	0.15972*	0.11712**	4.26289**
Block (eliminating Treatments)	7	0.00476**	0.00952**	102.45666**	0.14388*	0.00995^{ns}	0.00076**	167.52951**	228.26055**	499.34522**	320.66774**	596.94352**	4.54763**	11.55241**	107.01853**	30.11361^{ns}	28.73076^{ns}	1.90339**	0.58793**	13.06819**
Residuals	28	0.00	0.00	2.25	0.06	0.01	0.00	6.37	5.26	9.51	9.22	16.70	0.10	0.24	21.59	16.89	58.56	0.03	0.01	0.39

Phi2, PS II quantum yield; NPQ, non-photochemical quenching; RC, relative chlorophyll; CTD, canopy temperature depression; PS1, active PS I center; LT, leaf thickness; LA, leaf angle; DTH, days to heading; DTM, days to maturity; PH, plant height; TC, tiller count; SL, spike length; SPS, spikelets per spike; GPS, grains per spike; TGW, thousand-grain weight; GY, grain yield; GL, grain length; GB, grain breadth; GSA, grain surface area; CTTS, conventional tillage timely sown; CATS, conservation agriculture timely sown; CTLS, conventional tillage late sown; CALS, conservation agriculture late sown

^{ns} P > 0.05; * P ≤ 0.05; ** P ≤ 0.01

Supplementary Table S3d Block adjusted ANOVA for morpho-physiological under CALS production environment

Source	Df	Mean Square																		
		Phi2	NPQ	RC	PS1	CTD	LT	LA	DTH	DTM	PH	TC	SL	SPS	GPS	TGW	GY	GL	GB	GSA
Treatment (ignoring Blocks)	187	0.00322**	0.00728**	18.47425**	0.33521**	0.55439**	0.00046**	42.6709**	21.86437**	32.21837**	91.92614**	1540.71665**	1.66258**	3.5206**	125.55394**	56.63905**	224.98641**	0.14533**	0.12705**	6.02816**
Treatment: Check	4	0.00512**	0.00351^{ns}	43.95006**	1.37184**	0.46854**	0.0005**	41.94603*	20.55285**	28.07094**	24.01856*	166.6107**	11.80428**	4.42126**	182.94996**	36.3744**	39.38374**	0.15899*	0.19353**	7.30592**
Treatment: Test	182	0.00207**	0.00503**	17.99491**	0.31408**	0.47273**	0.00046**	38.29458**	21.99448**	30.14454**	62.33518**	1571.66348**	1.32056**	2.865**	119.00398**	55.03285**	228.9141**	0.13004**	0.10215**	5.18584**
Treatment: Test vs. Check	1	0.20428**	0.43191**	3.81143^{ns}	0.0346ns	15.7612**	0.00019**	842.06046**	3.43053^{ns}	426.2457**	5749.11092**	1404.81655**	23.34265**	119.23818**	1088.06217**	430.02687**	252.55635**	2.87367**	4.39295**	154.21935**
Block (eliminating Treatments)	7	0.00202^{ns}	0.0022^{ns}	121.19937**	0.29085**	0.02202**	0.00073**	169.70466**	247.98665**	481.1663**	352.6931**	680.27743**	4.46243**	13.82986**	160.13482**	81.308**	132.14902**	2.02694**	0.68244**	23.54738**
Residuals	28	0.00094	0.00141	1.9307	0.04168	0.00189	0.00001	15.21701	3.25187	6.75448	7.00408	11.39519	0.09603	0.21261	3.04691	1.94341	5.47082	0.0468	0.01227	0.52586

Phi2, PS II quantum yield; NPQ, non-photochemical quenching; RC, relative chlorophyll; CTD, canopy temperature depression; PS1, active PS I center; LT, leaf thickness; LA, leaf angle; DTH, days to heading; DTM, days to maturity; PH, plant height; TC, tiller count; SL, spike length; SPS, spikelets per spike; GPS, grains per spike; TGW, thousand-grain weight; GY, grain yield; GL, grain length; GB, grain breadth; GSA, grain surface area; CTTS, conventional tillage timely sown; CATS, conservation agriculture timely sown; CTLS, conventional tillage late sown; CALS, conservation agriculture late sown

^{ns} P > 0.05; * P ≤ 0.05; ** P ≤ 0.01

Supplementary Table S4 Pearson's correlation matrix of the 19 morpho-physiological traits under CTTS production environment

	Phi2	NPQ	RC	CTD	PS1	LT	LA	DTH	DTM	PH	TC	SL	SPS	GPS	TGW	GY	GL	GB	GSA
**Phi2**	1	-0.9***	0.03	-0.12	0.04	-0.23**	0.01	-0.02	-0.04	-0.08	-0.08	0	0.05	0.13	-0.01	-0.04	0.01	-0.02	0
**NPQ**	-0.9***	1	-0.1	0.14	-0.08	0.26***	-0.07	0.1	0.13	0.14	0.1	0.03	0.03	-0.09	-0.01	-0.01	-0.04	-0.01	-0.04
**RC**	0.03	-0.1	1	-0.06	0.24***	0.1	-0.11	-0.13	-0.16*	-0.29***	0.02	-0.14	-0.21**	-0.14	-0.06	0.14	0.05	-0.06	-0.06
**CTD**	-0.12	0.14	-0.06	1	-0.06	0.21**	0.14	-0.02	-0.01	0.08	0.03	0.09	-0.02	-0.03	-0.02	-0.06	0.05	-0.03	0
**PS1**	0.04	-0.08	0.24***	-0.06	1	-0.09	0.03	-0.01	-0.03	-0.09	0.02	-0.04	-0.16*	0.09	0.02	0.11	0.09	0.03	0.07
**LT**	-0.23**	0.26***	0.1	0.21**	-0.09	1	-0.08	-0.02	-0.02	0	0.06	0.03	0	-0.01	-0.11	-0.16*	-0.04	-0.15*	-0.13
**LA**	0.01	-0.07	-0.11	0.14	0.03	-0.08	1	-0.04	-0.04	0.06	-0.02	0.16*	0.09	0.03	-0.01	0.03	0.09	0.02	0.05
**DTH**	-0.02	0.1	-0.13	-0.02	-0.01	-0.02	-0.04	1	0.97***	0.4***	0.02	0.22**	0.46***	0.25***	-0.09	-0.19*	-0.1	-0.05	-0.08
**DTM**	-0.04	0.13	-0.16*	-0.01	-0.03	-0.02	-0.04	0.97***	1	0.37***	0.04	0.19**	0.43***	0.22**	-0.11	-0.17*	-0.1	-0.08	-0.1
**PH**	-0.08	0.14	-0.29***	0.08	-0.09	0	0.06	0.4***	0.37***	1	-0.11	0.41***	0.56***	0.28***	-0.01	-0.25***	0	0.09	0.07
**TC**	-0.08	0.1	0.02	0.03	0.02	0.06	-0.02	0.02	0.04	-0.11	1	-0.19**	-0.16*	-0.17*	-0.12	0.13	-0.1	-0.07	-0.13
**SL**	0	0.03	-0.14	0.09	-0.04	0.03	0.16*	0.22**	0.19**	0.41***	-0.19**	1	0.61***	0.24**	0.12	-0.12	0.16*	0.13	0.16*
**SPS**	0.05	0.03	-0.21**	-0.02	-0.16*	0	0.09	0.46***	0.43***	0.56***	-0.16*	0.61***	1	0.35***	0.06	-0.2**	-0.07	0.12	0.05
**GPS**	0.13	-0.09	-0.14	-0.03	0.09	-0.01	0.03	0.25***	0.22**	0.28***	-0.17*	0.24**	0.35***	1	0.02	-0.17*	0.01	0.17*	0.14*
**TGW**	-0.01	-0.01	-0.06	-0.02	0.02	-0.11	-0.01	-0.09	-0.11	-0.01	-0.12	0.12	0.06	0.02	1	0.26***	0.51***	0.77***	0.78***
**GY**	-0.04	-0.01	0.14	-0.06	0.11	-0.16*	0.03	-0.19*	-0.17*	-0.25***	0.13	-0.12	-0.2**	-0.17*	0.26***	1	0.17*	0.2**	0.22**
**GL**	0.01	-0.04	0.05	0.05	0.09	-0.04	0.09	-0.1	-0.1	0	-0.1	0.16*	-0.07	0.01	0.51***	0.17*	1	0.58***	0.8***
**GB**	-0.02	-0.01	-0.06	-0.03	0.03	-0.15*	0.02	-0.05	-0.08	0.09	-0.07	0.13	0.12	0.17*	0.77***	0.2**	0.58***	1	0.91***
**GSA**	0	-0.04	-0.06	0	0.07	-0.13	0.05	-0.08	-0.1	0.07	-0.13	0.16*	0.05	0.14*	0.78***	0.22**	0.8***	0.91***	1

Phi2, PS II quantum yield; NPQ, non-photochemical quenching; RC, relative chlorophyll; CTD, canopy temperature depression; PS1, active PS I center; LT, leaf thickness; LA, leaf angle; DTH, days to heading; DTM, days to maturity; PH, plant height; TC, tiller count; SL, spike length; SPS, spikelets per spike; GPS, grains per spike; TGW, thousand-grain weight; GY, grain yield; GL, grain length; GB, grain breadth; GSA, grain surface area

* $P \leq 0.05$; ** $P \leq 0.01$; *** $P \leq 0.001$

Supplementary Table S5 Pearson's correlation matrix of the 19 morpho-physiological traits under CATS production environment

	Phi2	NPQ	RC	CTD	PS1	LT	LA	DTH	DTM	PH	TC	SL	SPS	GPS	TGW	GY	GL	GB	GSA
**Phi2**	1	-0.88***	-0.18*	0.03	0.08	0	0.36***	0.24***	0.24**	0.19**	0.2**	0.01	0.19**	-0.05	-0.02	0.09	-0.08	-0.05	-0.11
**NPQ**	-0.88***	1	0.1	-0.03	-0.24***	0.05	-0.3***	-0.18*	-0.17*	-0.16*	-0.12	0	-0.16*	0	0.01	-0.07	0.06	-0.02	0.06
**RC**	-0.18*	0.1	1	-0.25***	0.03	0.25***	-0.2**	-0.29***	-0.27***	-0.23**	-0.11	0.06	-0.05	0.12	0.13	-0.06	0.08	0.1	0.09
**CTD**	0.03	-0.03	-0.25***	1	0.07	-0.05	0.05	0.19**	0.18*	-0.05	-0.25***	-0.02	-0.01	0.02	-0.04	-0.09	0.02	-0.12	-0.05
**PS1**	0.08	-0.24***	0.03	0.07	1	-0.08	-0.05	0.11	0.04	0.06	-0.13	0.03	0	0.05	0.08	-0.01	0	0.04	0.01
**LT**	0	0.05	0.25***	-0.05	-0.08	1	-0.07	-0.11	-0.06	0.07	0.12	0.13	-0.03	0	0.08	0.06	0.05	0.08	0.15*
**LA**	0.36***	-0.3***	-0.2**	0.05	-0.05	-0.07	1	0.16*	0.2**	0.01	0.17*	0.01	0.14*	-0.15*	-0.14	0	-0.14	-0.01	-0.14
**DTH**	0.24***	-0.18*	-0.29***	0.19**	0.11	-0.11	0.16*	1	0.9***	0.21**	0.07	0.12	0.15*	0.08	-0.04	0.02	0.02	-0.02	-0.06
**DTM**	0.24**	-0.17*	-0.27***	0.18*	0.04	-0.06	0.2**	0.9***	1	0.15*	0.06	0.1	0.11	0.01	-0.01	-0.02	0.04	-0.02	-0.02
**PH**	0.19**	-0.16*	-0.23**	-0.05	0.06	0.07	0.01	0.21**	0.15*	1	0.28***	0.31***	0.35***	0.04	0.06	0.22**	0	0.02	0.02
**TC**	0.2**	-0.12	-0.11	-0.25***	-0.13	0.12	0.17*	0.07	0.06	0.28***	1	0.05	0.1	-0.04	-0.13	0.28***	-0.14	-0.06	-0.18*
**SL**	0.01	0	0.06	-0.02	0.03	0.13	0.01	0.12	0.1	0.31***	0.05	1	0.57***	0.14	0.05	0.01	0.13	0.09	0.1
**SPS**	0.19**	-0.16*	-0.05	-0.01	0	-0.03	0.14*	0.15*	0.11	0.35***	0.1	0.57***	1	0.16*	-0.12	0.04	-0.15*	-0.02	-0.07
**GPS**	-0.05	0	0.12	0.02	0.05	0	-0.15*	0.08	0.01	0.04	-0.04	0.14	0.16*	1	-0.04	0.02	-0.05	0.07	0.03
**TGW**	-0.02	0.01	0.13	-0.04	0.08	0.08	-0.14	-0.04	-0.01	0.06	-0.13	0.05	-0.12	-0.04	1	-0.06	0.46***	0.48***	0.66***
**GY**	0.09	-0.07	-0.06	-0.09	-0.01	0.06	0	0.02	-0.02	0.22**	0.28***	0.01	0.04	0.02	-0.06	1	-0.05	-0.01	-0.09
**GL**	-0.08	0.06	0.08	0.02	0	0.05	-0.14	0.02	0.04	0	-0.14	0.13	-0.15*	-0.05	0.46***	-0.05	1	0.5***	0.72***
**GB**	-0.05	-0.02	0.1	-0.12	0.04	0.08	-0.01	-0.02	-0.02	0.02	-0.06	0.09	-0.02	0.07	0.48***	-0.01	0.5***	1	0.78***
**GSA**	-0.11	0.06	0.09	-0.05	0.01	0.15*	-0.14	-0.06	-0.02	0.02	-0.18*	0.1	-0.07	0.03	0.66***	-0.09	0.72***	0.78***	1

Phi2, PS II quantum yield; NPQ, non-photochemical quenching; RC, relative chlorophyll; CTD, canopy temperature depression; PS1, active PS I center; LT, leaf thickness; LA, leaf angle; DTH, days to heading; DTM, days to maturity; PH, plant height; TC, tiller count; SL, spike length; SPS, spikelets per spike; GPS, grains per spike; TGW, thousand-grain weight; GY, grain yield; GL, grain length; GB, grain breadth; GSA, grain surface area

* $P \leq 0.05$; ** $P \leq 0.01$; *** $P \leq 0.001$

Supplementary Table S6 Pearson's correlation matrix of the 19 morpho-physiological traits under CTLS production environment

	Phi2	NPQ	RC	CTD	PS1	LT	LA	DTH	DTM	PH	TC	SL	SPS	GPS	TGW	GY	GL	GB	GSA
**Phi2**	1	-0.9***	0.07	-0.32***	0.1	0.36***	0.26***	0.08	0.07	-0.16*	0.08	-0.03	-0.08	-0.15*	0.01	-0.34***	0.12	-0.02	0.04
**NPQ**	-0.9***	1	-0.17*	0.24***	-0.19*	-0.33***	-0.24***	-0.01	0.01	0.17*	-0.06	0.02	0.04	0.14	0.01	0.35***	-0.13	0.05	-0.03
**RC**	0.07	-0.17*	1	-0.13	0.31***	0.19**	-0.08	0.02	0.09	-0.21**	0.17*	0	-0.01	-0.05	0.07	-0.2**	0.12	0	0.07
**CTD**	-0.32***	0.24***	-0.13	1	0.01	-0.22**	0.1	-0.01	0.02	0.19*	-0.29***	0.12	0.12	0.14	0.03	0.17*	0.03	0.07	0.05
**PS1**	0.1	-0.19*	0.31***	0.01	1	0.16*	0.13	0.08	0.04	-0.05	0.03	0.15*	-0.01	-0.01	0.07	-0.05	0.15*	0.12	0.15*
**LT**	0.36***	-0.33***	0.19**	-0.22**	0.16*	1	-0.06	0.02	0.03	-0.19*	0.07	0.15*	-0.02	-0.11	-0.05	-0.3***	0.02	-0.07	-0.04
**LA**	0.26***	-0.24***	-0.08	0.1	0.13	-0.06	1	-0.07	-0.09	-0.16*	-0.15*	-0.03	-0.15*	-0.01	0.15*	-0.05	0.14*	0.25***	0.22**
**DTH**	0.08	-0.01	0.02	-0.01	0.08	0.02	-0.07	1	0.88***	0.03	-0.01	0.1	0.13	0.1	-0.2**	-0.16*	-0.12	-0.16*	-0.17*
**DTM**	0.07	0.01	0.09	0.02	0.04	0.03	-0.09	0.88***	1	0	0.05	0.08	0.11	0.09	-0.19*	-0.11	-0.16*	-0.15*	-0.18*
**PH**	-0.16*	0.17*	-0.21**	0.19*	-0.05	-0.19*	-0.16*	0.03	0	1	-0.15*	0.17*	0.36***	0.03	0.05	0.49***	-0.21**	-0.04	-0.13
**TC**	0.08	-0.06	0.17*	-0.29***	0.03	0.07	-0.15*	-0.01	0.05	-0.15*	1	-0.07	-0.05	0	-0.01	-0.04	-0.05	-0.08	-0.08
**SL**	-0.03	0.02	0	0.12	0.15*	0.15*	-0.03	0.1	0.08	0.17*	-0.07	1	0.56***	0.02	0.21**	0.02	0.15*	0.1	0.14
**SPS**	-0.08	0.04	-0.01	0.12	-0.01	-0.02	-0.15*	0.13	0.11	0.36***	-0.05	0.56***	1	0.14	0.02	0.2**	-0.13	0.01	-0.08
**GPS**	-0.15*	0.14	-0.05	0.14	-0.01	-0.11	-0.01	0.1	0.09	0.03	0	0.02	0.14	1	-0.17*	0.03	-0.06	0.06	-0.02
**TGW**	0.01	0.01	0.07	0.03	0.07	-0.05	0.15*	-0.2**	-0.19*	0.05	-0.01	0.21**	0.02	-0.17*	1	0.07	0.42***	0.67***	0.67***
**GY**	-0.34***	0.35***	-0.2**	0.17*	-0.05	-0.3***	-0.05	-0.16*	-0.11	0.49***	-0.04	0.02	0.2**	0.03	0.07	1	-0.18*	0.05	-0.06
**GL**	0.12	-0.13	0.12	0.03	0.15*	0.02	0.14*	-0.12	-0.16*	-0.21**	-0.05	0.15*	-0.13	-0.06	0.42***	-0.18*	1	0.52***	0.84***
**GB**	-0.02	0.05	0	0.07	0.12	-0.07	0.25***	-0.16*	-0.15*	-0.04	-0.08	0.1	0.01	0.06	0.67***	0.05	0.52***	1	0.87***
**GSA**	0.04	-0.03	0.07	0.05	0.15*	-0.04	0.22**	-0.17*	-0.18*	-0.13	-0.08	0.14	-0.08	-0.02	0.67***	-0.06	0.84***	0.87***	1

Phi2, PS II quantum yield; NPQ, non-photochemical quenching; RC, relative chlorophyll; CTD, canopy temperature depression; PS1, active PS I center; LT, leaf thickness; LA, leaf angle; DTH, days to heading; DTM, days to maturity; PH, plant height; TC, tiller count; SL, spike length; SPS, spikelets per spike; GPS, grains per spike; TGW, thousand-grain weight; GY, grain yield; GL, grain length; GB, grain breadth; GSA, grain surface area

* $P \le 0.05$; ** $P \le 0.01$; *** $P \le 0.001$

Supplementary Table S7 Pearson's correlation matrix of the 19 morpho-physiological traits under CALS production environment

	Phi2	NPQ	RC	CTD	PS1	LT	LA	DTH	DTM	PH	TC	SL	SPS	GPS	TGW	GY	GL	GB	GSA
**Phi2**	1	-0.92***	-0.04	-0.13	0.15*	0.24***	0.1	0.3***	0.27***	-0.27***	-0.12	-0.09	-0.18*	-0.12	-0.29***	-0.26***	-0.14	-0.31***	-0.27***
**NPQ**	-0.92***	1	0.07	0.1	-0.26***	-0.33***	0.01	-0.41***	-0.37***	0.36***	0.17*	0.09	0.18*	0.09	0.34***	0.35***	0.14	0.37***	0.3***
**RC**	-0.04	0.07	1	-0.13	0.15*	-0.02	-0.05	0.09	0.09	-0.03	-0.01	0.11	0.12	0.05	0.06	-0.08	0.21**	0.07	0.13
**CTD**	-0.13	0.1	-0.13	1	-0.11	-0.01	0.13	0.03	0.03	-0.02	-0.06	-0.05	0.07	0.13	-0.02	0.06	-0.14	0.1	-0.01
**PS1**	0.15*	-0.26***	0.15*	-0.11	1	-0.01	-0.15*	0.16*	0.18*	-0.12	-0.17*	-0.03	-0.03	0.08	-0.05	-0.19**	0.12	-0.09	0.02
**LT**	0.24***	-0.33***	-0.02	-0.01	-0.01	1	-0.15*	0.25***	0.18*	-0.22**	-0.05	-0.05	-0.08	0.01	-0.05	-0.28***	-0.02	-0.17*	-0.1
**LA**	0.1	0.01	-0.05	0.13	-0.15*	-0.15*	1	-0.18*	-0.2**	0.11	-0.06	-0.03	0.08	-0.18*	0.02	0.18*	0.01	0.14	0.04
**DTH**	0.3***	-0.41***	0.09	0.03	0.16*	0.25***	-0.18*	1	0.83***	-0.4***	-0.24***	0.12	0.08	0.13	-0.44***	-0.37***	-0.17*	-0.4***	-0.34***
**DTM**	0.27***	-0.37***	0.09	0.03	0.18*	0.18*	-0.2**	0.83***	1	-0.31***	-0.15*	0.1	0.01	0.03	-0.47***	-0.38***	-0.22**	-0.42***	-0.37***
**PH**	-0.27***	0.36***	-0.03	-0.02	-0.12	-0.22**	0.11	-0.4***	-0.31***	1	0.38***	0.21**	0.21**	-0.03	0.24***	0.28***	0.12	0.3***	0.23**
**TC**	-0.12	0.17*	-0.01	-0.06	-0.17*	-0.05	-0.06	-0.24***	-0.15*	0.38***	1	-0.06	-0.06	-0.23**	0.14	0.28***	0.1	0.1	0.1
**SL**	-0.09	0.09	0.11	-0.05	-0.03	-0.05	-0.03	0.12	0.1	0.21**	-0.06	1	0.66***	0.14	-0.06	-0.1	0.07	-0.03	0
**SPS**	-0.18*	0.18*	0.12	0.07	-0.03	-0.08	0.08	0.08	0.01	0.21**	-0.06	0.66***	1	0.23**	-0.06	-0.04	-0.03	-0.02	-0.05
**GPS**	-0.12	0.09	0.05	0.13	0.08	0.01	-0.18*	0.13	0.03	-0.03	-0.23**	0.14	0.23**	1	0.1	-0.13	0	0.28***	0.17*
**TGW**	-0.29***	0.34***	0.06	-0.02	-0.05	-0.05	0.02	-0.44***	-0.47***	0.24***	0.14	-0.06	-0.06	0.1	1	0.33***	0.59***	0.78***	0.81***
**GY**	-0.26***	0.35***	-0.08	0.06	-0.19**	-0.28***	0.18*	-0.37***	-0.38***	0.28***	0.28***	-0.1	-0.04	-0.13	0.33***	1	0.14	0.32***	0.29***
**GL**	-0.14	0.14	0.21**	-0.14	0.12	-0.02	0.01	-0.17*	-0.22**	0.12	0.1	0.07	-0.03	0	0.59***	0.14	1	0.62***	0.82***
**GB**	-0.31***	0.37***	0.07	0.1	-0.09	-0.17*	0.14	-0.4***	-0.42***	0.3***	0.1	-0.03	-0.02	0.28***	0.78***	0.32***	0.62***	1	0.92***
**GSA**	-0.27***	0.3***	0.13	-0.01	0.02	-0.1	0.04	-0.34***	-0.37***	0.23**	0.1	0	-0.05	0.17*	0.81***	0.29***	0.82***	0.92***	1

Phi2, PS II quantum yield; NPQ, non-photochemical quenching; RC, relative chlorophyll; CTD, canopy temperature depression; PS1, active PS I center; LT, leaf thickness; LA, leaf angle; DTH, days to heading; DTM, days to maturity; PH, plant height; TC, tiller count; SL, spike length; SPS, spikelets per spike; GPS, grains per spike; TGW, thousand-grain weight; GY, grain yield; GL, grain length; GB, grain breadth; GSA, grain surface area

* P ≤ 0.05; ** P ≤ 0.01; *** P ≤ 0.001

SUPPLEMENTARY FIGURES

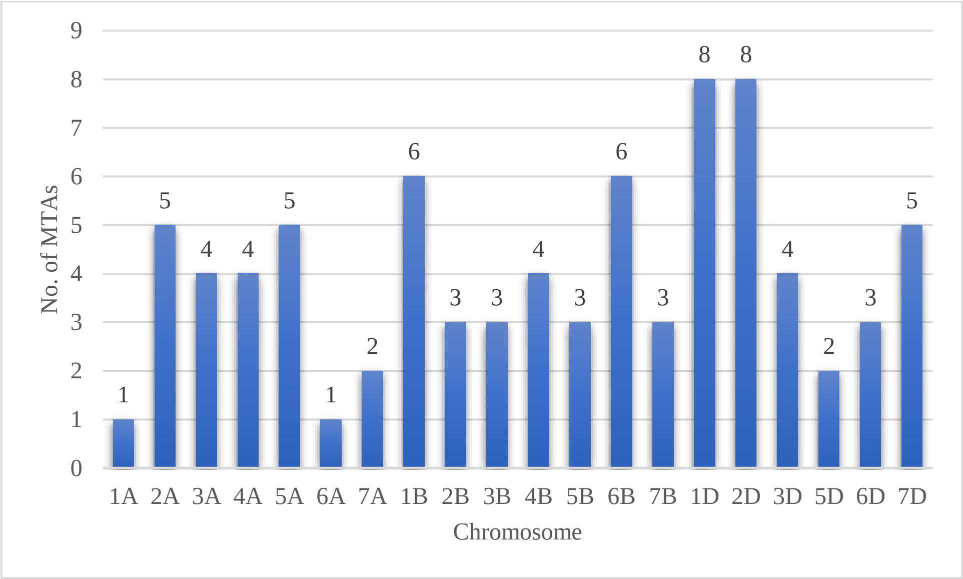

Supplementary Fig S1 Chromosome-wise distribution of the identified MTAs through BLINK in GAPIT

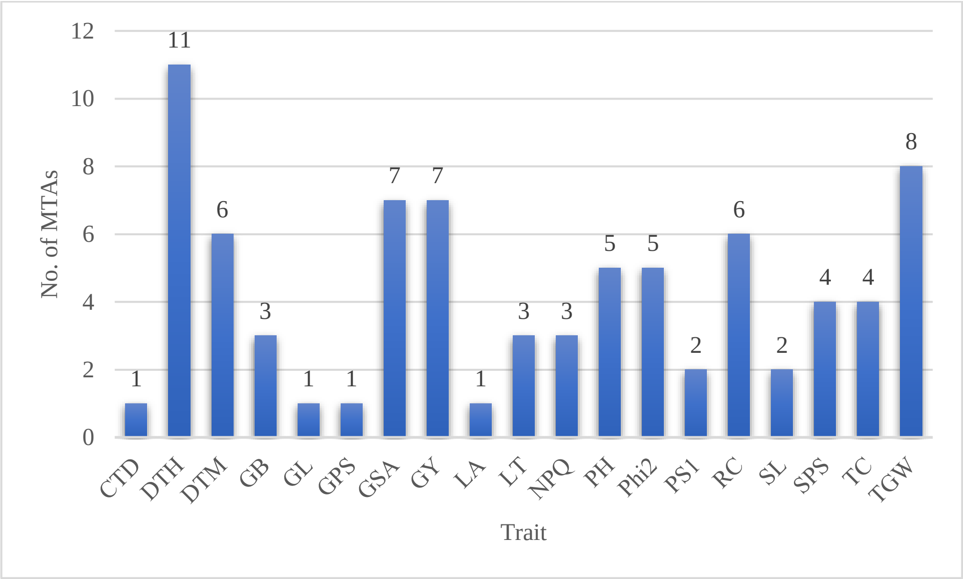

Supplementary Fig S2 Trait-wise distribution of the identified MTAs through BLINK in GAPIT

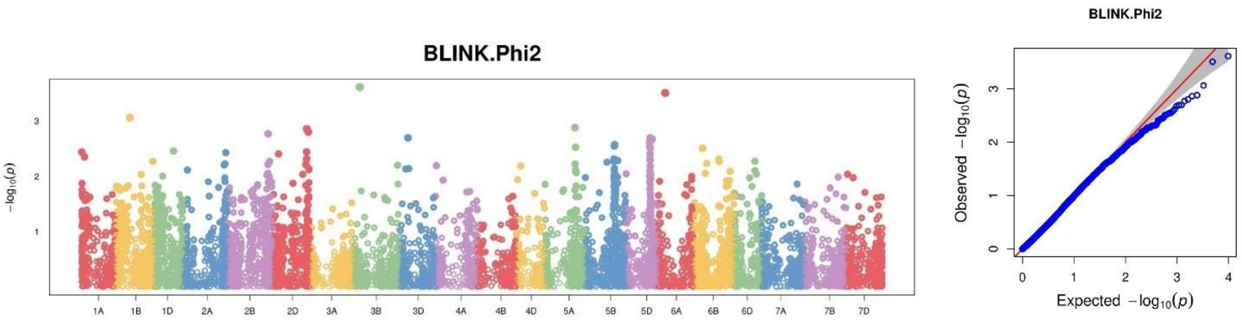

(a)

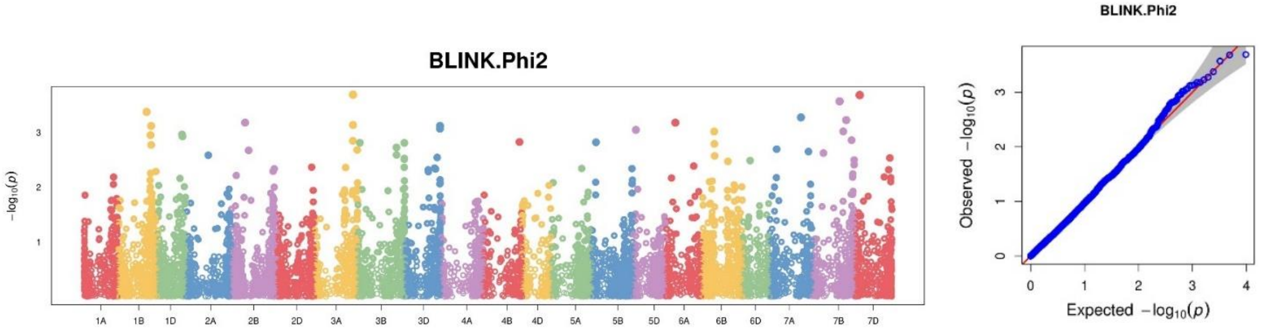

(b)

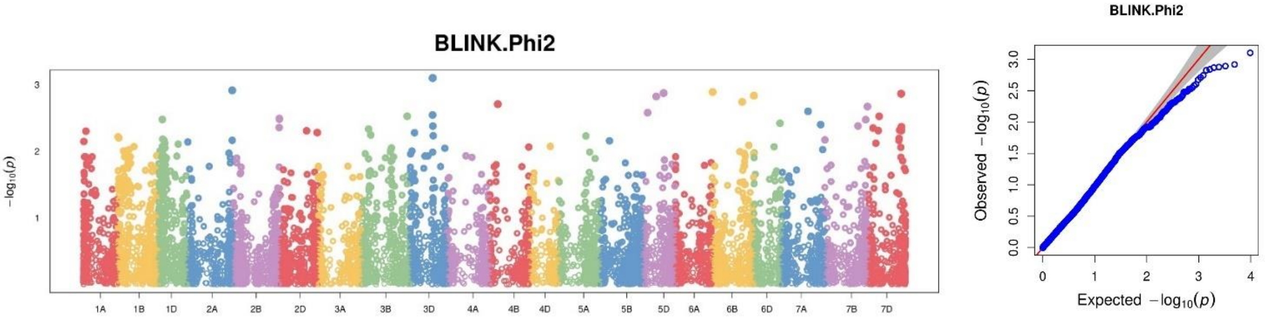

(c)

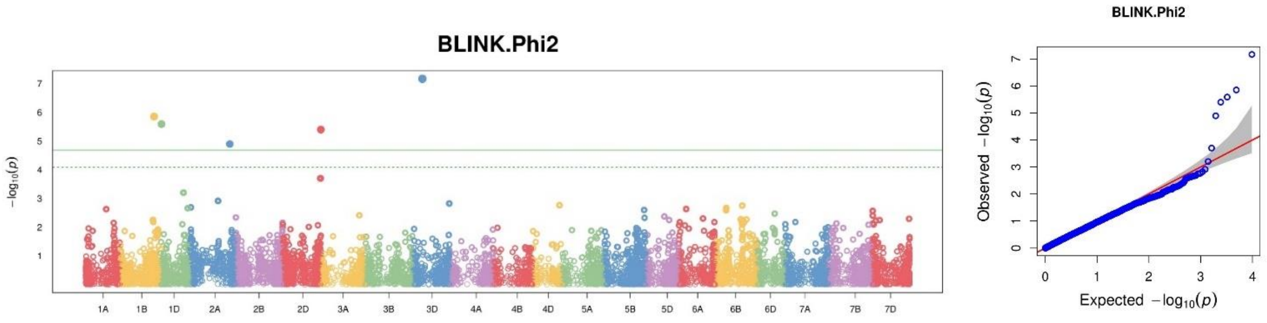

(d)

Fig S3 Manhattan plots and QQ plots and QQ plots for Phi2 under (a) CTTS, (b) CATS, (c) CTLS, and (d) CALS

Phi2, PS II quantum yield; CTTS, Conventional tillage timely sown; CATS, Conservation agriculture timely sown; CTLS, Conventional tillage late sown; CALS, Conservation agriculture late sown

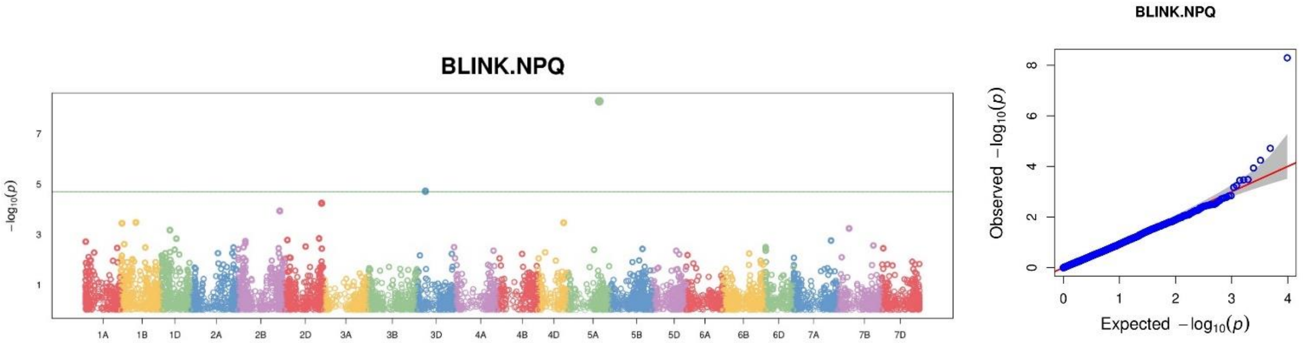

(a)

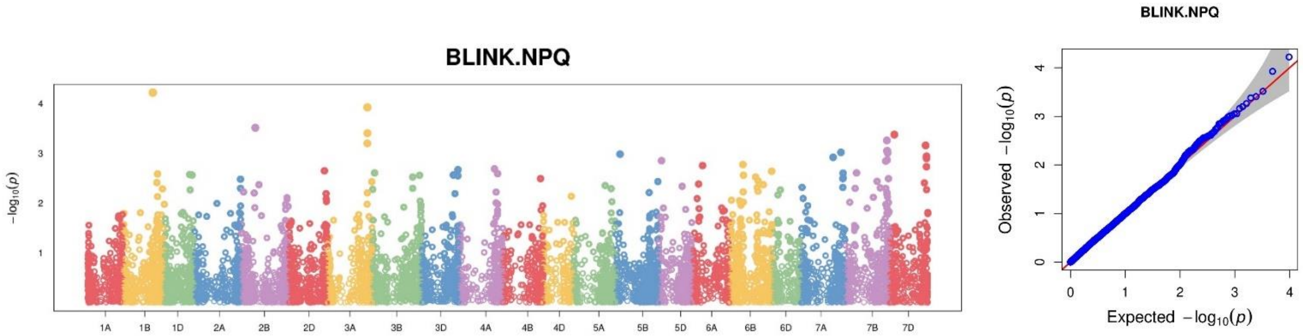

(b)

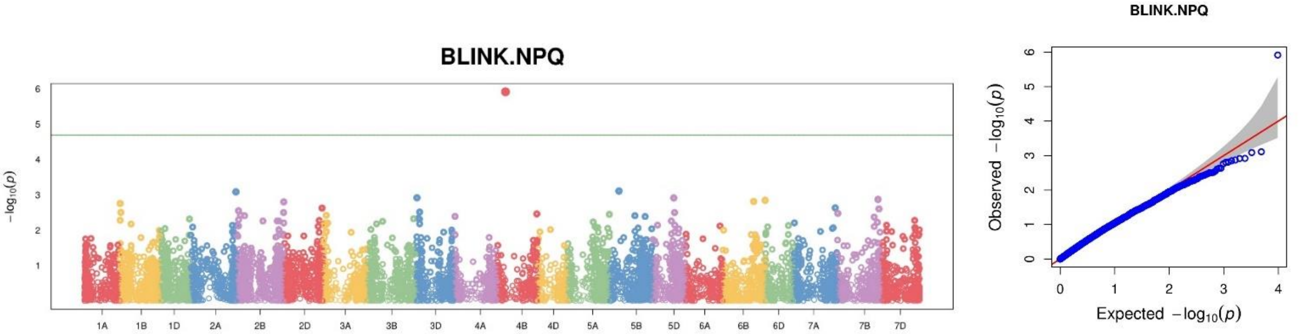

(c)

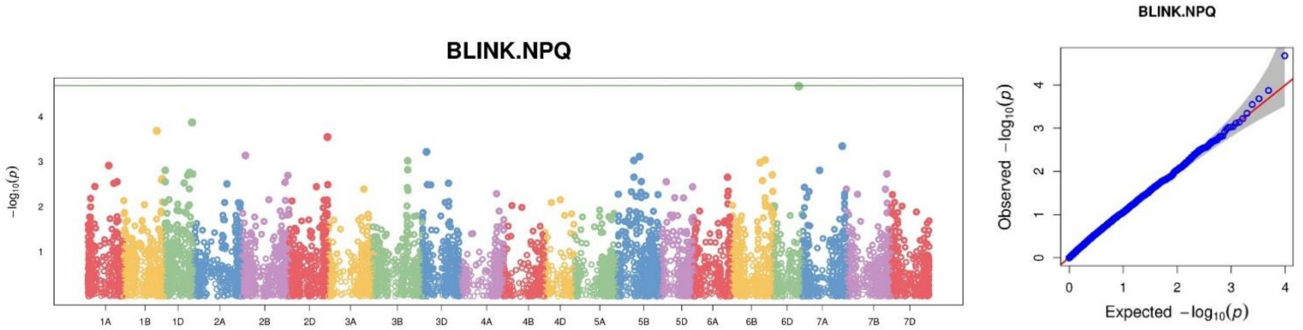

(d)

Fig S4 Manhattan plots and QQ plots and QQ plots for NPQ under (a) CTTS, (b) CATS, (c) CTLS, and (d) CALS NPQ, non-photochemical quenching; CTTS, Conventional tillage timely sown; CATS, Conservation agriculture timely sown; CTLS, Conventional tillage late sown; CALS, Conservation agriculture late sown.

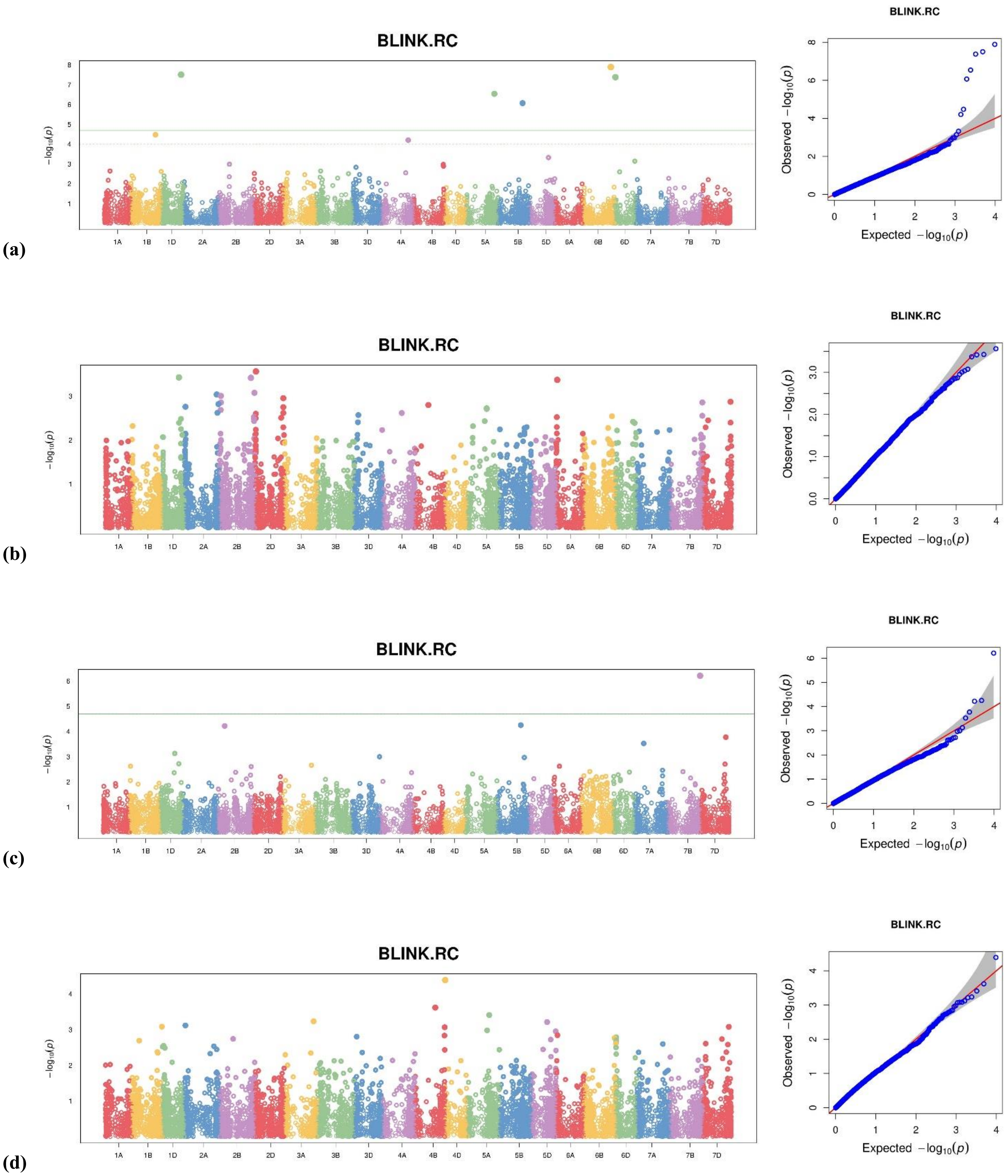

Fig S5 Manhattan plots and QQ plots and QQ plots for RC under (a) CTTS, (b) CATS, (c) CTLS, and (d) CALS

RC, relative chlorophyll; CTTS, Conventional tillage timely sown; CATS, Conservation agriculture timely sown; CTLS, Conventional tillage late sown; CALS, Conservation agriculture late sown.

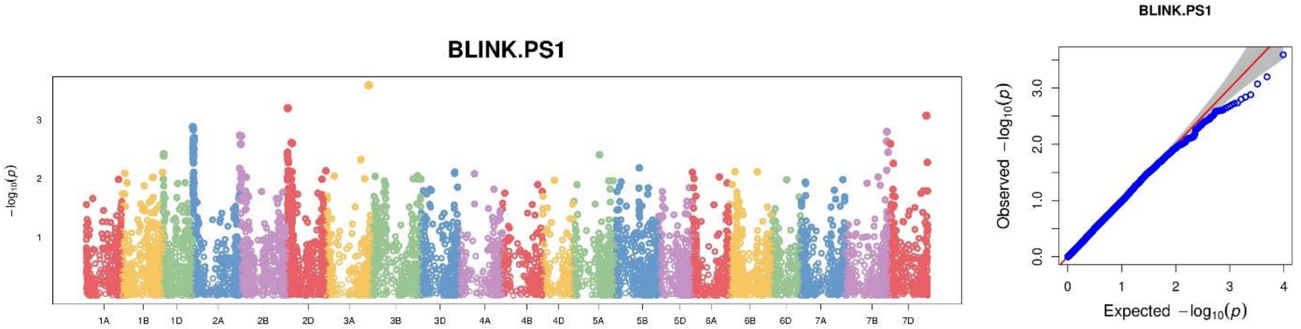

(a)

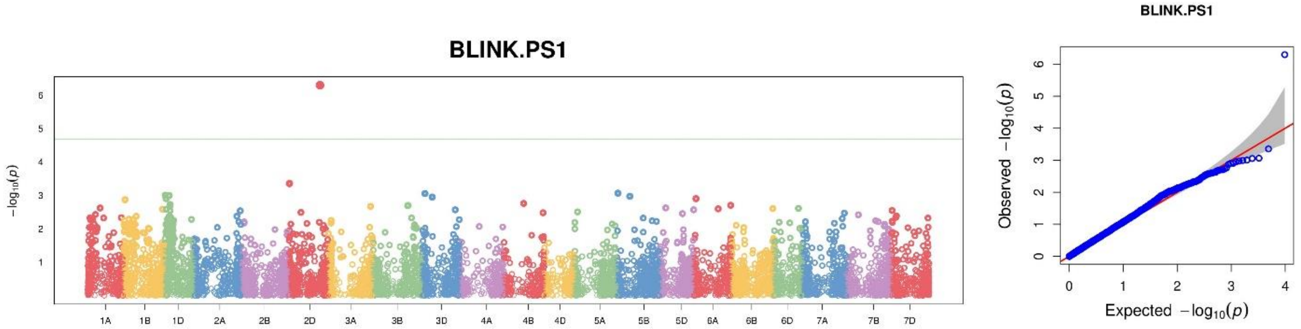

(b)

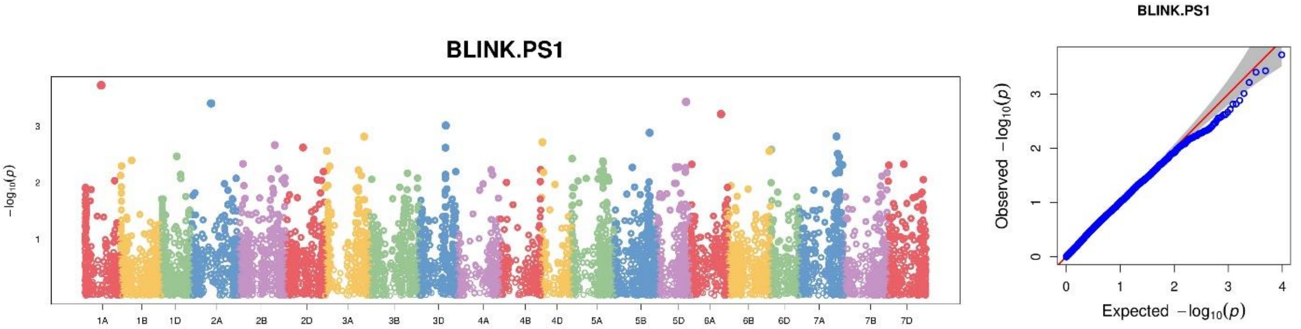

(c)

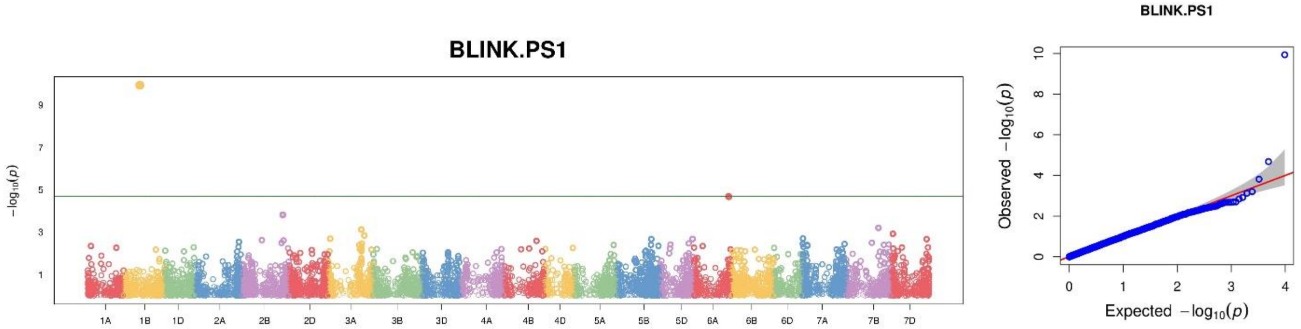

(d)

Fig S6 Manhattan plots and QQ plots and QQ plots for PS1 under (a) CTTS, (b) CATS, (c) CTLS, and (d) CALS PS1, active PS I center; CTTS, Conventional tillage timely sown; CATS, Conservation agriculture timely sown; CTLS, Conventional tillage late sown; CALS, Conservation agriculture late sown.

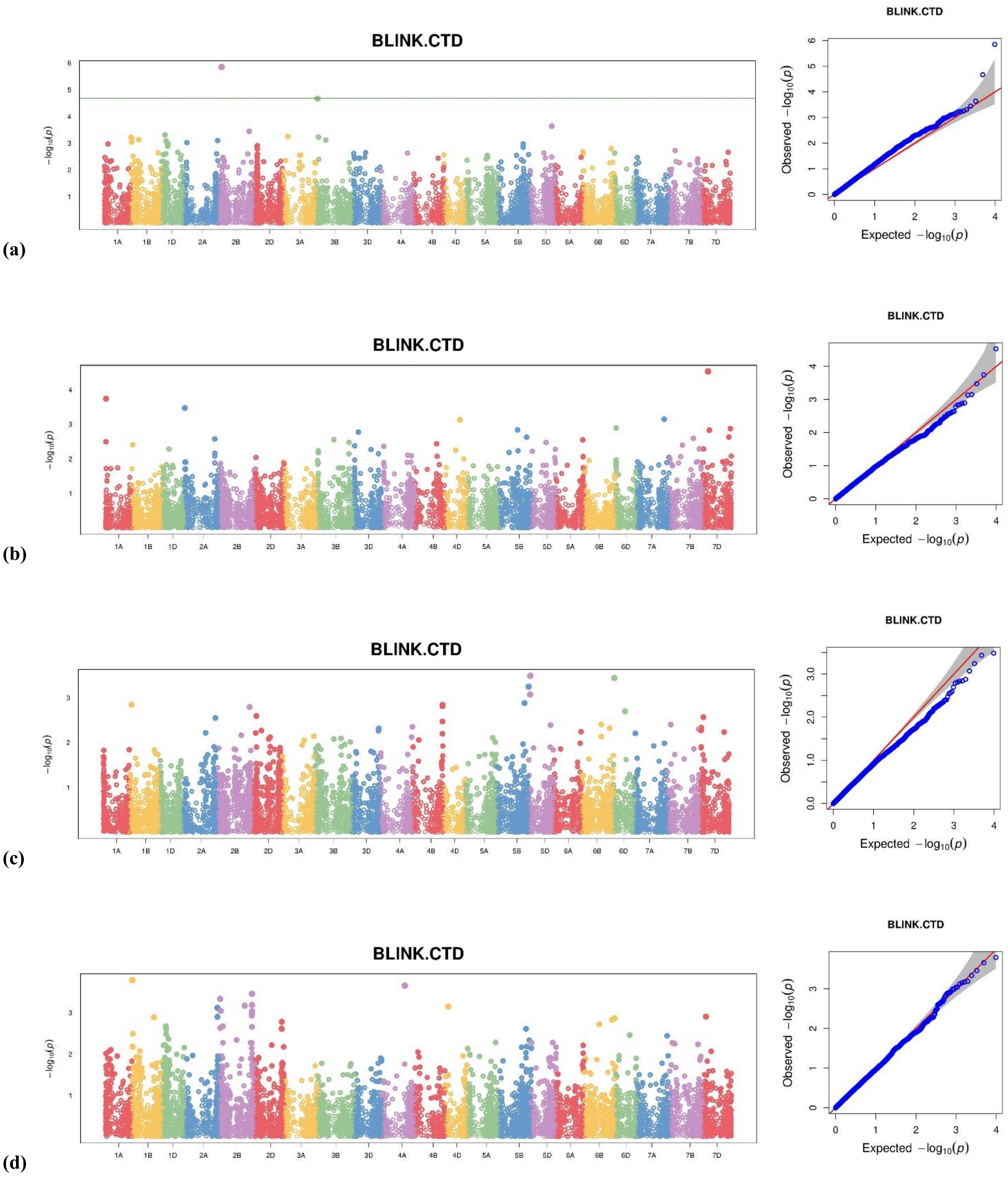

Fig S7 Manhattan plots and QQ plots and QQ plots for CTD under (a) CTTS, (b) CATS, (c) CTLS, and (d) CALS

CTD, canopy temperature depression; CTTS, Conventional tillage timely sown; CATS, Conservation agriculture timely sown; CTLS, Conventional tillage late sown; CALS, Conservation agriculture late sown.

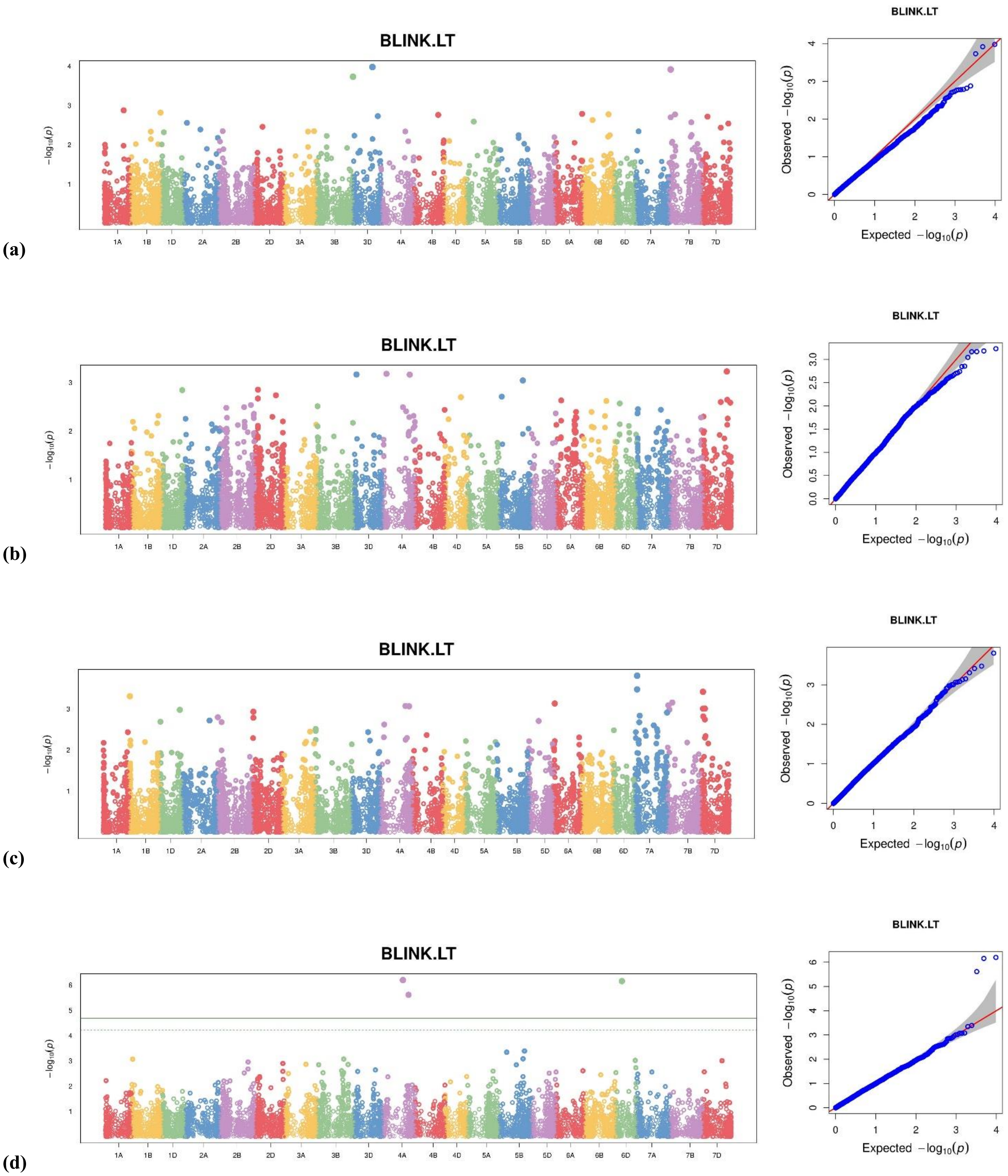

Fig S8 Manhattan plots and QQ plots and QQ plots for LT under (a) CTTS, (b) CATS, (c) CTLS, and (d) CALS
LT, leaf thickness; CTTS, Conventional tillage timely sown; CATS, Conservation agriculture timely sown; CTLS, Conventional tillage late sown; CALS, Conservation agriculture late sown.

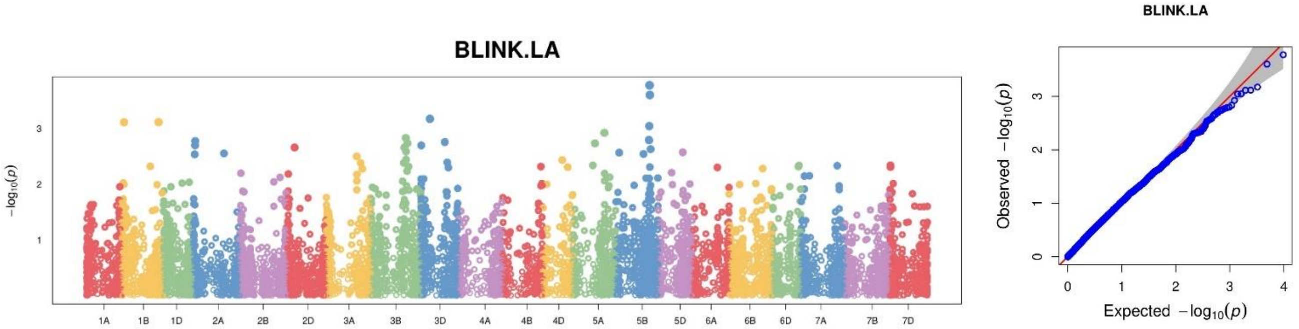

(a)

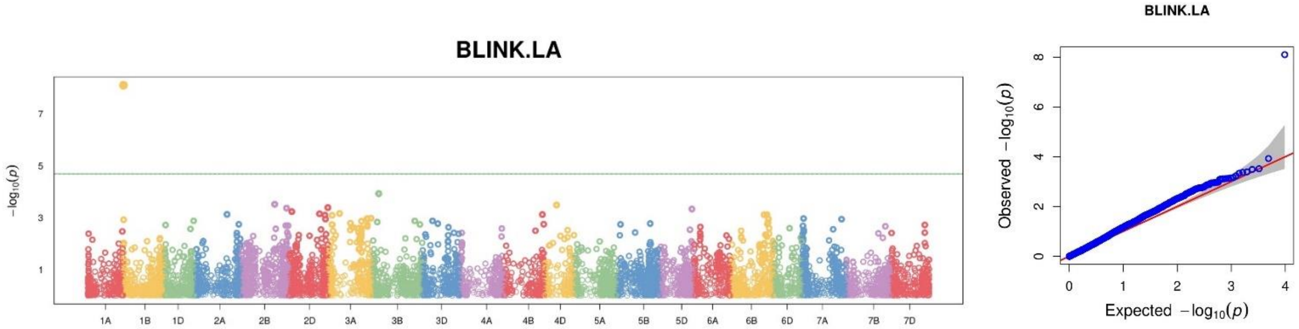

(b)

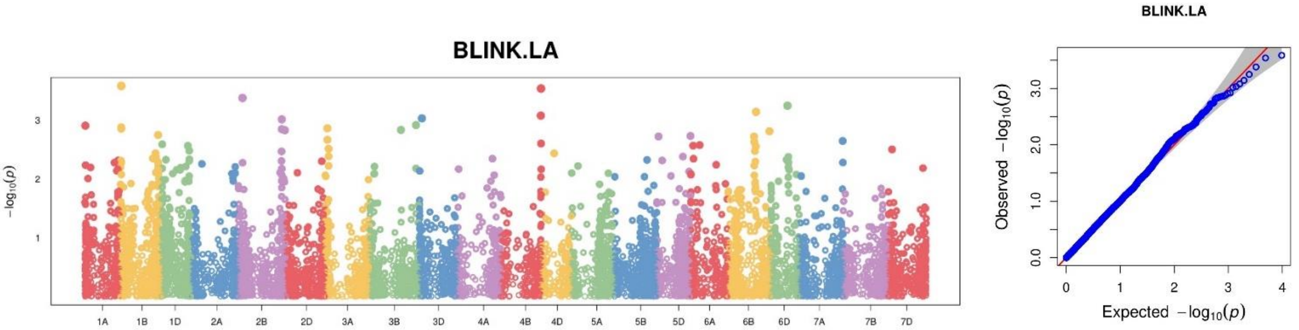

(c)

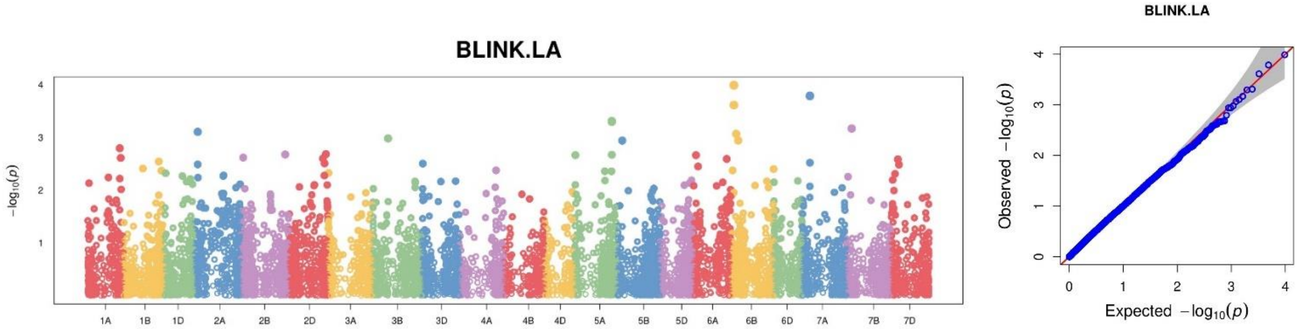

(d)

Fig S9 Manhattan plots and QQ plots and QQ plots for LA under (a) CTTS, (b) CATS, (c) CTLS, and (d) CALS

LA, leaf angle; CTTS, Conventional tillage timely sown; CATS, Conservation agriculture timely sown; CTLS, Conventional tillage late sown; CALS, Conservation agriculture late sown.

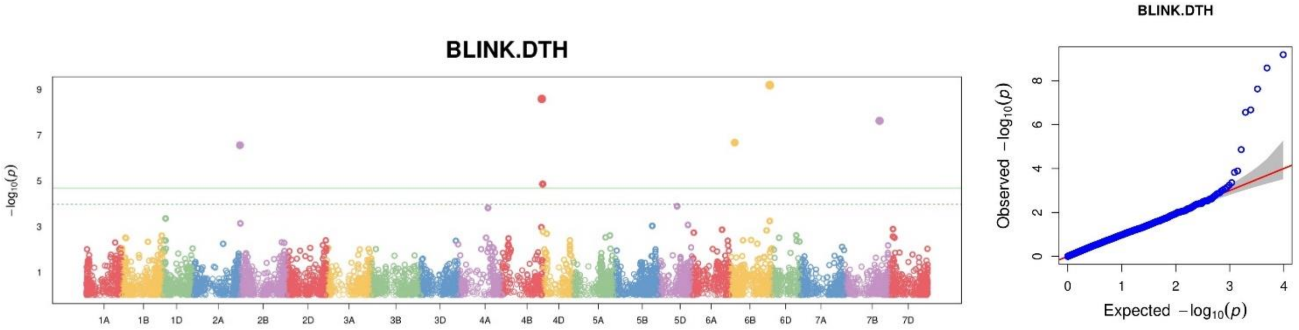

(a)

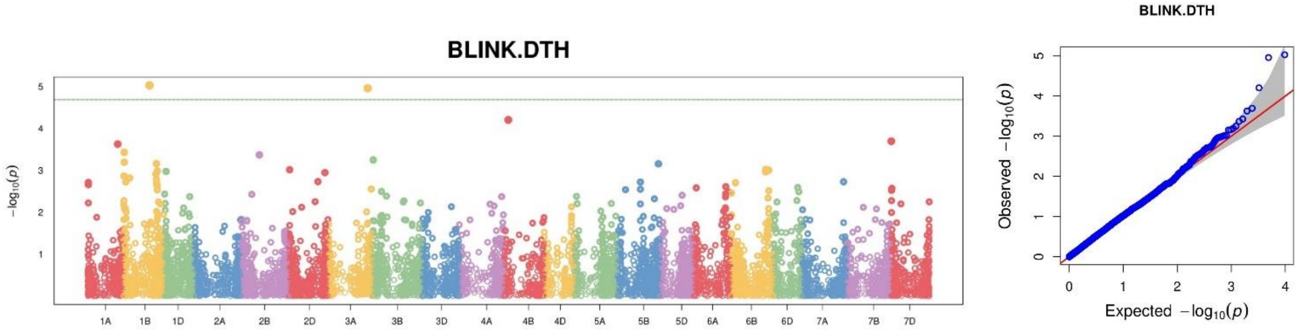

(b)

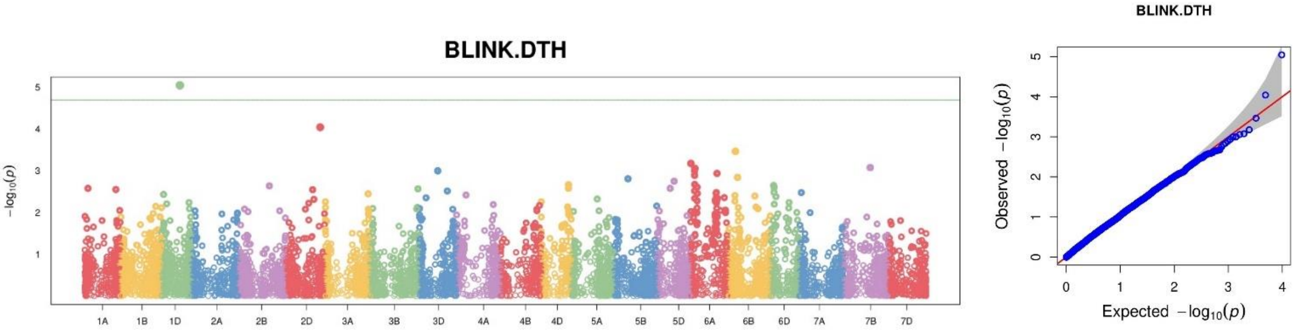

(c)

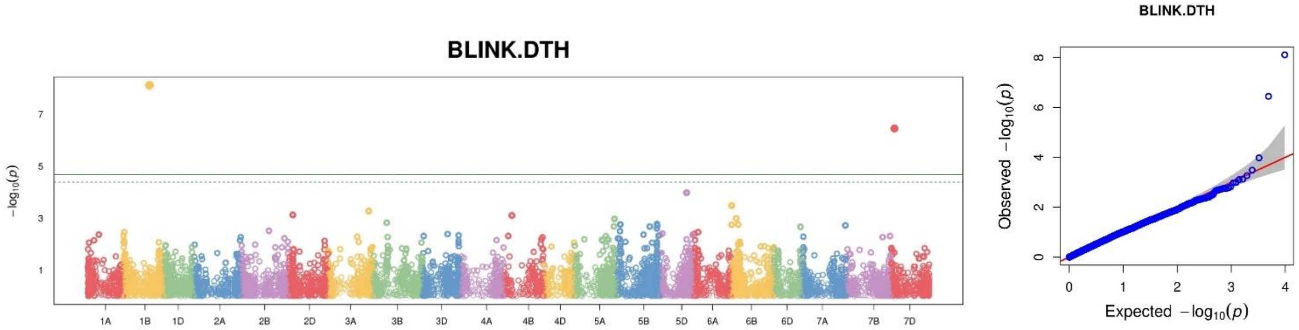

(d)

Fig S10 Manhattan plots and QQ plots and QQ plots for DTH under (a) CTTS, (b) CATS, (c) CTLS, and (d) CALS DTH, days to heading; CTTS, Conventional tillage timely sown; CATS, Conservation agriculture timely sown; CTLS, Conventional tillage late sown; CALS, Conservation agriculture late sown.

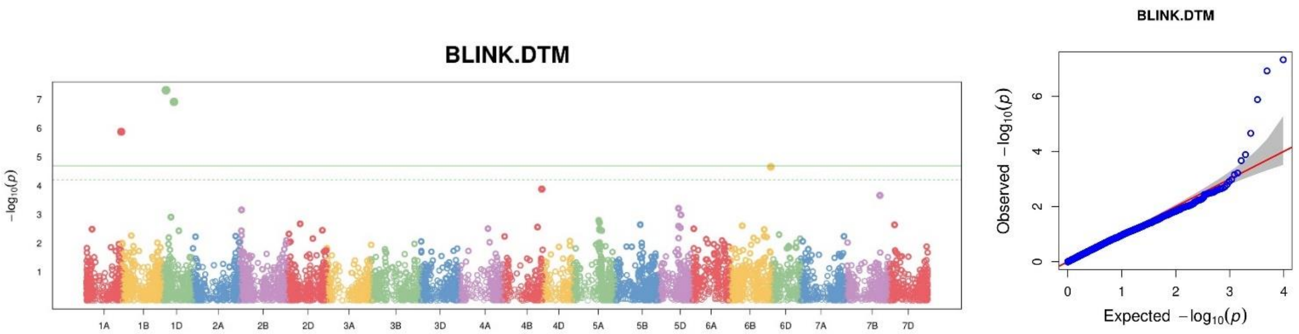

(a)

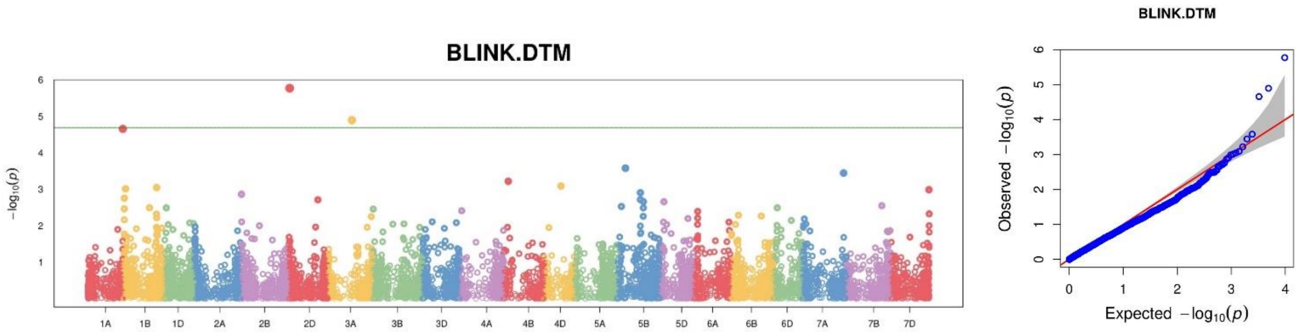

(b)

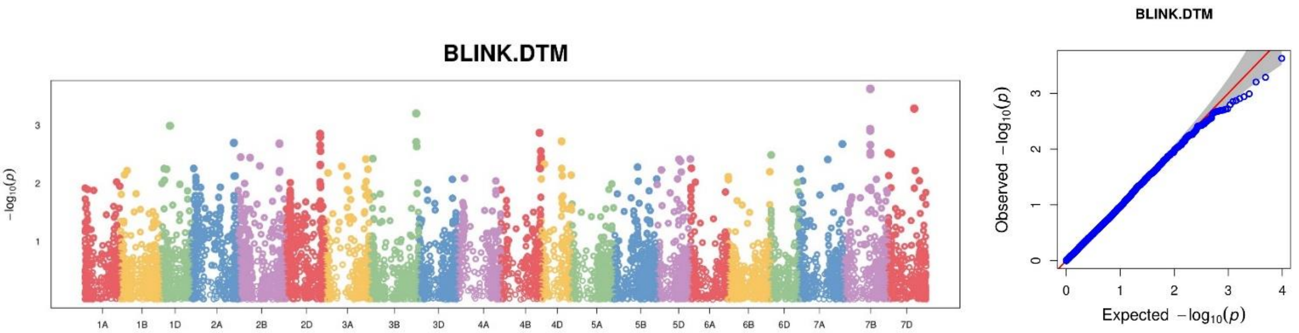

(c)

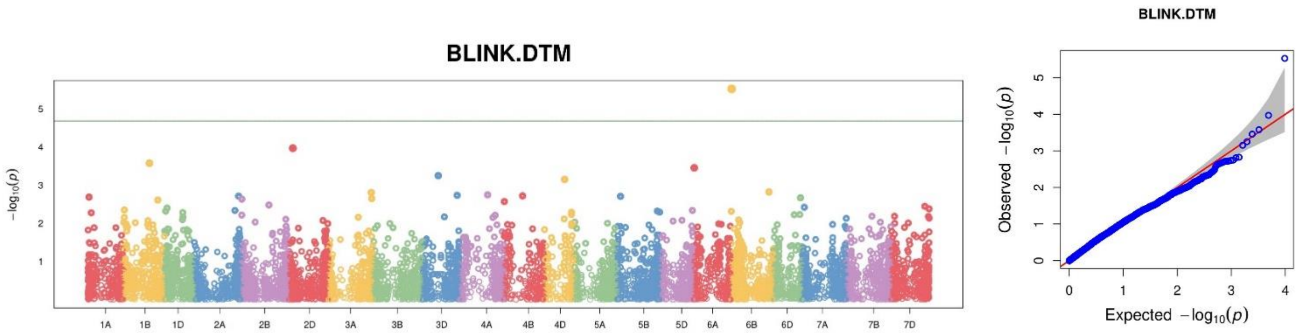

(d)

Fig S11 Manhattan plots and QQ plots and QQ plots for DTM under (a) CTTS, (b) CATS, (c) CTLS, and (d) CALS DTM, days to maturity; CTTS, Conventional tillage timely sown; CATS, Conservation agriculture timely sown; CTLS, Conventional tillage late sown; CALS, Conservation agriculture late sown.

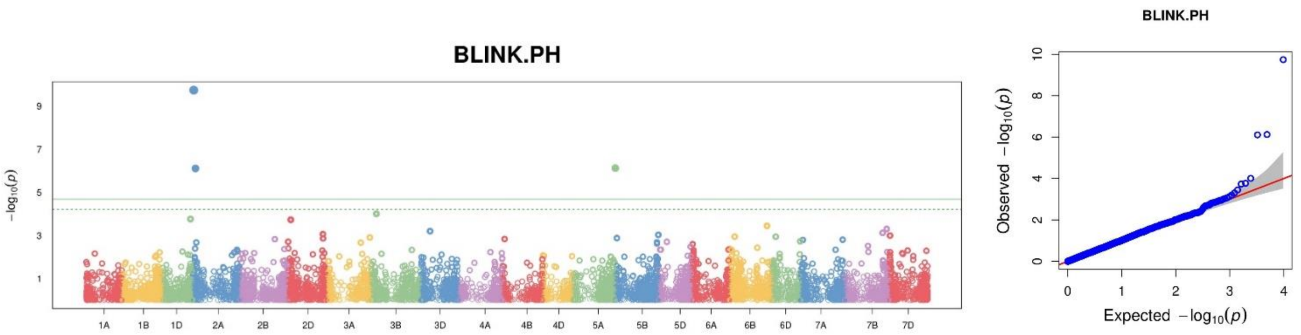

(a)

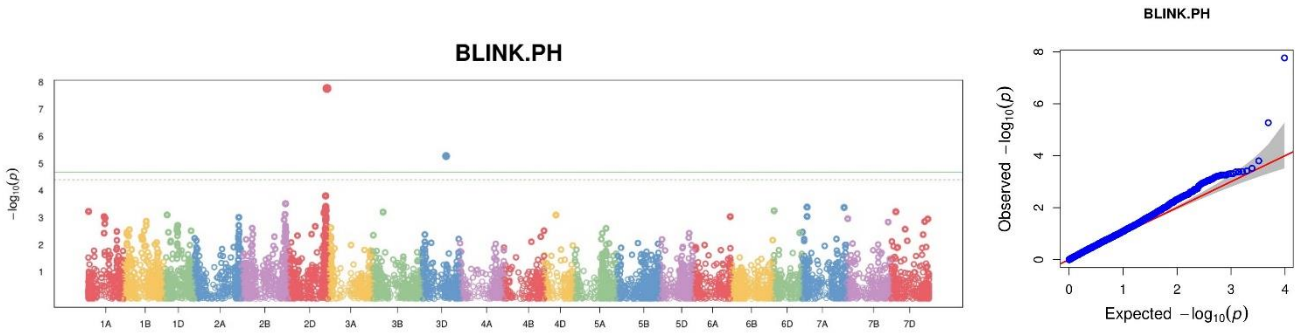

(b)

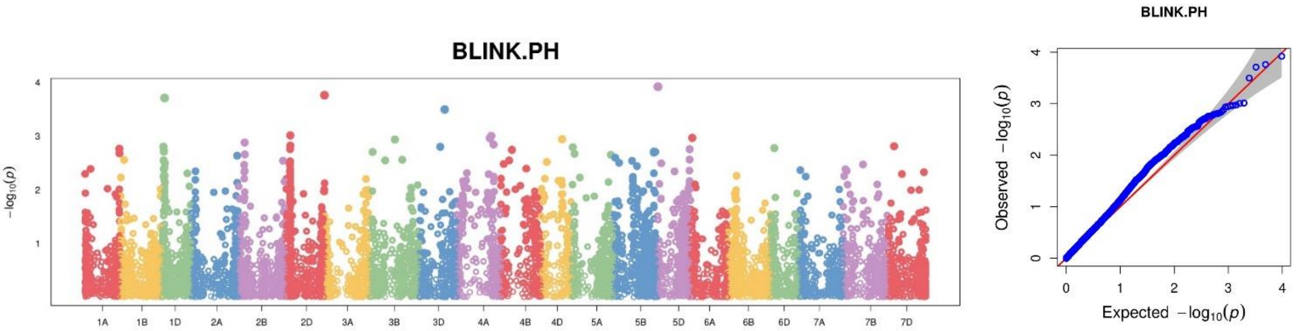

(c)

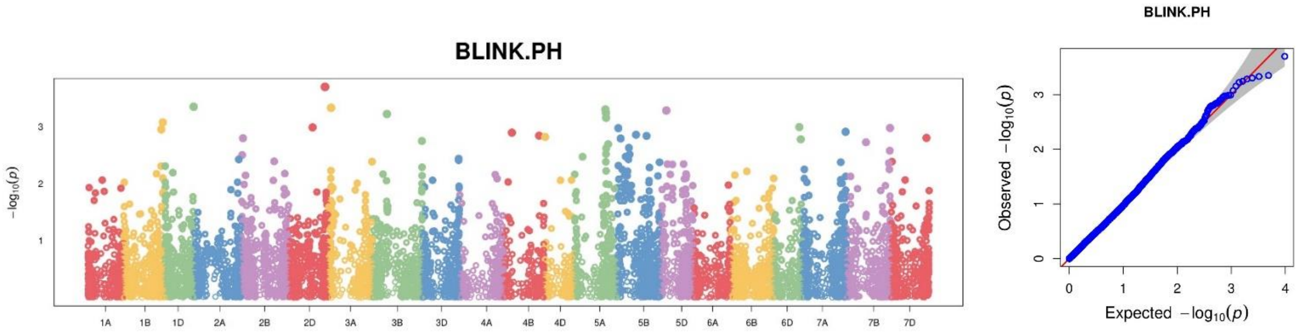

(d)

Fig S12 Manhattan plots and QQ plots and QQ plots for PH under (a) CTTS, (b) CATS, (c) CTLS, and (d) CALS

PH, plant height; CTTS, Conventional tillage timely sown; CATS, Conservation agriculture timely sown; CTLS, Conventional tillage late sown; CALS, Conservation agriculture late sown.

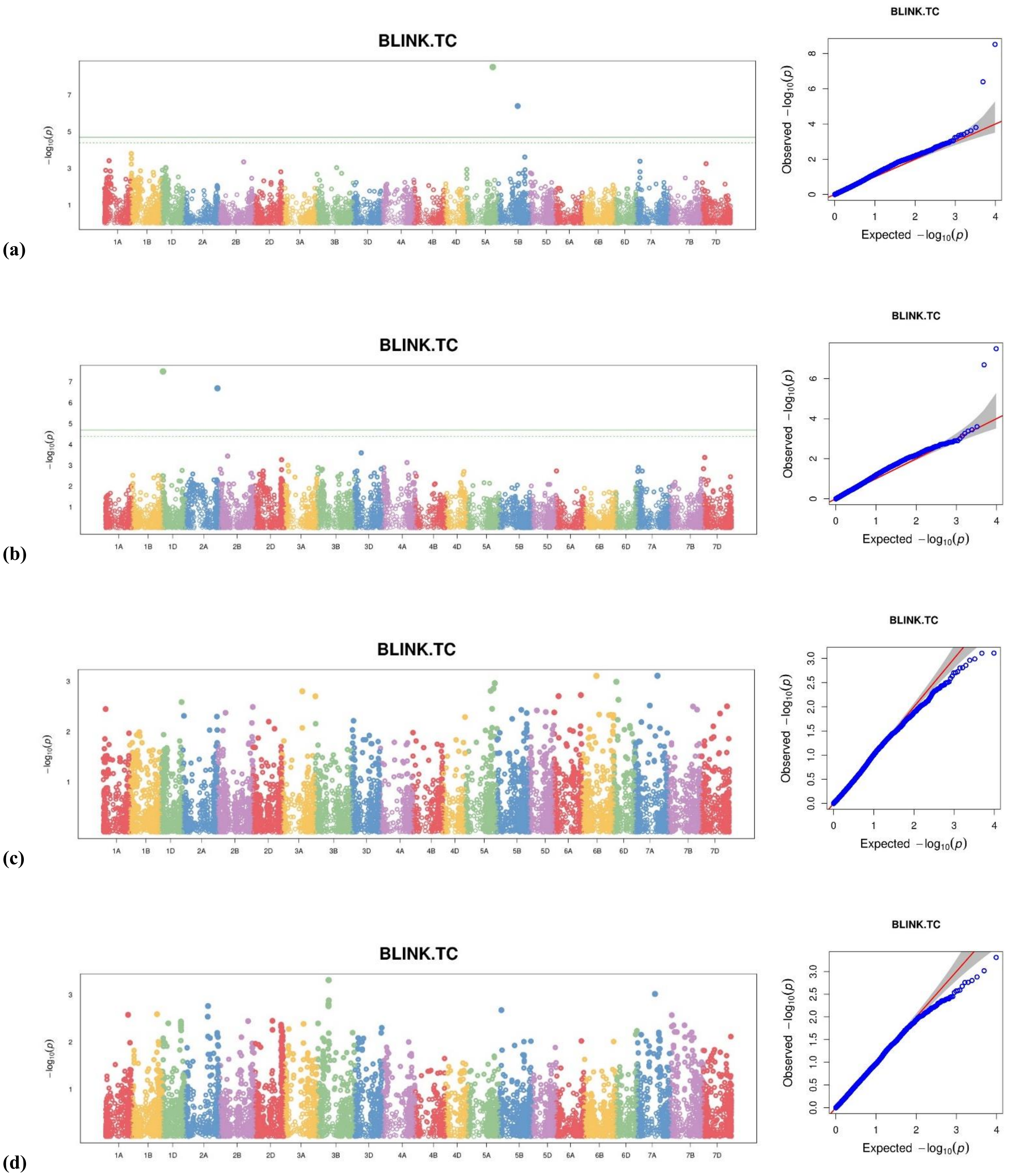

Fig S13 Manhattan plots and QQ plots and QQ plots for TC under (a) CTTS, (b) CATS, (c) CTLS, and (d) CALS

TC, tiller count; CTTS, Conventional tillage timely sown; CATS, Conservation agriculture timely sown; CTLS, Conventional tillage late sown; CALS, Conservation agriculture late sown.

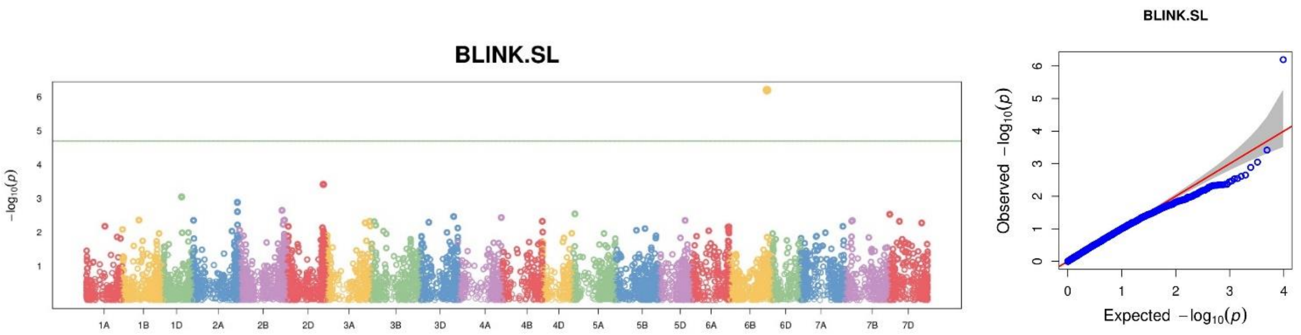

(a)

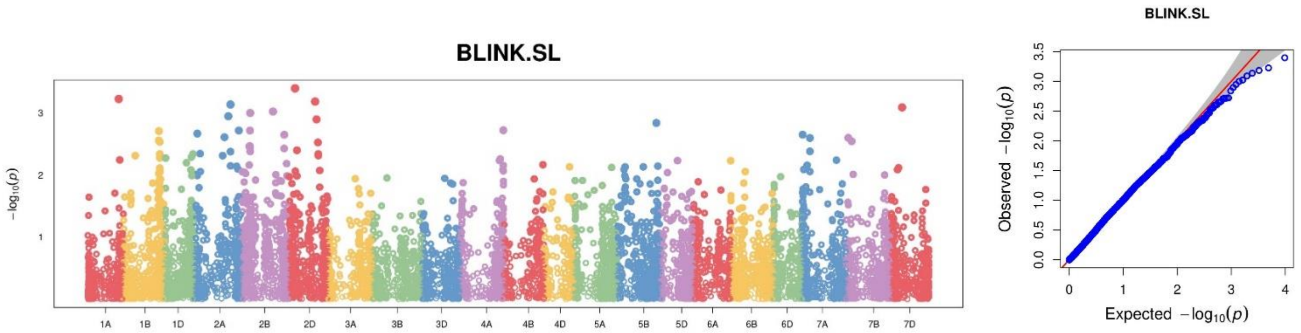

(b)

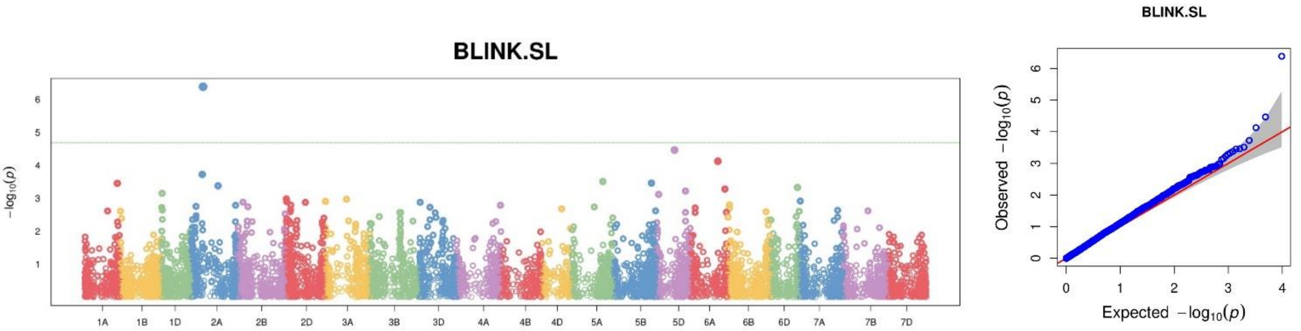

(c)

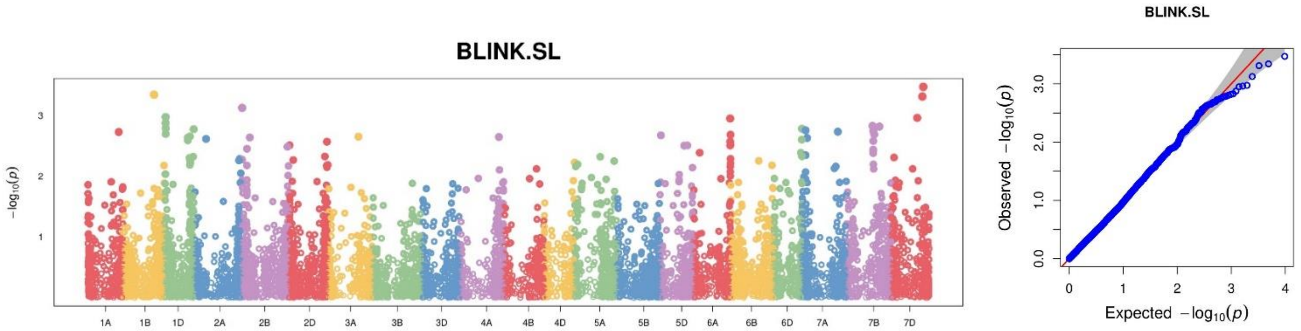

(d)

Fig S14 Manhattan plots and QQ plots and QQ plots for SL under (a) CTTS, (b) CATS, (c) CTLS, and (d) CALS SL, spike length; CTTS, Conventional tillage timely sown; CATS, Conservation agriculture timely sown; CTLS, Conventional tillage late sown; CALS, Conservation agriculture late sown.

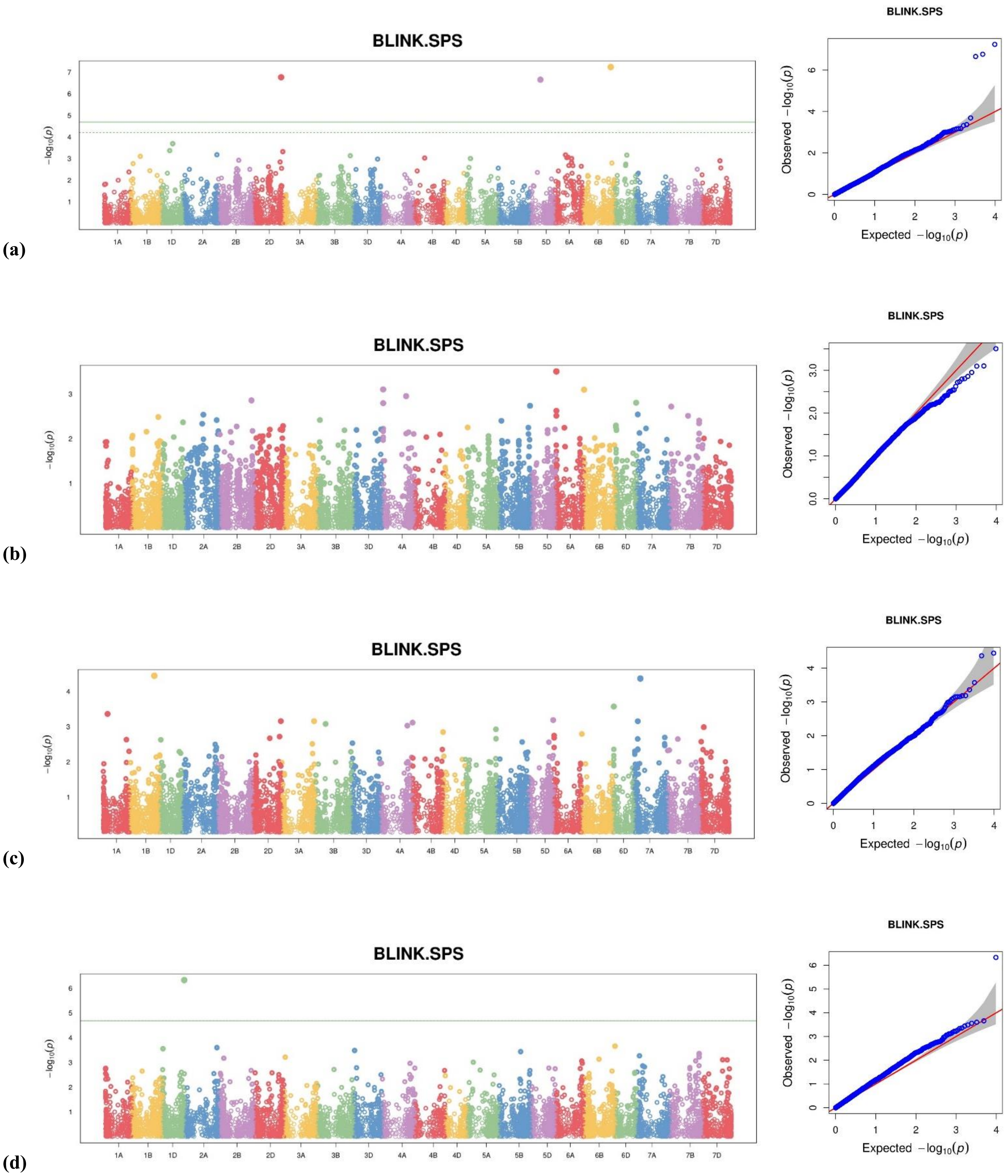

Fig S15 Manhattan plots and QQ plots for SPS under (a) CTTS, (b) CATS, (c) CTLS, and (d) CALS

SPS, spikelets per spike; CTTS, Conventional tillage timely sown; CATS, Conservation agriculture timely sown; CTLS, Conventional tillage late sown; CALS, Conservation agriculture late sown.

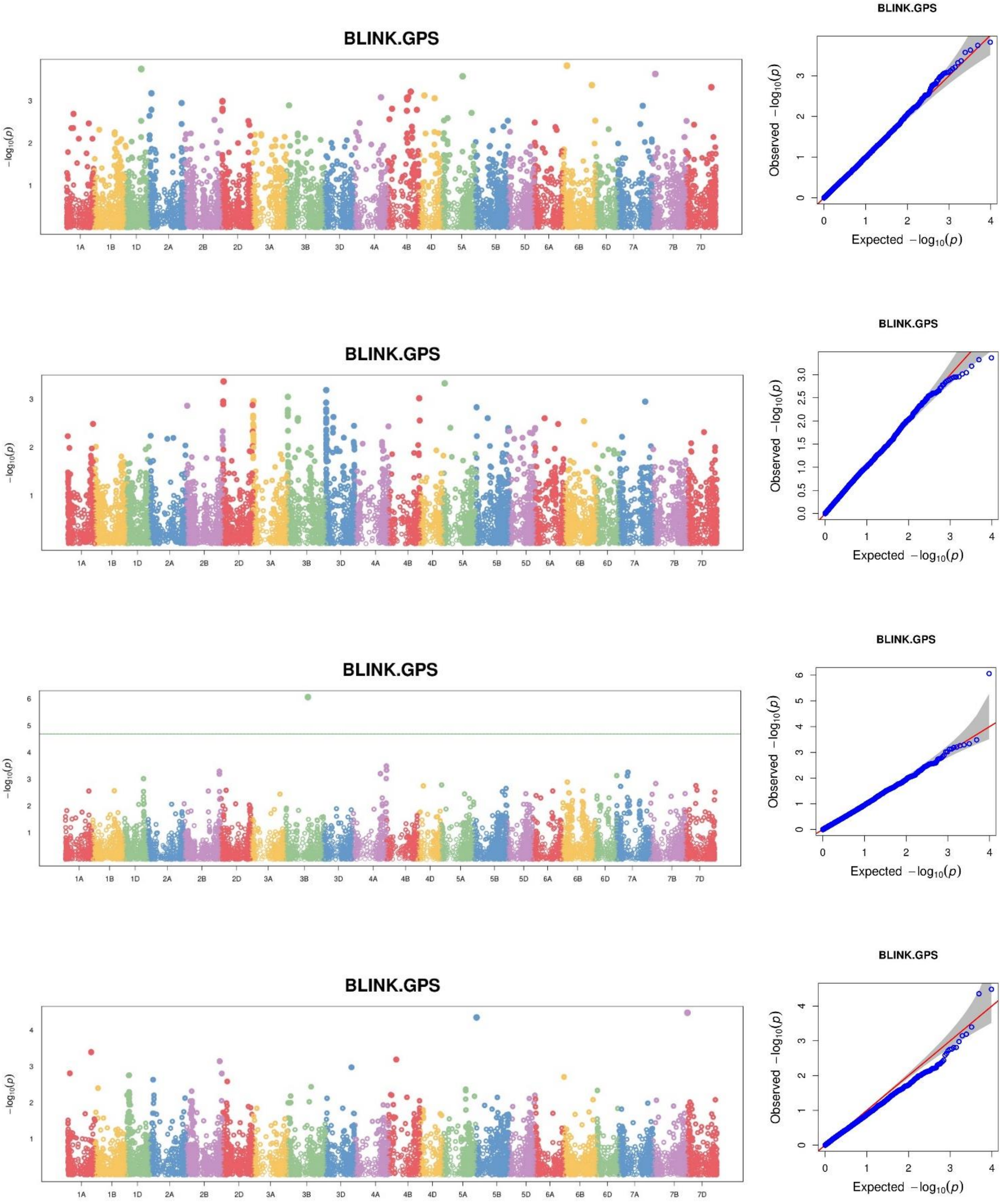

(a)

(b)

(c)

(d)

Fig S16 Manhattan plots and QQ plots for GPS under (a) CTTS, (b) CATS, (c) CTLS, and (d) CALS
GPS, grains per spike; CTTS, Conventional tillage timely sown; CATS, Conservation agriculture timely sown; CTLS, Conventional tillage late sown; CALS, Conservation agriculture late sown.

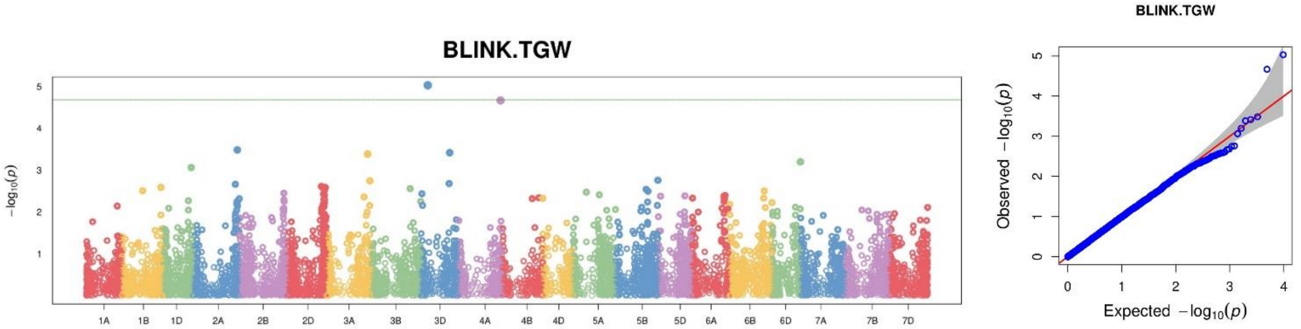

(a)

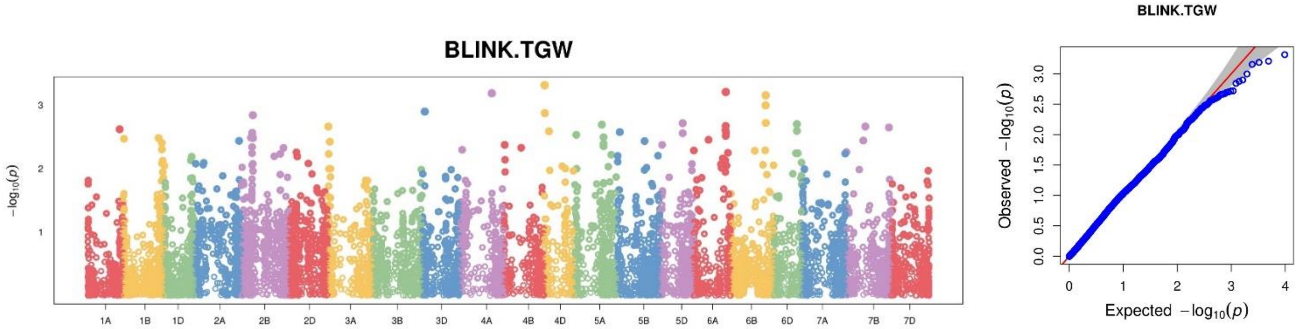

(b)

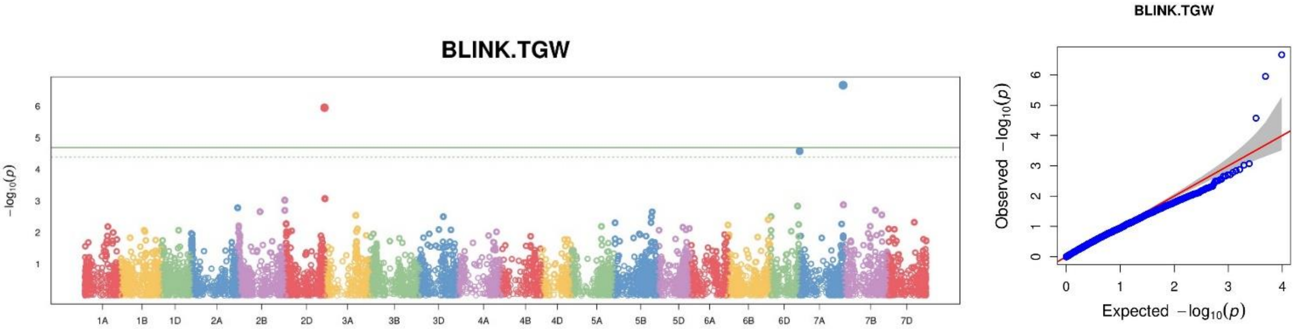

(c)

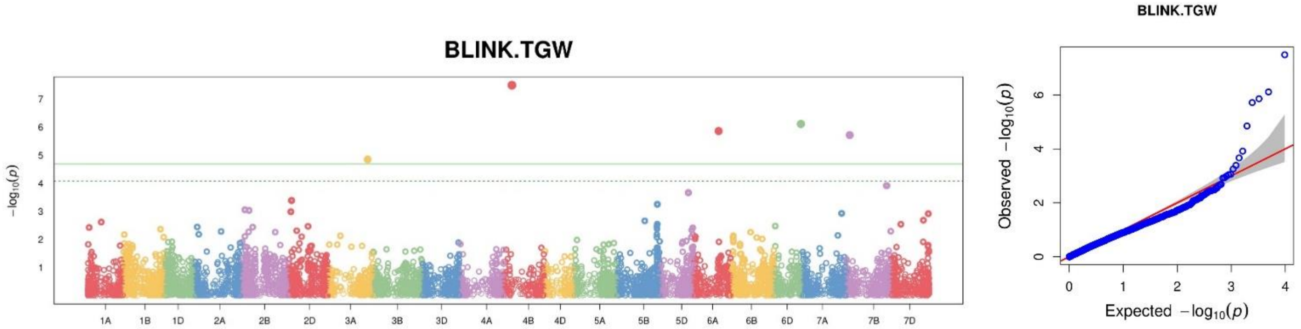

(d)

Fig S17 Manhattan plots and QQ plots for TGW under (a) CTTS, (b) CATS, (c) CTLS, and (d) CALS

TGW, thousand-grain weight; CTTS, Conventional tillage timely sown; CATS, Conservation agriculture timely sown; CTLS, Conventional tillage late sown; CALS, Conservation agriculture late sown.

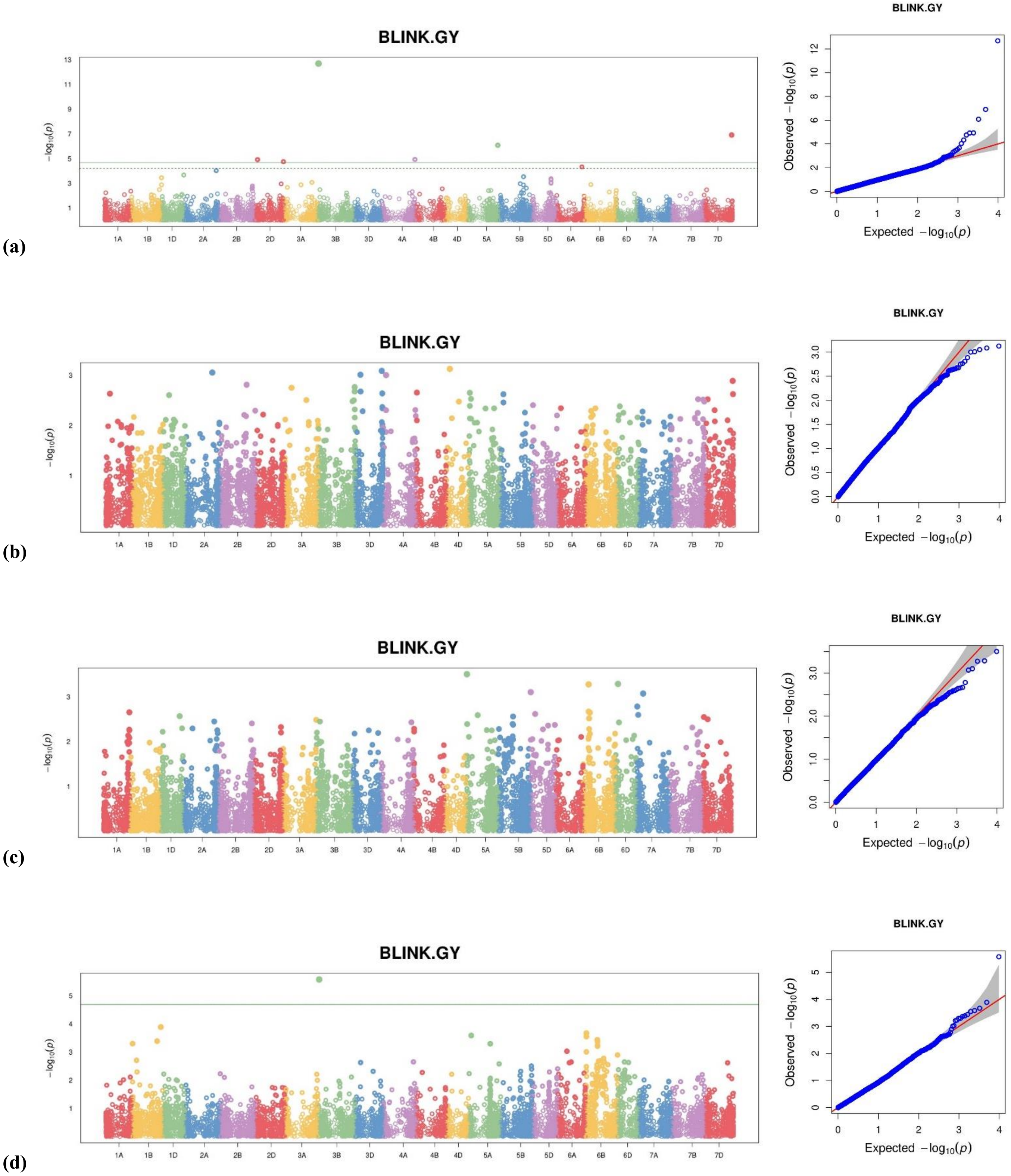

Fig S18 Manhattan plots and QQ plots for GY under (a) CTTS, (b) CATS, (c) CTLS, and (d) CALS

GY, grain yield; CTTS, Conventional tillage timely sown; CATS, Conservation agriculture timely sown; CTLS, Conventional tillage late sown; CALS, Conservation agriculture late sown.

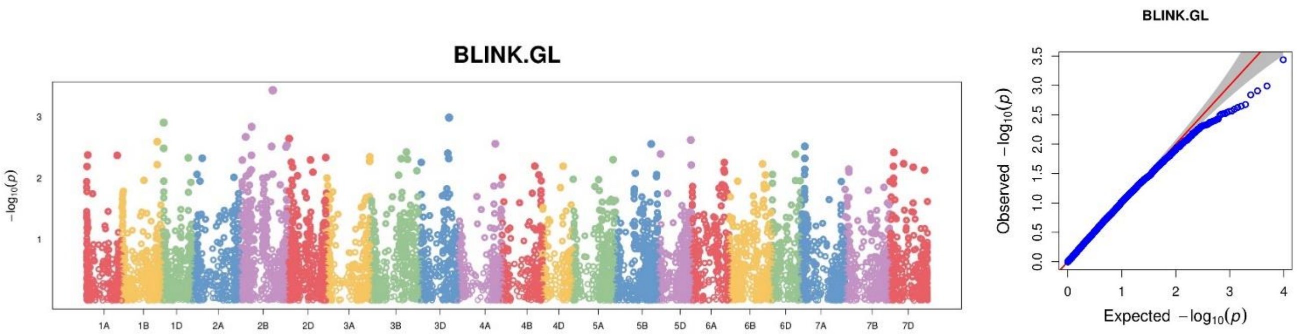

(a)

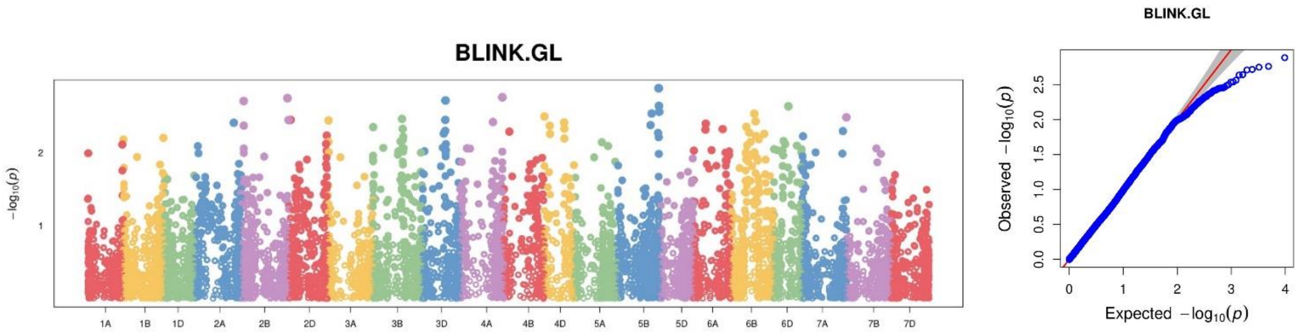

(b)

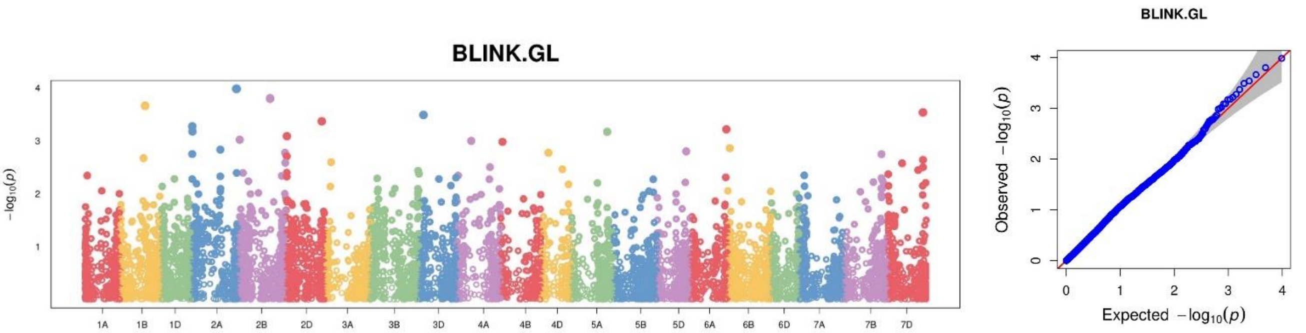

(c)

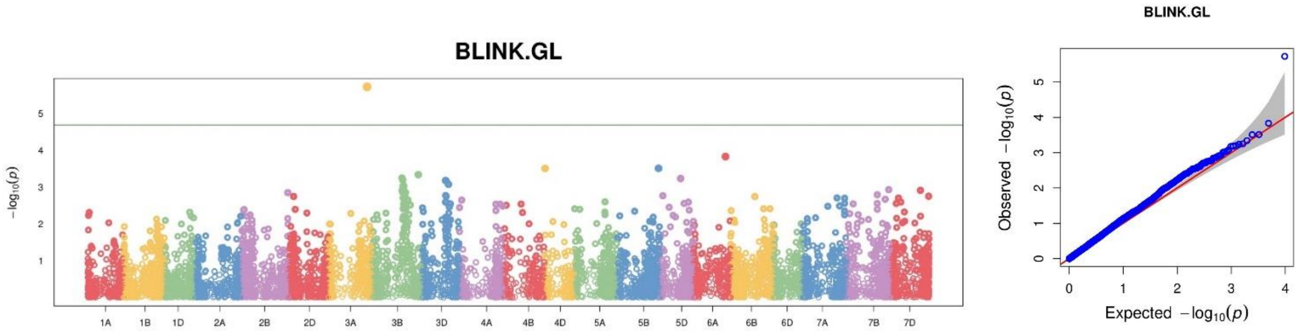

(d)

Fig S19 Manhattan plots and QQ plots for GL under (a) CTTS, (b) CATS, (c) CTLS, and (d) CALS

GL, grain length; CTTS, Conventional tillage timely sown; CATS, Conservation agriculture timely sown; CTLS, Conventional tillage late sown; CALS, Conservation agriculture late sown.

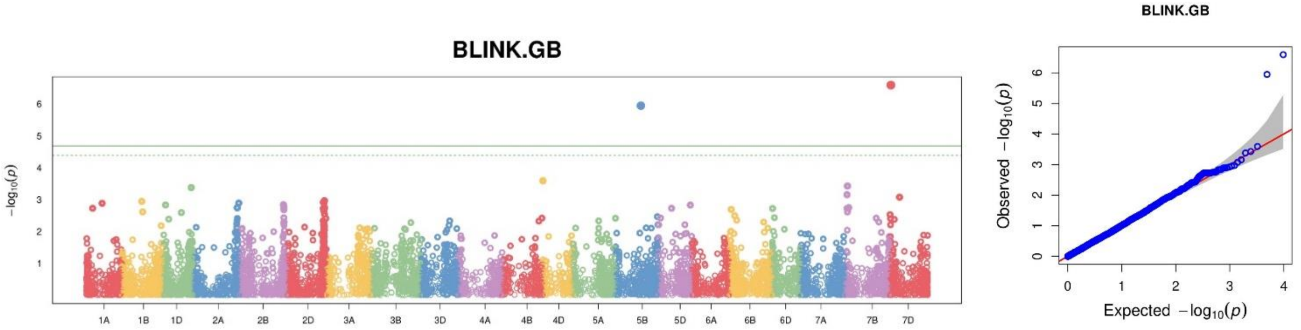

(a)

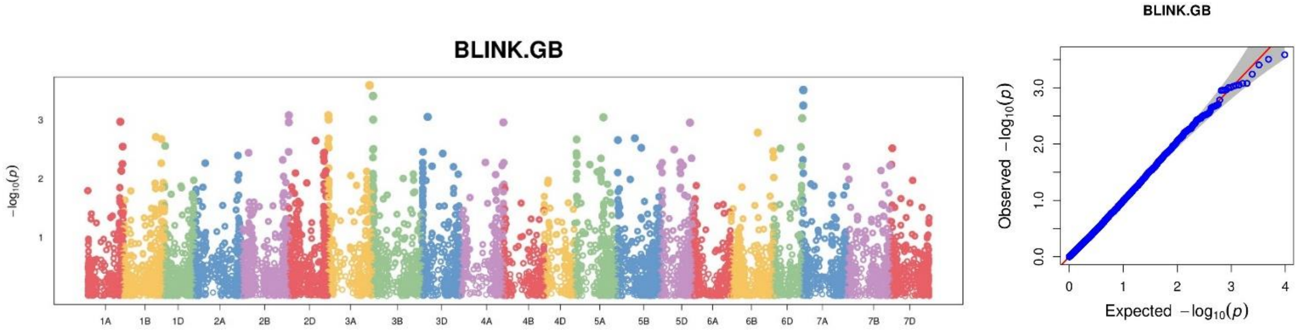

(b)

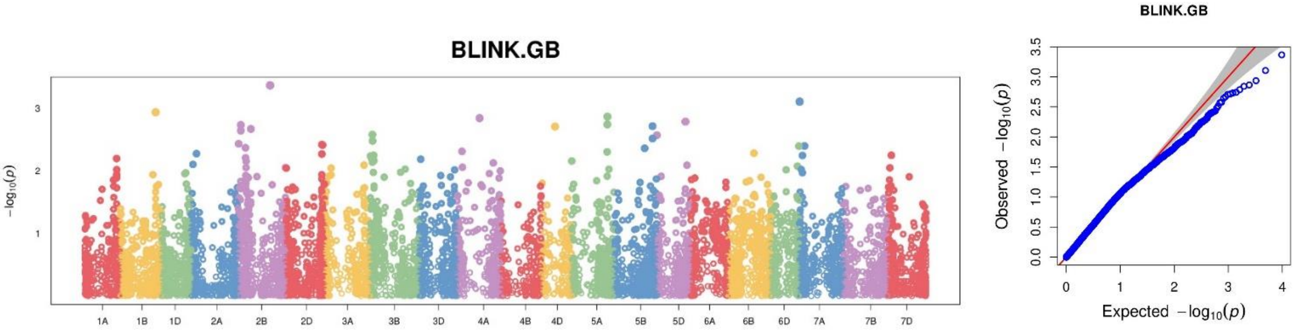

(c)

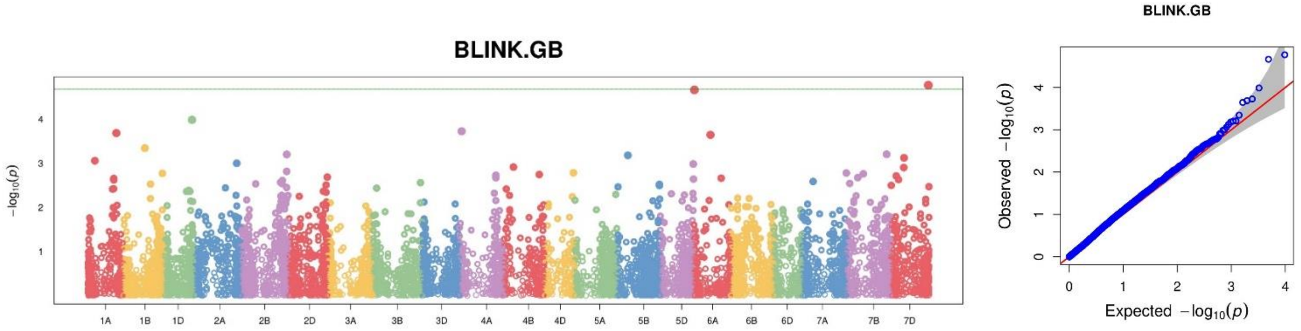

(d)

Fig S20 Manhattan plots and QQ plots for GB under (a) CTTS, (b) CATS, (c) CTLS, and (d) CALS

GB, grain breadth; CTTS, Conventional tillage timely sown; CATS, Conservation agriculture timely sown; CTLS, Conventional tillage late sown; CALS, Conservation agriculture late sown.

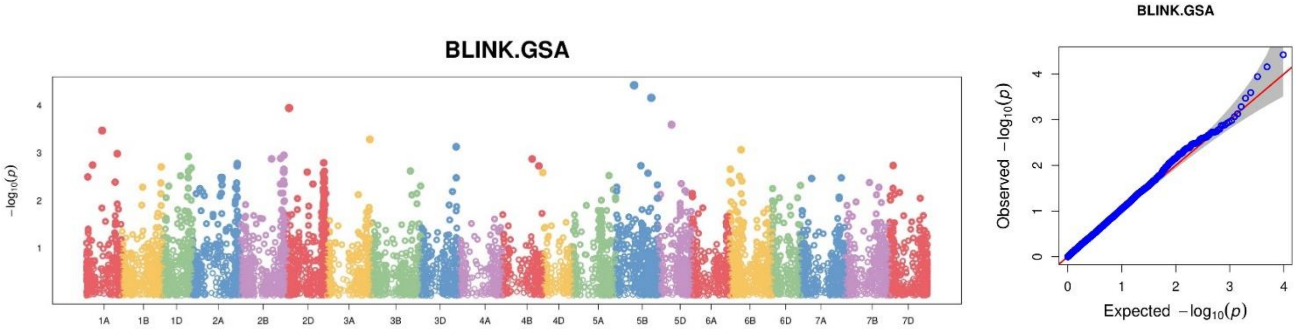

(a)

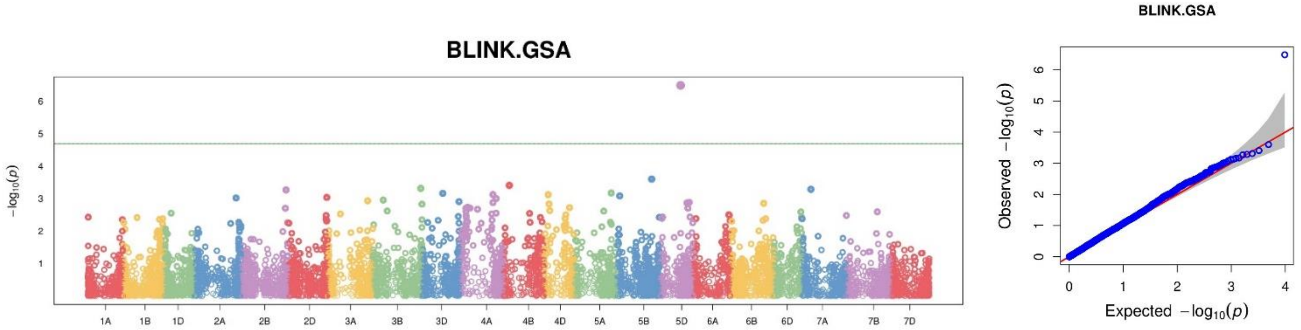

(b)

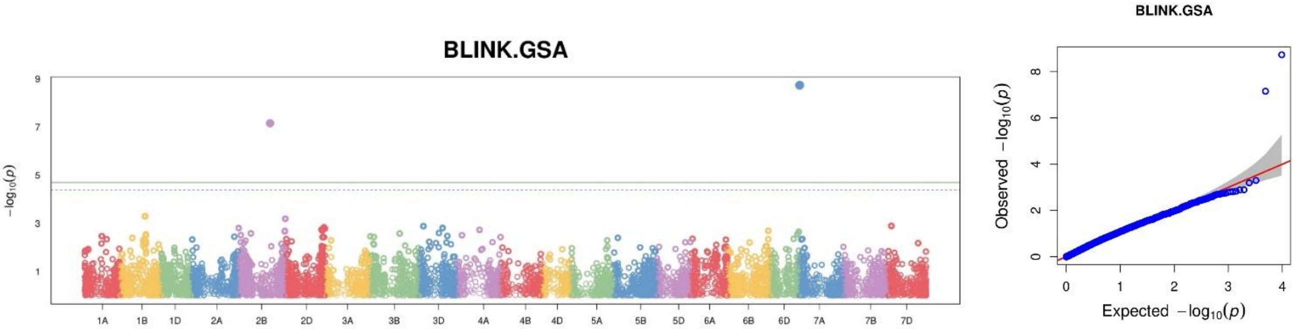

(c)

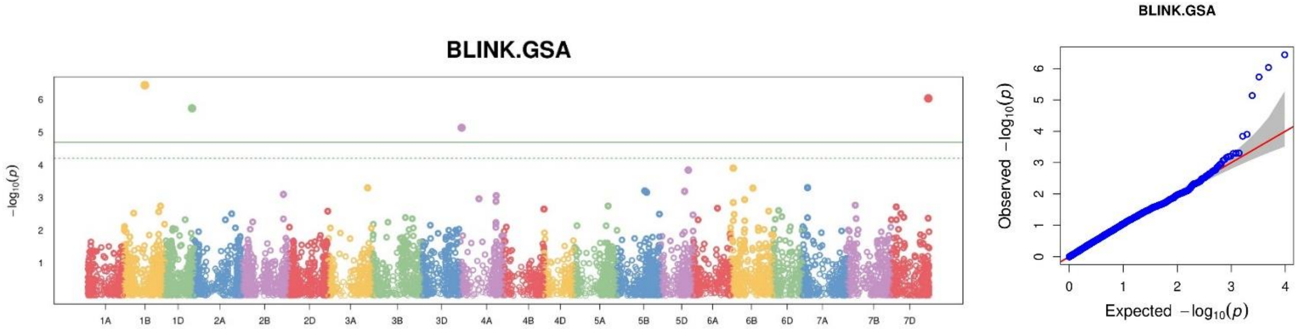

(d)

Fig S21 Manhattan plots and QQ plots for GSA under (a) CTTS, (b) CATS, (c) CTLS, and (d) CALS

GSA, grain surface area; CTTS, Conventional tillage timely sown; CATS, Conservation agriculture timely sown; CTLS, Conventional tillage late sown; CALS, Conservation agriculture late sown.
